# Test of the gravitational interaction between milligram masses using a miniature torsion pendulum

**DOI:** 10.1038/s41467-026-78187-4

**Published:** 2026-10-07

**Authors:** Zheng-Jie An, Wen-Hai Tan, Wen-Can Dong, Cheng-Gang Qin, Zu-Xing Deng, Zhi-Chao Duan, Du Chen, Yang Huang, Qi-Long Gong, Lin Zhu, Liang-Cheng Tu, Jun Luo, Shan-Qing Yang

**Affiliations:** 1 MOE Key Laboratory of TianQin Mission, TianQin Research Center for Gravitational Physics & School of Physics and Astronomy, Frontiers Science Center for TianQin, Gravitational Wave Research Center of CNSA, Sun Yat-sen University (Zhuhai Campus), Zhuhai, China; 2 MOE Key Laboratory of Fundamental Physical Quantities Measurement, Hubei Key Laboratory of Gravitation and Quantum Physics, PGMF and School of Physics, Huazhong University of Science and Technology, Wuhan, China; 3 Precision Measurement Research and Development Center, Sun, Yat-sen University Nanchang Research Institute, Nanchang, China

**Keywords:** General relativity and gravity, General relativity and gravity

## Abstract

Precision tabletop measurements of gravitational force are important for probing the fundamental nature of gravitation. Although gravitational experiments have spanned test masses from macroscopic objects to individual quantum systems, the mutual gravitational interaction between small—particularly microscopic—masses remains largely unexplored. Recent advances have extended such studies into the sub-100 mg regime, however, probing gravitation at still smaller masses continues to pose significant challenges. Here, we report a measurement of gravitational interaction between two 7.6 mg silicon wafers using a miniature Cavendish-type torsion balance combined with a rotating mass-modulation technique. Over separation distances from 0.654 to 1.554 mm, the measured forces agree with Newtonian predictions, achieving a maximum uncertainty of 50 aN (2*σ*) and enabling constraints on Yukawa-type deviations from Newtonian gravitation. From these data, we determine the gravitational constant *G* = 6.67(35) × 10^−11^m^3^ kg^−1^ s^−2^ (1*σ*). This work measured the gravitational interaction between milligram-scale objects, representing a significant step toward bridging the classical and quantum regimes of gravitation.

## Abstract (web summary)

Probing gravitation between small test masses continues to pose significant challenges. Here, the authors report a miniature closed-loop Cavendish-type torsion balance test using two 7.6 mg silicon masses, over separation distances from 0.654 to 1.554 mm and achieving a maximum uncertainty of 50 aN (2*σ*).

## Introduction

General relativity (GR) and the Standard Model (SM), the two cornerstones of modern physics, have withstood every experimental test to date. Yet their fundamental incompatibility provides a compelling impetus for probing the quantum nature of gravitation^1^. A large number of laboratory experiments have been performed to investigate the gravitational interactions, including measurements of the gravitational constant *G*^2,3^, tests of the deviation of the Newtonian inverse-square law at short ranges^4–9^, tests of the equivalence principle^10–12^, and other tests of the general relativistic effect^13–15^. In current experiments, to enhance the gravitational interaction signal, the source mass is typically chosen to be a massive object (including celestial bodies), while the test mass varies from macroscopic objects to individual quantum systems, depending on the experimental method and expected precision. However, gravitational measurements where both the source mass and the test mass are in the small-mass regime remain relatively scarce. Consequently, whether gravitational force deviates from Newton’s inverse-square law when the objects become sufficiently small or even enter the quantum regime remains a question worthy of investigation^16–20^. This work targets the largely unexplored gravitational interaction between small-mass objects, aiming to provide experimental evidence that may shed light on the nature of gravitation and lay a foundation for future studies on even smaller-mass systems^21^.

Probing gravitational interactions between even smaller source masses remains challenging due to the extreme weakness of gravitation compared to other fundamental interactions. Recent experiments have pushed the frontier toward smaller masses and novel designs. For instance, Jaffe et al.^22^ detected the gravitational interaction between freely falling cesium atoms and a centimeter-sized source mass of 0.19 kg. Westphal et al.^23^ measured gravitational coupling between two 90-mg gold spheres using a miniature torsion balance. Brack et al.^24^ demonstrated dynamical detection of gravitation between two meter-scale parallel beams in the hertz regime. Fuchs et al.^25^ reported an intermediate step toward detecting gravitation of small masses by using a 2.4-kg source mass and a magnetically levitated sub-milligram test mass.

Thanks to the development of the plasma deep silicon etching technology^26^, we have integrally fabricated a miniature silicon torsion pendulum and attractor (See Supplementary Note 1), which can address the challenges of precise geometric control in the manufacture and assembly. Here we report a precision measurement of the gravitational interaction between two milligram-mass silicon wafers (each 7.6 mg) using a closed-loop torsion balance, which achieves torque and force noise floors of approximately $0.15\,{{\rm{fNm}}}/\sqrt{{{\rm{Hz}}}}$ and $6\,{{\rm{fN}}}/\sqrt{{{\rm{Hz}}}}$, respectively, near the 30 mHz frequency band. Over test-to-source mass separations ranging from 0.654 to 1.554 mm, we compare our measured gravitational forces with Newtonian predictions and constrain possible Yukawa-type deviations from Newtonian gravitation. From these data, we determine the gravitational constant *G*.

## Results

### Experimental setup

**Fig. 1:**
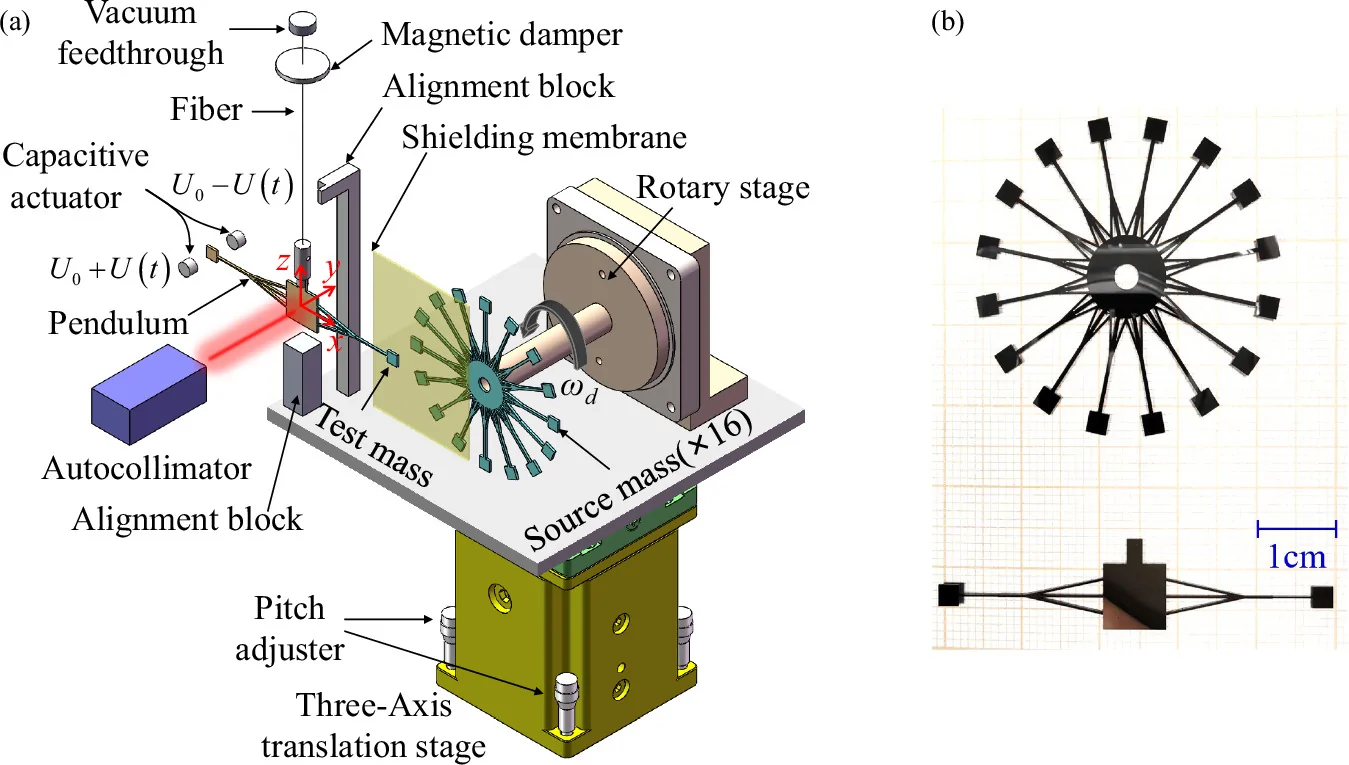
Experimental setup of the miniature torsion balance. **a** Schematic view of the experimental setup(not to scale). The pendulum is suspended by a 76-cm-long, 10-μm-diameter tungsten fiber, centered to the pendulum within 15 μm. The total mass of the pendulum, including the reflector and the fastenings, is about 243 mg. The eddy-current magnetic damper suspended by a 5-cm-long, 100-μm-diameter tungsten fiber is used to suppress the effect of environmental vibrations. The attractor, rotary stage and alignment block are all mounted on a three-axis translation stage to enables precise alignment with the pendulum. **b** The photograph of the pendulum and the attractor fabricated by the plasma deep silicon etching technology.

The schematic view of the experimental design is shown in Fig. 1. The miniature torsion pendulum consists of a symmetric structure with two silicon wafers positioned at its two ends, serving as the test mass and the counter-balance, respectively. The test mass has dimensions of 2.598 × 2.597 × 0.487 mm^3^ and a mass of approximately 7.6 mg (Table 1). The pendulum has an end-to-end length of 49.986 mm, with an average force arm of *L*_*eq*_ = 23.694 mm from the test mass center to the suspension fiber. A larger silicon wafer (8 × 8mm^2^) at the center of the pendulum acts as an autocollimator reflector for angular readout.

**Table 1:** 1*σ* contributions of error sources to the 16*ω*_*d*_ torque at *s* = 0.654 mm

Torque error	Measured	*δ*(*Δτ*_16*ω*_)	
sources	values	(aNm)	
Test mass			0.06
Length *x*	(2.598 ± 0.003)mm	0.01	
Thickness *y*	(0.4871 ± 0.0002)mm	< 0.01	
Width *z*	(2.597 ± 0.003)mm	0.01	
Density^c^	(2313.5 ± 11.9)kg/m^3^	0.06	
Source mass			0.01
Length *x*	(2.5883 ± 0.0007)mm	< 0.01	
Thickness *y*	(0.4985 ± 0.0003)mm	< 0.01	
Width *z*	(2.5878 ± 0.0008)mm	< 0.01	
Density^c^	(2313.5 ± 11.9)kg/m^3^	0.01	
Alignment			0.23
Position *dx*	± 0.024 mm	0.02	
Position *dy*	± 0.020 mm	0.23	
Attitude *θx*	± 130 μrad	< 0.01	
Attitude *θz*	± 130 μrad	< 0.01	
Driving system			0.10
Rotational speed	(0.6000 ± 0.0005)^∘^/s	0.08	
Run-out	≤3 μm	0.06	
Others^a^			< 0.01
Total Newtonian error			**0.26**
Other systematic errors			**0.46**
Electrostatic		0.27	
Magnetic		0.14	
Temperature		0.24	
Transfer function		0.22	
Voltage calibration		0.11	
Statistical error (with 100 hours integration time)			**0.27**
Total			**0.59**^**b**^

^a^Position errors *dx*, *dz* for both masses; alignment error *dz*; attractor wobble; attitude of the pendulum.

^b^The torque uncertainty of 0.59aNm (1*σ*) corresponds to a force uncertainty of 25aN (1*σ*) and 50aN (2*σ*) for an effective force arm of 23.694(3)mm. The bold style represents the emphasis for the root-sum-squared combined errors of related sub-items.

^c^The silicon wafer density value represents an average calculated from multiple samples cut from the same wafer used for both the torsion pendulum and source masses, determined by measuring their masses and dimensions.

The attractor is designed as a 16-fold symmetrical structure. It comprises 16 identical silicon wafer source masses arranged with azimuthal symmetry; each source mass has the same geometric dimensions as the test mass (as highlighted in Supplementary Fig. 1). The attractor, with a diameter of 38 mm and a thickness of 0.499 mm, is connected to a rotary stage via a shaft. Each source mass sequentially faces the test mass as the stage rotates at angular frequency *ω*_*d*_ (= 2*π* ⋅ *f*_*d*_), causing the torsion pendulum to experience a Newtonian gravitational signal at 16*f*_*d*_. Compared to the gap modulation method^5,23^, this design puts the signal frequency at the 16th harmonic of the rotation, far from the fundamental disturbance frequency, and allows the Newtonian force to be detected within the most sensitive frequency band of the torsion balance.

According to a strict alignment procedure (See Supplementary Note 2), the rotation axis of the attractor is aligned to the rotary stage within 40 μm (as highlighted in Supplementary Fig. 1). The rotation axis is aligned to the pendulum center within uncertainties of 24 μm and 20 μm in the *x* and *z* directions, respectively, while the face-to-face separation along *y* is determined with an uncertainty of below 20 μm. The perpendicularity of the rotation shaft relative to the attractor is monitored by an autocollimator and adjusted to be 1.86(13)mrad (as highlighted in Supplementary Fig. 2).

**Fig. 2:**
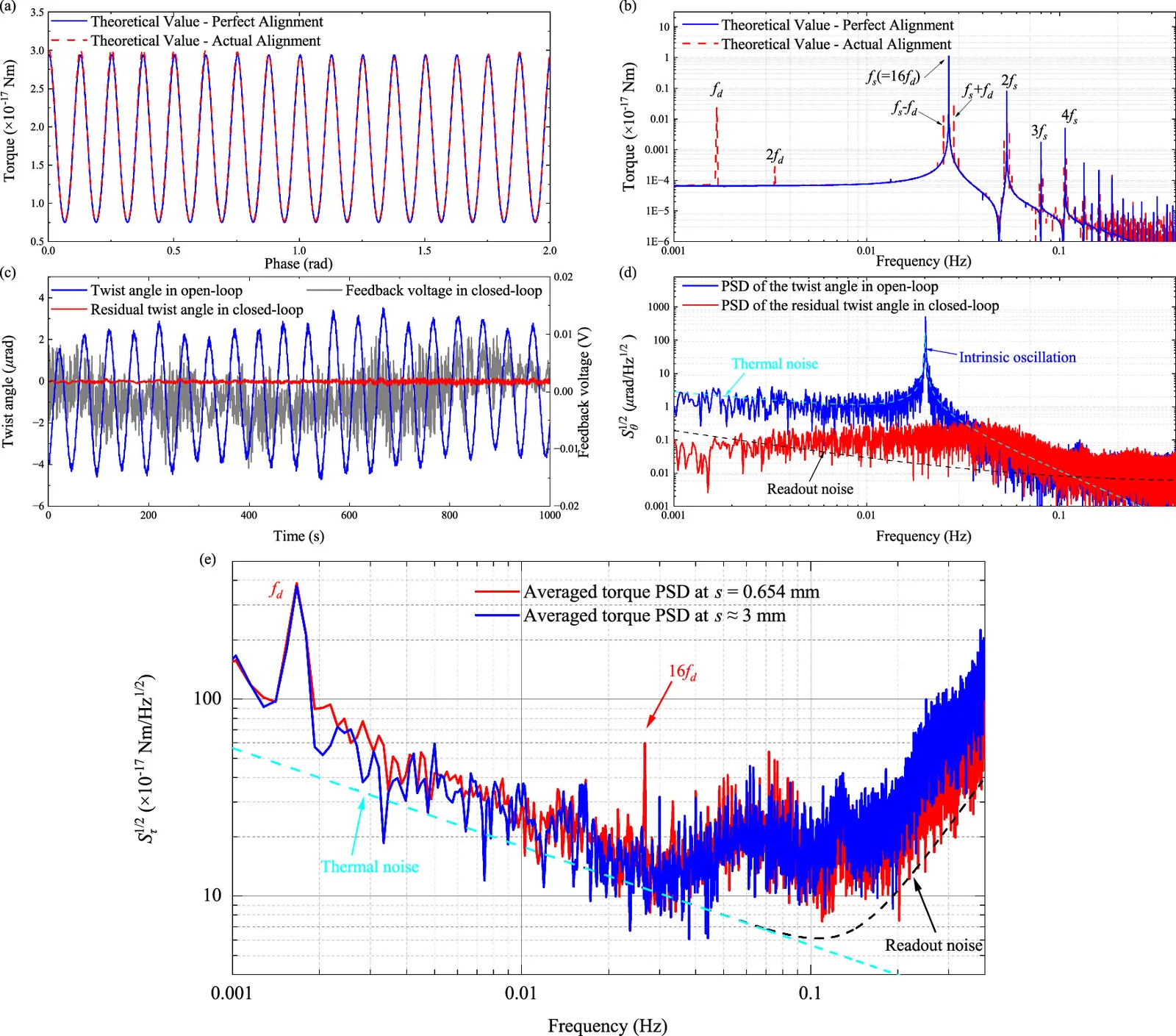
Experimental data. **a** Comparison of the calculated gravitation-induced torque with perfect alignment and actual alignment of the test and source masses when the separation is 0.654 mm. **b** The amplitude spectral density of the calculated gravitation-induced torque, where the harmonics are attributed to the structural design and actual dimension errors of test and source masses. The theoretical calculation includes contributions from all 16 source masses and their supporting spokes; the spokes alone contribute 0.53 aNm to the 16*f*_*d*_ torque at *s* = 0.654 mm, which corresponds to about 4.7% of the total calculated Newtonian torque of 11.17(26) aNm. **c** Typical time-domain measurement of the open-loop twist angle, the closed-loop feedback voltage, and the residual twist angle. **d** The corresponding power spectral density (PSD) of the torsional angle measured before and after controlled with data lengths of 40,000 s, where the feedback system suppresses the residual twist angle to a level below the thermal noise for the free oscillation (for *f*≤30 mHz) due to the increased stiffness. **e** Averaged torque PSD derived from six independent feedback voltage segments (7800 s each) at a separation of 0.654 mm. A distinct gravitational signal is observed at 16*f*_*d*_. For comparison, an identical measurement was performed at a larger separation (*s* ≈ 3 mm), where the Newtonian gravitational torque at 16*f*_*d*_ is theoretically expected to fall below the torsion balance noise floor and thus remain undetectable in the power spectrum. The measurement confirms this expectation: a prominent signal appears only at the drive fundamental frequency *f*_*d*_, with a magnitude consistent with the close-separation result, while no discernible signal is present at 16*f*_*d*_. This control test further corroborates the reliability of the close-separation measurements.

To suppress the electrostatic disturbances and optimize the signal-to-noise ratio, the closest face-to-face separation between the test and the source mass is set to *s* = 0.654 mm. A 50-μm-thick, gold-coated monocrystalline silicon membrane is inserted between them to prevent direct electrostatic coupling. The source-mass-to-membrane separation is fixed at 0.268(3) mm, resulting in a pendulum-membrane gap ≥0.336 mm. The Casimir effect^27^ and the squeeze-film damping effect^28,29^ between the pendulum and the shielding membrane are negligible due to the large separation (See Supplementary Note 3). The Newtonian torque between two masses can be calculated through numerical integration, and as an example, the calculated result with a separation of 0.654 mm is shown in Fig. 2a, b.

Although both the pendulum and the membrane are grounded, a non-zero residual electrostatic potential difference still exists due to the patch effect^6,30,31^, introducing a negative stiffness that compromises pendulum stability at small separations. To address this, two differential capacitive actuators (with a separation of  ~ 21.5 mm, positioned on both sides of the counter-balance wafer) with a proportional-integral-derivative (PID) feedback algorithm are utilized to provide weak rotational control^32^. The closed-loop system maintains the pendulum in an untwisted state during the experiment, with the feedback voltage *U*(*t*) directly reflecting the torque acting on the pendulum.

The entire system is installed inside a vacuum chamber maintained at approximately 10^−5^Pa by an ion pump. The torsional motion of the pendulum is monitored by the autocollimator with a noise floor of about $10\,{{\rm{nrad}}}/\sqrt{{{\rm{Hz}}}}$ in the frequency band of tens of mHz (as highlighted in Supplementary Fig. 3). To characterize the pendulum’s mechanical properties, the pendulum-membrane separation was first set to about 6mm for negligible electrostatic effect. The free oscillation of the pendulum with a large amplitude ( ~ 3 mrad) is recorded, yielding the natural period *T*_0_ = 49.50(5)s and the quality factor *Q* = 1786(20) due to the intrinsic dissipation. The moment of inertia of the pendulum is calculated to be *I* = 12.61(1) × 10^−9^kg ⋅ m^2^ from the measured geometric parameters (as highlighted in Supplementary Fig. 1). Then the torsional spring constant of the fiber is determined to be ${k}_{f}=I{(2\pi /{T}_{0})}^{2}=203.1(5)\,{{\rm{pNm/rad}}}$. For a higher-precision test of sensitivity, small-amplitude ( ~ 6 μrad) oscillation of the pendulum is usually maintained, as shown in Fig. 2c, d. Averaged torque power spectral density (PSD) of the closed-loop system at a test-to-source mass separation of 0.654 mm is shown in Fig. 2e.

### Measurements of gravitation

**Fig. 3:**
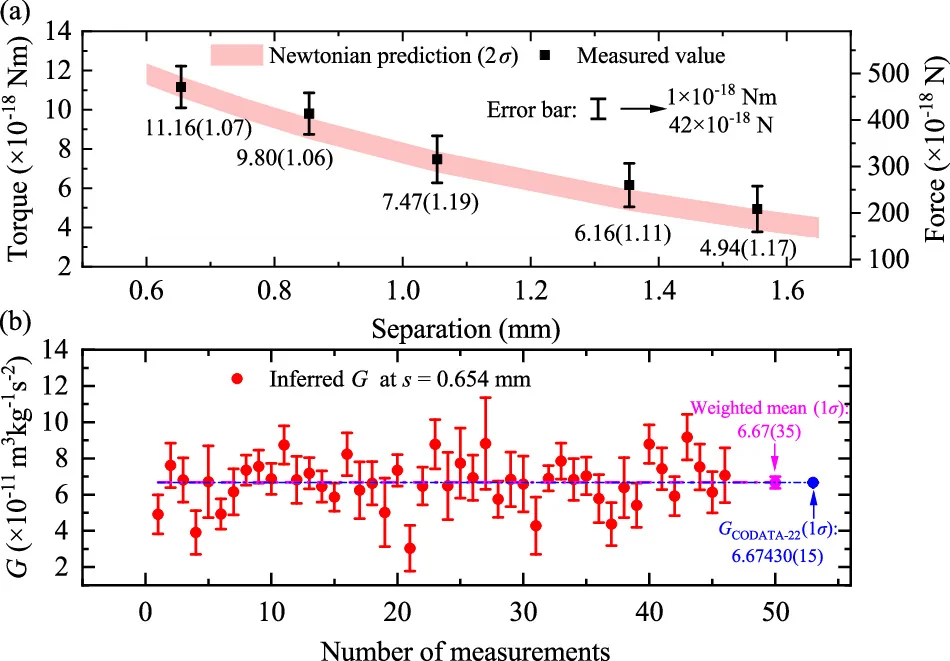
Measured gravitational forces and inferred gravitational constant G. **a** Comparison of experimental results and the theoretical calculations of the Newtonian gravitation ranging from 0.654 to 1.554 mm with 2*σ* confidence level. The red band represents the theoretical Newtonian torque calculated from the measured geometric parameters of the pendulum and the source mass, while the black squares denote the measured torque at different separations, with the error bars incorporating both the statistical and other systematic errors summarized in Table. 1. The measured torque values are indicated near the data points. **b** Gravitational constant *G* inferred from the measured torque at a separation of 0.654 mm. Each red circle represents the *G* value obtained from a single measurement (46 measurements in total), with each measurement corresponding to 13 periods ( ~ 7800 s) of source mass rotation, the error bars reflect only statistical uncertainty. The magenta circle represents the *G* value inferred from the weighted mean of torque accumulated over 100 h of integration time, with error bars incorporating both statistical and systematic uncertainties. For reference, the CODATA recommended *G* value is shown as a blue circle. The relative error of the *G* value obtained in this work is evaluated at the 1*σ* confidence level for comparison with the CODATA recommendation.

Using the high-sensitivity torsion balance with the rotating mass-modulation technique, the gravitational coupling between two 7.6 mg silicon wafers is characterized with a combined uncertainty of 1.2 aNm (2*σ*) in torque and 50 aN (2*σ*) in force. The measured gravitational forces at a series of separations are summarized in Fig. 3a, which demonstrate the consistency of the experimental results with Newton’s law of gravitation.

Experiments with small-mass objects are not generally competitive for improving the accuracy of classical gravitational experiments, such as the *G* measurement, tests of the deviation of the Newtonian inverse-square law, and so on, due to the smaller gravitational effect between small masses. Even so, the *G* value can be inferred from the measured gravitational force as $G={F}_{\exp }/{{\mathcal{M}}}$, where ${{\mathcal{M}}}=\int_{{V}_{1}}\int_{{V}_{2}}\frac{{\rho }_{1}({{{\boldsymbol{r}}}}_{1})\,{\rho }_{2}({{{\boldsymbol{r}}}}_{2})}{| {{{\boldsymbol{r}}}}_{2}-{{{\boldsymbol{r}}}}_{1}{| }^{2}}\,d{V}_{1}\,d{V}_{2}$ denotes the geometric factor. The relative errors in Fig. 3a become larger as the separation increases because the gravitational interaction weakens, while disturbances from electromagnetism, seismic noise, and other random noise remain almost the same. Therefore, the result of ${F}_{\exp }$ at the closest separation of 0.654 mm is utilized to obtain the *G* value, where ${F}_{\exp }=(471\pm 23)\,{{\rm{aN}}}$ (1*σ*), ${{\mathcal{M}}}=(7.06\pm 0.16)\times 1{0}^{-6}\,{{{\rm{kg}}}}^{2}/{{{\rm{m}}}}^{2}$, and *G* is deduced to be 6.67(35) × 10^−11^ m^3^ kg^−1^ s^−2^ (1*σ*), agreeing with the CODATA-2022 value^3^ of 6.67430(15) × 10^−11^ m^3^ kg^−1^ s^−2^ within the 1*σ* uncertainty of this measurement (5.3% relative uncertainty), as shown in Fig. 3b).

To investigate possible frequency dependence of the measurement, we extended the turntable rotation period from 600 to 3600 s, corresponding to a gravitational signal frequency of 4.444 mHz at the 16th harmonic. This frequency selection was motivated by two considerations: (1) sensitivity analysis—despite the 1/*f*-type noise profile of our torsion balance increasing noise floors at this frequency compared to the previous setting, it remains within acceptable limits; (2) frequency separation—maximizing the separation from the current 16*f*_*d*_ (26.667 mHz) to enable detection of potential frequency-dependent phenomena. We performed 15 measurement sets with a test-to-source mass spacing of 0.654 mm, each comprising 13 full turntable rotations (13 h). Using the same signal extraction method (See Supplementary Note 4), we measured *τ*_2_ = (11.41 ± 0.46)aNm at 4.444 mHz. Within 1*σ* uncertainty, this result agrees with our previous measurement *τ*_1_ = (11.16 ± 0.27)aNm (Table 1) at 26.667 mHz (statistical uncertainty only), confirming no frequency-dependent phenomena were observed (as highlighted in Supplementary Fig. 4).

### Constraints on Yukawa interactions

**Fig. 4:**
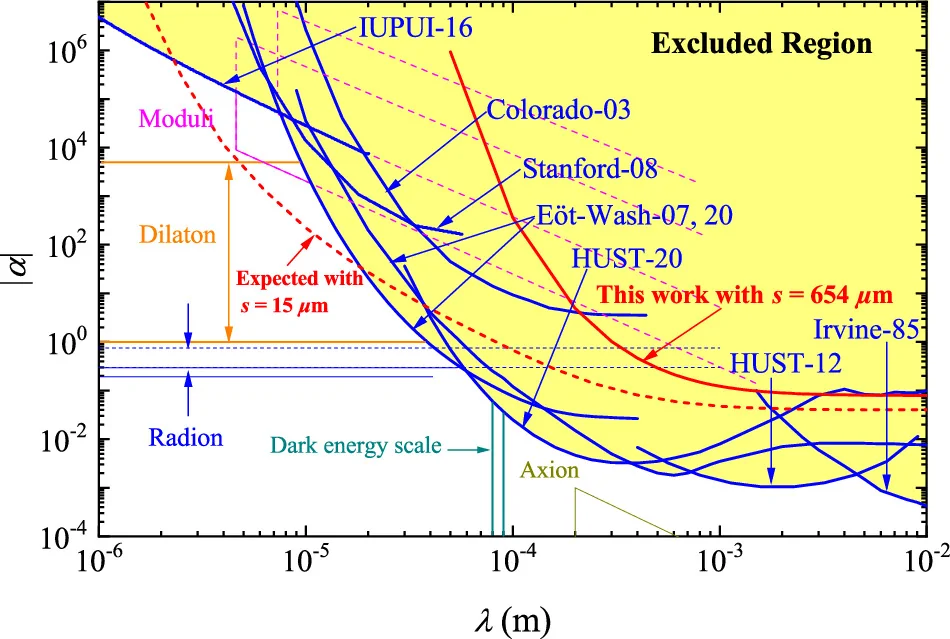
Constraints on Yukawa-type violations of the Newtonian 1/*r*^2^ law. The shaded regions are excluded at the 95% confidence level by previous experiments from refs. ^4–9,47,48^, and this work (the red solid line), respectively. Light lines show various theoretical predictions summarized in ref. ^34^. If the separations between smaller masses can be decreased to  ~ 15 μm, while the sensitivity of the torsion balance and other systematic effects can still be maintained at the same level, the expected constraint on the *α* is also show in red dashed line.

Similarly, we can also impose constraints on the *α*–*λ* parameter space in the context of extra-dimension models by using the Yukawa-type potential $V=-\frac{G{m}_{1}{m}_{2}}{r}(1+\alpha {e}^{-r/\lambda })$^33,34^ as shown in Fig. 4. The resulting bound is about two orders of magnitude weaker than that obtained in our previous work^5^, where two 8.7 g tungsten blocks were used as test and source masses at millimeter range. If the separations between smaller masses in this work can be decreased to  ~ 15 μm, while the sensitivity of the torsion balance and other systematic effects can still be maintained at the same level, the constraints on the *α*–*λ* parameters could be improved at *λ* ~ 10 μm.

## Discussion

Suggestions based on two experimental schemes^18,35^, to observe gravitation-induced quantum effects, both the source mass and the test mass should ideally fall within the range of 10^−14^ to 10^−12^ kg, representing masses significantly smaller than the Planck mass (*m*_*p*_ ≈ 2.2 × 10^−8^ kg). Within this mass window, gravitational interactions between micrometer-scale objects can produce measurable entanglement within experimentally achievable coherence times^17^. This target mass range presents a substantial challenge beyond the current scope of our work, yet aligns with the scale that experimental efforts are advancing toward^21^. It is essential to improve sensitivity and reduce the thermal noise of the torsion balance by reducing the fiber’s *k*_*f*_/*Q* ratio^36^. For example, by using a quartz fiber with a diameter of 3 μm (*k*_*f*_ ~ 10^−13^ Nm/rad) and assuming a quality factor *Q* ~ 25,000, the sensitivity of the torsion balance can be enhanced by approximately a factor of 90 compared to this work. At the same time, by shortening the separation from the current 654 μm to  ~ 15 μm, a 10^5^ improvement in signal-to-noise ratio can be obtained for probing Yukawa-type deviations of the Newtonian 1/*r*^2^ law at *λ* ≈ 90 μm (See Supplementary Note 5). Furthermore, with a 30-day integration period, the optimized torsion balance is expected to achieve a torque measurement resolution of  ~ 10^−21^ Nm. This would potentially enable the detection of gravitational coupling between two objects separated by tens of micrometers and below the Planck mass scale, representing a significant step toward measuring gravitational interactions between smaller mass objects. In recent years, a growing number of experimental platforms have pursued the reduction of test-mass scales in the quest for gravitational interaction detection between objects in the quantum regime. These include torsion pendulum techniques, optical and magnetic levitation schemes, as well as cavity optomechanical systems and so on^21,25,37–43^. The continued advancement of these approaches, each operating across different but increasingly overlapping mass regimes, holds promise that gravitational interactions between quantum-state objects may be observed in the foreseeable future. Nevertheless, the fact that gravitation can be both sourced and probed in a yet unexplored low-mass regime is very important, but how to overcome various disturbances and maintain the quantum coherence poses a completely different technological challenge.

## Methods

### Kinematic equation

In the closed-loop system, the feedback voltages *U*_0_ + *U*(*t*) and *U*_0_ − *U*(*t*) are applied to two electrodes (*U*_0_ = 5V), respectively. The equation of torsional motion of the closed-loop torsion pendulum is

1 $$I\ddot{\theta }+{k}_{f}(1+i/Q)\theta -{k}_{e}\theta=\tau -\beta U,$$

where *θ* is the torsional angle, *k*_*e*_ is the electrostatic stiffness introduced by the shielding membrane, *τ*(*t*) is the external torque, including the thermal noise torque of the suspension fiber, *β* is the ratio of the torque to the feedback voltage *U*(*t*), which is calculated as $U(t)={k}_{p}\theta+{k}_{i}\int\theta \,{{\rm{d}}}t+{k}_{d}\dot{\theta }$ according to the PID algorithm, with *k*_*p*_ = 0.02V/μrad, *k*_*i*_ = 0.002V/(μrad ⋅ s), *k*_*d*_ = 0.1V/(μrad ⋅ s^−1^) are the proportional, integral, and differential term, respectively. The measured torque *τ* at the angular frequency *ω* is calculated by the transfer function derived from Eq. (1) as

2 $$\begin{array}{ll}&{H}_{U}(\omega )\equiv \dfrac{U(\omega )}{\tau (\omega )}=\dfrac{U(\omega )}{\theta (\omega )}\dfrac{\theta (\omega )}{\tau (\omega )}\\ &=\dfrac{\left({k}_{p}+\dfrac{{k}_{i}}{i\omega }+i\omega {k}_{d}\right)\Delta \phi }{({k}_{f}-{k}_{e}-I{\omega }^{2})+i\dfrac{{k}_{f}}{Q}+\beta ({k}_{p}+\dfrac{{k}_{i}}{i\omega }+i\omega {k}_{d})\Delta \phi },\end{array}$$

where Δ *ϕ* = *e*^−iω Δt^ is the correction for the delay between the input and the output signal with Δ*t* = 1s the sampling interval. A typical voltage curve *U*(*t*) is shown in Fig. 2c. The feedback gain is determined to be *β* = 33.4(5)fNm/V (as highlighted in Supplementary Fig. 5), and the electrostatic stiffness is *k*_*e*_ = 19.4(2.7)pNm/rad at *s* = 0.654mm (as highlighted in Supplementary Fig. 6). The typical value of relative uncertainty of *H*_*U*_(*ω*_*s*_) is estimated to be about 2%, primarily arising from the uncertainties of *β* and *k*_*e*_.

### Data analysis

In the measurement, the rotation period of the attractor is set to 600s so that its 16th harmonic (26.667 mHz) would fall within the lowest noise band of the torsion pendulum. The angular position of the attractor is monitored by an encoder integrated inside the rotary stage (as highlighted in Supplementary Fig. 7). The gravitational coupling between the test and source masses is measured at the separations of *s* = 0.654, 0.854, 1.054, 1.354, and 1.554 mm, respectively. Each data run at different separations lasts about 40  ~ 100 h to obtain an acceptable statistical error. Fig. 2e shows the typical torque power spectral density (PSD) of the closed-loop torsion pendulum. The 16*ω*_*d*_ component of the feedback voltage *U*(*ω*_*s*_), is extracted via ${U}_{a}=\frac{2}{T}\int_{0}^{T}U(t)\cos ({\omega }_{s}t)\,{{\rm{d}}}t$ and ${U}_{b}=\frac{2}{T}\int_{0}^{T}U(t)\sin ({\omega }_{s}t)\,{{\rm{d}}}t$, after removing the low-frequency drift using a third-order polynomial fit. The measured torque *τ*(*ω*_*s*_) is then calculated as *τ*(*ω*_*s*_) = *U*(*ω*_*s*_)/*H*_*U*_(*ω*_*s*_) (See Supplementary Note 4).

### Newtonian gravitation

The 16*f*_*d*_ Newtonian torque is calculated via numerical integration of the Newtonian gravitational interaction over all volume elements of the torsion pendulum assembly and all 16 source masses with their supporting struts, using the measured geometries and densities of each component. Individual integrations are performed for each separation from *s* = 0.654 to 1.554 mm. The main error sources in the calculation are from the dimensions, positions, orientations, densities, and alignments of the test and source masses. Combining the uncertainties of these parameters yields a Newtonian gravitation of *τ*_*N*_ = (11.17 ± 0.26)aNm (1*σ*) in torque and *F*_*N*_ = (471 ± 11)aN in force at *s* = 0.654 mm. The main parameters and their contributions to the Newtonian torque error budget are summarized in Table 1, while the error bands at different separations are shown in Fig. 3a.

### Electrostatic effect

Electrostatic interactions between the pendulum and the membrane, arising from surface patch potentials, could produce a spurious signal at 16*f*_*d*_, as well as contribute to random noise. Fluctuations in the patch potential manifest as random noise^30,31^. Furthermore, mechanical vibrations of the rotating stage can induce variations in the pendulum-membrane separation distance. This separation change modulates the capacitance and consequently introduces parasitic electrostatic torque^6^. The shielding membrane translational displacement along the gap direction was precisely measured using an ultra-precision laser interferometer, yielding a maximum displacement of *δs*≤324 pm. With the measured electrostatic stiffness *k*_*e*_ = 19.4(2.7) pNm/rad, the resulting parasitic electrostatic torque noise is estimated as *δτ*_*e*_ = *k*_*e*_ ⋅ *δs*/*L*_*eq*_ ≤ 0.27 aNm (as highlighted in Supplementary Fig. 6).

### Magnetic effect

Magnetic coupling between the test and the source masses, arising from both the remanent magnetic moment and induced moment by ambient magnetic fields, could produce a spurious signal at 16*f*_*d*_ that mimics the Newtonian torque. The magnetic torque is modeled by integrating the dipole-dipole interaction energy^44^ as:

3 $${\tau }_{m}=\dfrac{\partial }{\partial \theta }\iint \dfrac{{\mu }_{0}}{4\pi {r}^{3}}{\vec{M}}_{p}\cdot \left[3\hat{r}({\vec{M}}_{a}\cdot \hat{r})-{\vec{M}}_{a}\right]\,{{\mbox{d}}}{V}_{p}\,{{\mbox{d}}}\,{V}_{a},$$

where ${\vec{M}}_{p}$ and ${\vec{M}}_{a}$ denote the magnetization intensity (magnetic moment per unit volume) of the pendulum and attractor, which are originated either from the remanence ${\vec{M}}_{r}$ or are induced by an external field as ${\vec{M}}_{i}=\chi \vec{B}/{\mu }_{0}$. The remanent magnetization of the silicon sample was measured using a high-sensitivity atomic magnetometer within a near-zero magnetic field environment^45^, yielding ${\vec{M}}_{rp}={\vec{M}}_{ra}=(2.4,3.9,5.6)\,{{\rm{mA/m}}}$ along the *x*, *y*, and *z* directions (the coordinate axes are shown in Fig. 1a), respectively. Using the volume magnetic susceptibility of silicon^46^ (*χ*_Si_ = − 3.2 × 10^−6^) and the ambient magnetic field measured at the pendulum’s location, the induced magnetization intensity is estimated to be ${\vec{M}}_{ip}={\vec{M}}_{ia}=(0.1,7.9,4.1)\,{{\rm{\mu A/m}}}$. Based on the measured and estimated values of magnetization intensity, the magnetic torque at *s* = 0.654 mm calculated from Eq. (3) is *δτ*_*m*_ < 0.14 aNm. Furthermore, a pair of horizontally orthogonal coils generated controlled alternating magnetic fields to characterize the torsion balance response. When combined with adjacent magnetic field fluctuations at 16*f*_*d*_ frequency, the magnetic torque noise from horizontal magnetic field variations is estimated to be less than 0.02 aNm (as highlighted in Supplementary Fig. 8).

### Temperature effect

Several experimental elements are sensitive to environmental temperature, including thermoelastic behavior of the suspension fiber, drift of the equilibrium position, and change of the geometries due to thermal deformation. To estimate these effects, the ambient temperature is intentionally modulated via the air conditioning system, yielding a temperature response coefficient of the torsion pendulum of *k*_*T*_ = ( − 114.2 ± 5.7) fNm/^∘^C. Combined with the typical temperature fluctuation of *δT*≤2.1 μK at 16*f*_*d*_ around the pendulum’s location during an experimental run of 7800 s, the resulting torque uncertainty is estimated to be *δτ*_*T*_ = *k*_*T*_ ⋅ *δT* < 0.24 aNm (as highlighted in Supplementary Fig. 9).

## Supplementary information

Supplementary Information

Transparent Peer Review file

## Data Availability

The data that support the findings of this study are available from the corresponding author upon request.

## Code availability

The custom codes used for data acquisition and analysis are available from the corresponding author upon request.
