# Supplementary material for: Test of the gravitational interaction between milligram masses using a miniature torsion pendulum
Source: Nat Commun. 2026 Oct 7;17:11425. doi: 10.1038/s41467-026-78187-4 (PMC13737168; doi:10.1038/s41467-026-78187-4)
Supplement: Supplementary file 1 [file 41467_2026_78187_MOESM1_ESM.pdf]

Supplementary Information of
"Test of the gravitational interaction between milligram masses using a miniature torsion pendulum"

Zheng-Jie An, Wen-Hai Tan, Wen-Can Dong, Cheng-Gang Qin, Zu-Xing Deng, Zhi- Chao Duan, Du Chen, Yang Huang, Qi-Long Gong, Lin Zhu, Liang-Cheng Tu, Jun Luo, Shan-Qing Yang

This supplementary information provides detailed descriptions of the Signal extraction method, Alignment procedure, Fabrication Process, Evaluation of residual gas effects, and Sensitivity estimation in the main text.

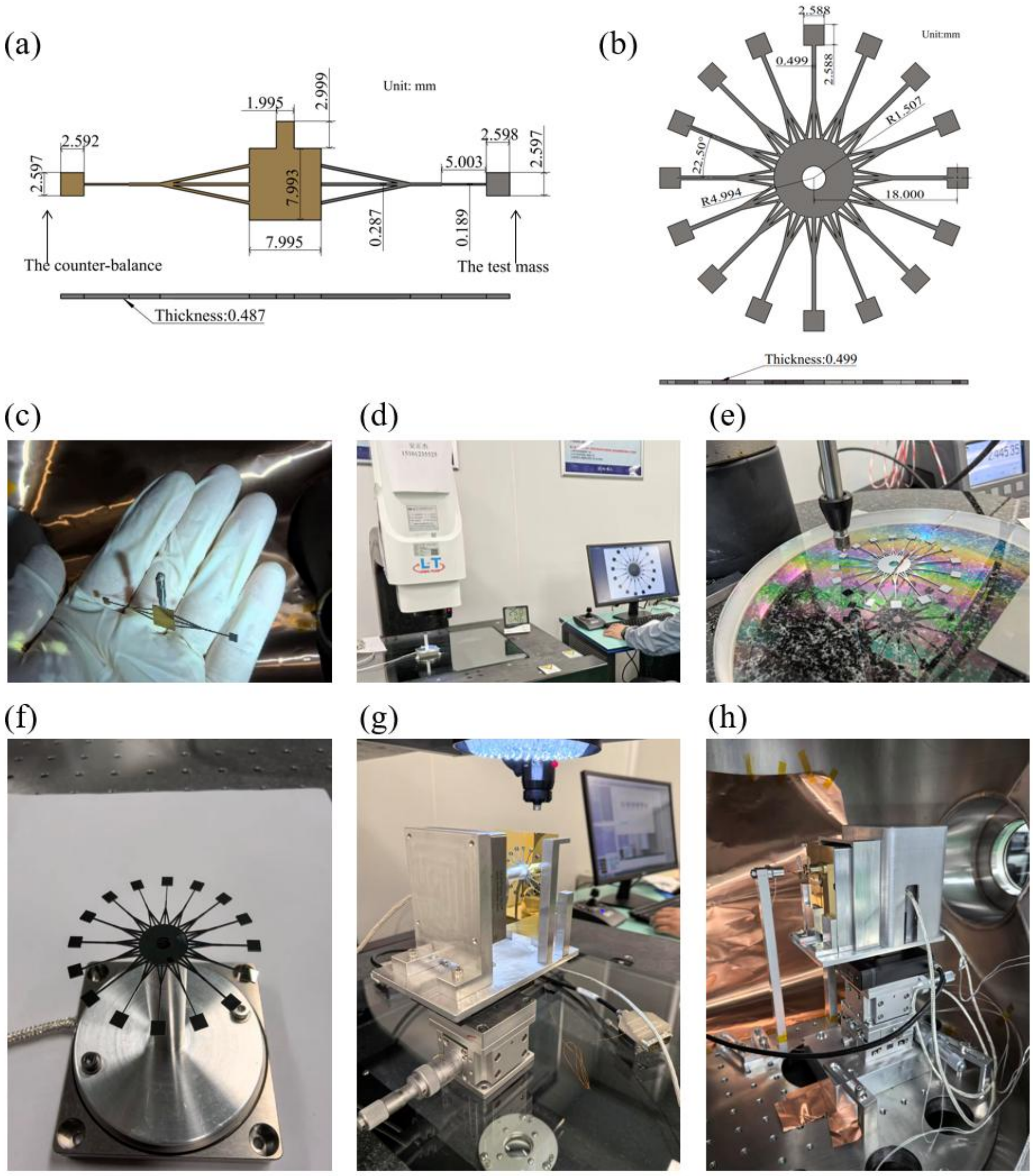

Supplementary Figure 1 Characterization of the torsion pendulum and attractor assembly. (a) Measured dimensions of the torsion pendulum. The 0.487-mm-thick pendulum has an end-to-end length of 49.986 mm and features a symmetric structure: two 2.598 × 2.597 × 0.487 mm^3 silicon wafers at its ends serve as the test mass and the counterbalance, respectively. (b) Measured dimensions of the 16-fold symmetric attractor, which comprises 16 identical silicon wafers, each with 2.588 × 2.588 × 0.499 mm^3. (c) Photograph of the torsion pendulum suspended by a tungsten fiber inside the vacuum chamber. (d) Photograph of the coaxiality measurement of the attractor disc using a Video Measuring Machine (QVP5040T, accuracy: ±3 μm); the rotation axis is aligned to the rotary stage within 40 μm. (e) Photograph of the Contact Length Gauge (HEIDENHAIN CT6001, accuracy: ±0.1 μm) measuring the thickness of the attractor disc. (f) Photograph of the attractor disc mounted onto the rotary stage via the rotation shaft. (g) Photograph of the Video Measuring Machine measuring the separation between the attractor disc and the shielding membrane, as well as the relative position between the rotation shaft and the alignment reference block. (h) Photograph of the complete attractor assembly installed inside the vacuum chamber.

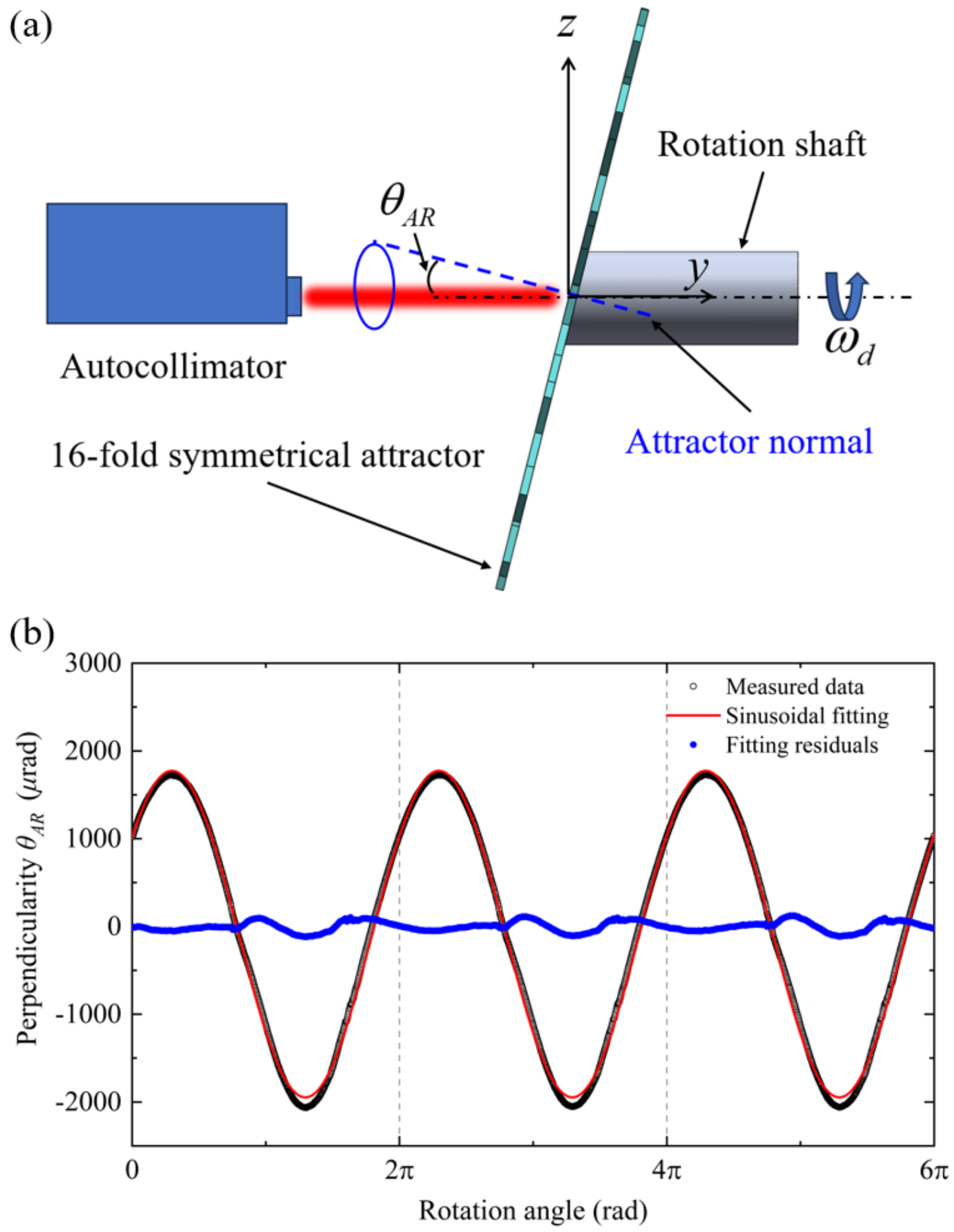

Supplementary Figure 2 Measurement of rotation-shaft-to-attractor perpendicularity. (a) Schematic view of the measurement method. When the rotation shaft is non-perpendicular to the attractor surface, the autocollimator-measured angle varies with attractor rotation. (b) The measured angular deviation θ_{AR} varies with the attractor's rotation angle. The data are fitted to a sinusoidal function at rotation frequency, yielding a non-perpendicularity of 1.86 mrad. This value is correspondingly corrected in the theoretical calculation of Newtonian gravitation. Additionally, the fitting residuals (blue dots) are determined to be less than ±130 μrad, introducing a < 0.01 aNm calculation error to the Newtonian torque.

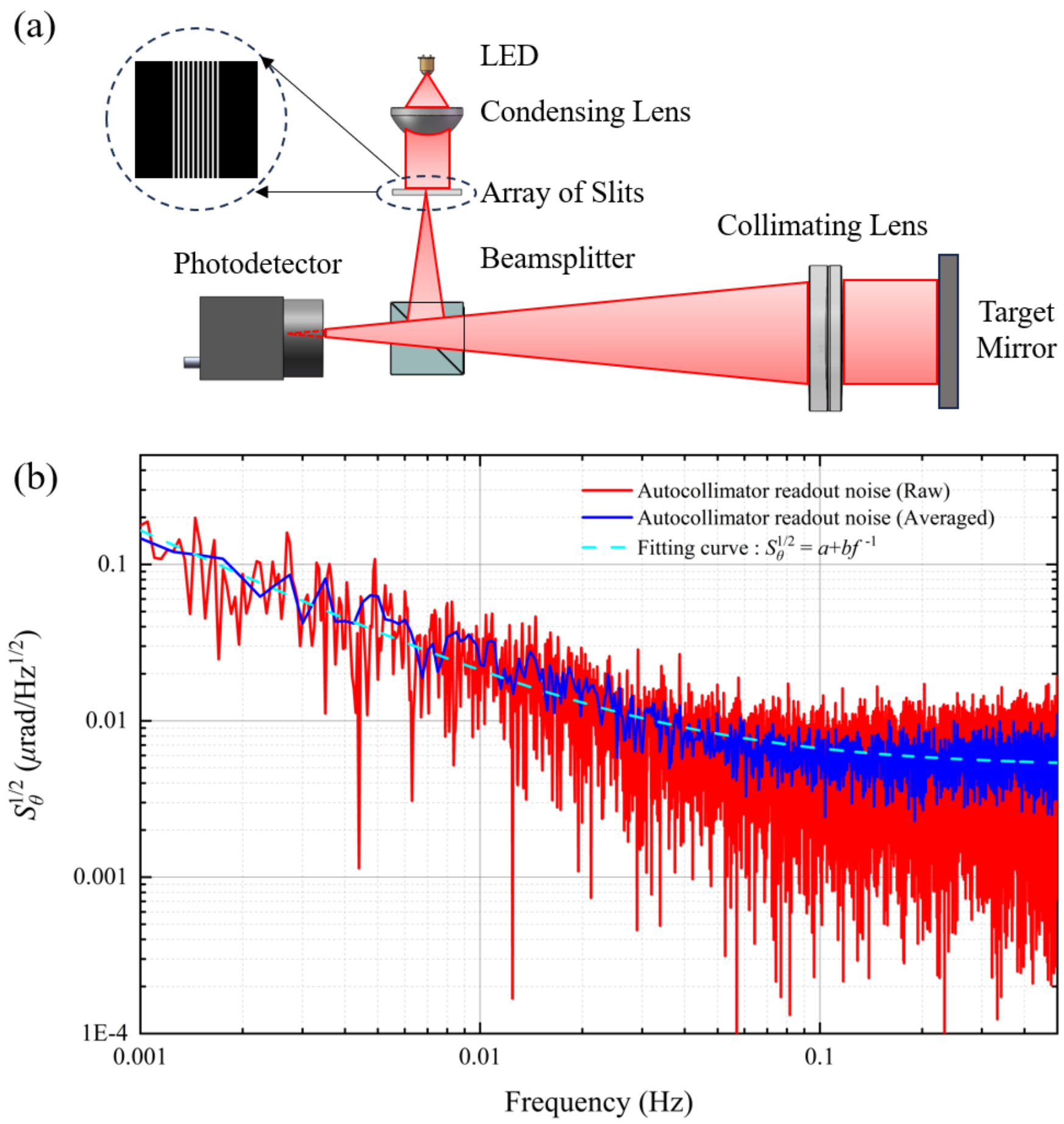

Supplementary Figure 3 Characterization of the autocollimator. (a) Schematic diagram of the autocollimator used in this work. (b) Readout noise floor of the autocollimator. During noise floor measurement, a fixed mirror replaces the torsion pendulum for beam reflection. The red curve shows the raw power spectral density with a data length of 20,000 s, while the blue curve displays the five-segment averaged result, reducing estimation variance and smoothing the noise floor. The angular power spectral density reaches $\sim 10\ \mathrm{nrad}/\sqrt{Hz}$ in the frequency band of interest (20 – 30 mHz). The fitted curve (cyan dashed line) follows $S_\theta^{1/2} = a + bf^{-1}$, with coefficients $a = 5.07(6)\ \mathrm{nrad}/\sqrt{\mathrm{Hz}}$ and $b = 0.1602(6)\ \mathrm{nrad}\cdot\mathrm{Hz}/\sqrt{\mathrm{Hz}}$.

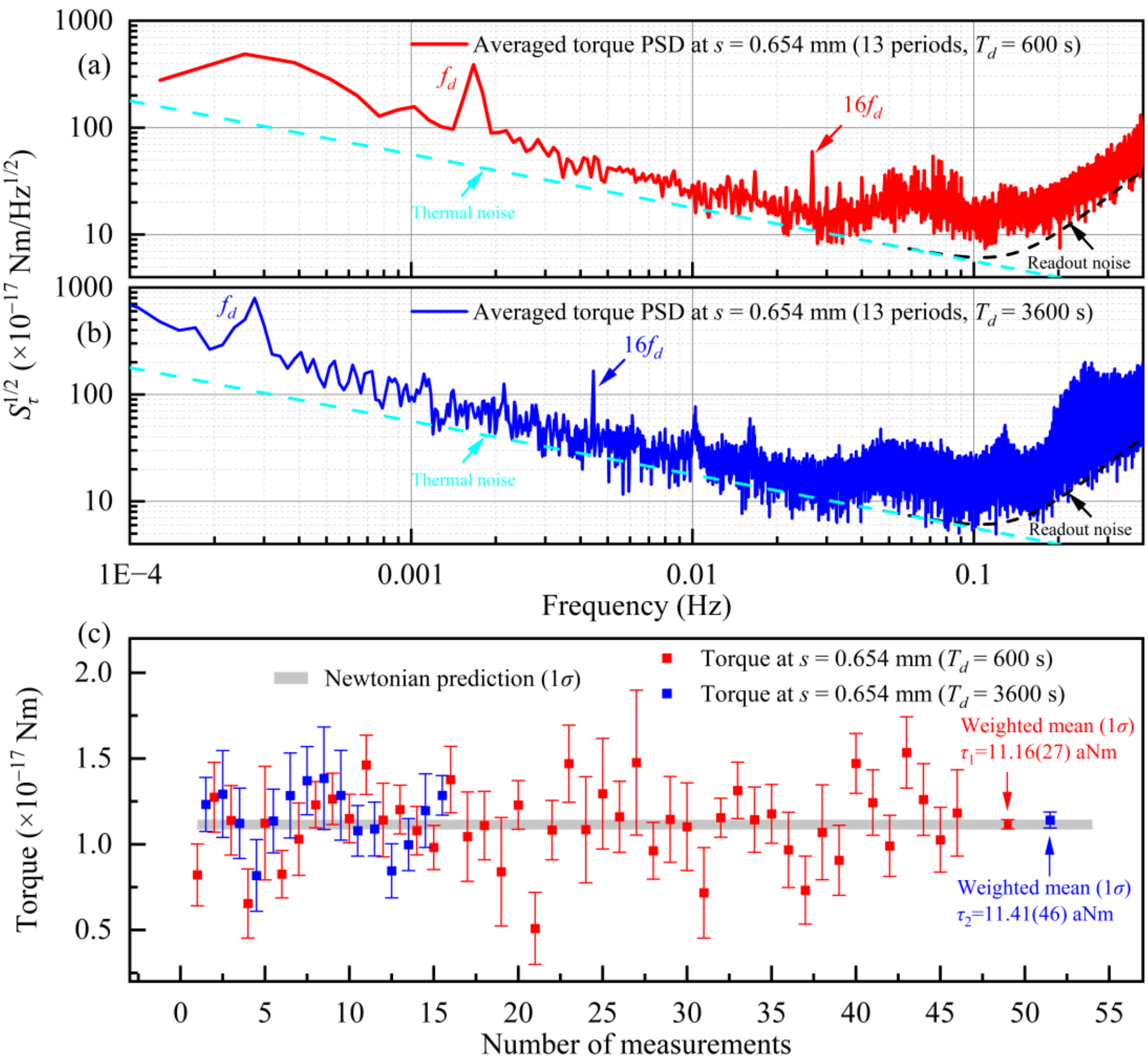

Supplementary Figure 4 Comparison of measured gravitational torques for the source mass disk rotating at two different periods (600 s vs 3600 s) at s = 0.654 mm. (a) and (b) Averaged torque power spectral densities (PSDs) of the torsion balance from multiple independent measurement segments (each containing 13 rotation periods) at s = 0.654 mm for rotation periods of 600 s and 3600 s, respectively. Distinct signals were observed at frequency $16f_d$ (26.667 mHz and 4.444 mHz, respectively). (c) Torque amplitudes at $16f_d$ frequency extracted from data segments using the time-domain orthogonal decomposition method. Each point represents the average torque over 13 rotation periods. At the signal frequency of 26.667 mHz, 46 measurements yielded a weighted mean τ_1 = 11.16(27) aNm, while at 4.444 mHz, 15 measurements yielded τ_2 = 11.41(46) aNm. The uncertainties represent 1σ statistical uncertainties only. The gray band indicates the theoretical Newtonian torque of 11.17(26) aNm at this separation distance. The elevated noise level at 4.444 mHz compared to 26.667 mHz primarily results from increased $1/f$ noise characteristics of the suspension fiber at lower frequencies. All measurements remain consistent within 1σ uncertainties, confirming the frequency independence of the gravitational measurement results.

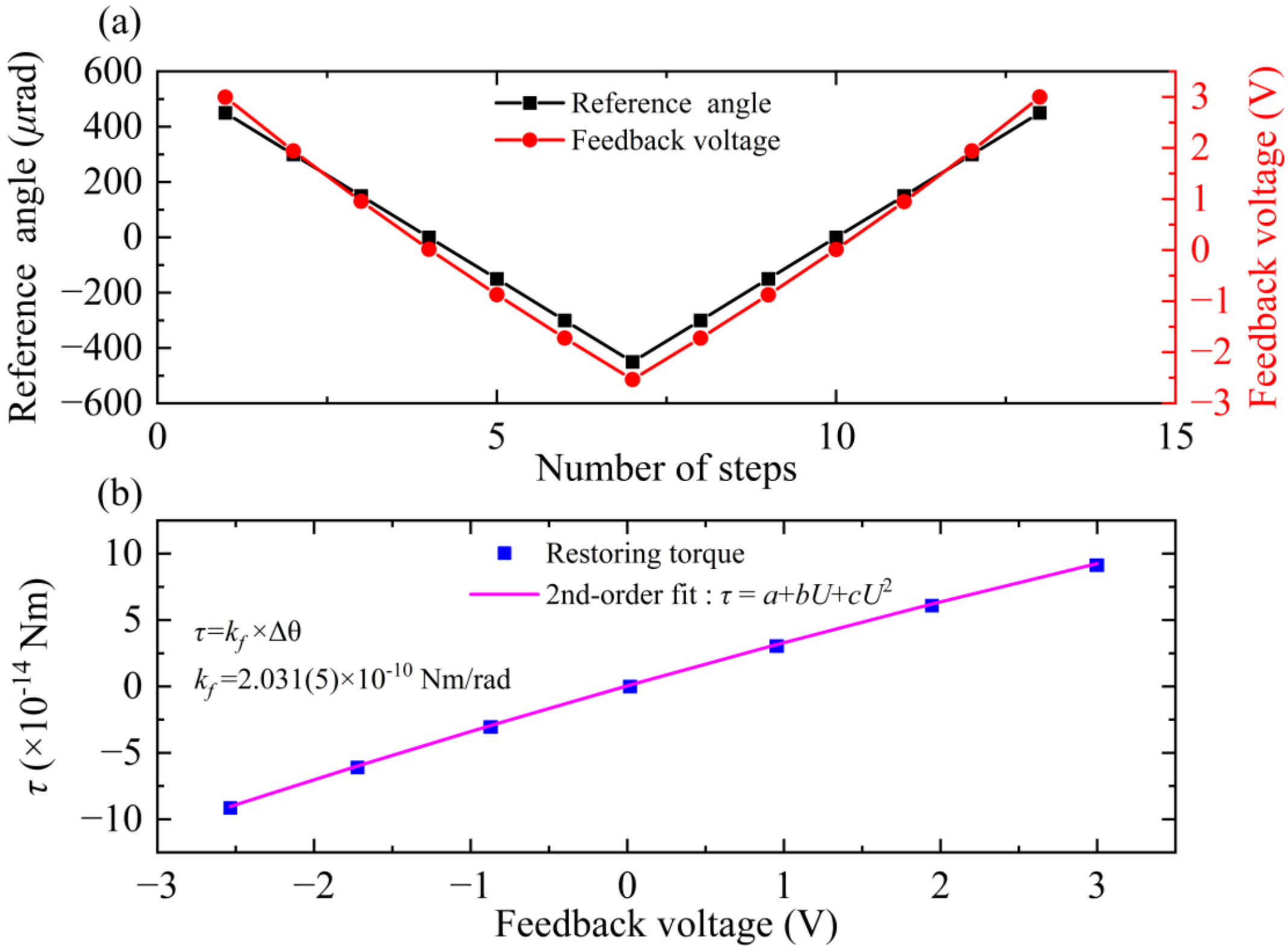

Supplementary Figure 5 Determination of the electrostatic control coefficient β of feedback voltage to torque. During the measurement, a large separation (≥6 mm) is maintained between the pendulum and the shielding membrane, allowing the residual electrostatic stiffness ke to be neglected. In this case, the open-loop torsion pendulum behaves as a harmonic oscillator in rotational motion. The open-loop stiffness k_f (primarily contributed by the suspension fiber) is determined through the natural torsional frequency ω0 and the pendulum's moment of inertia I, yielding $k_f = I\omega_0^2 = 2.031(5) \times 10^{-10}$ Nm/rad. By sweeping the reference angle in closed loop and recording the feedback voltage applied to the actuator, the ratio β is obtained. Note that β depends on the pendulum and capacitive actuator but is unaffected by separation from the shielding membrane. (a) One complete adjustment cycle of the torsion balance's reference angle, with adjustments within ±600 μrad in steps of approximately 150 μrad. (b) Relationship between restoring torque τ and feedback voltage U, where $\tau = k_f \times \Delta\theta$. The data points (blue squares) show restoring torque as a function of applied feedback voltage. A second-order polynomial $\tau = a + bU + cU^2$ is fitted to the data (solid line), yielding coefficients: $a = -5.6(3) \times 10^{-16}$ Nm, $b = 3.348(1) \times 10^{-14}$ Nm/V, and $c = -9.28(7) \times 10^{-16}$ Nm/V². From the fitting result, the calibrated electrostatic torque coefficient is derived as $\beta = \partial\tau/\partial U = b + 2cU$. Multiple calibrations yield $\beta = 3.34(5) \times 10^{-14} - 1.88(2) \times 10^{-15}\ U$. At the typical operating point ($U = 0$), $\beta_0 = 3.34(5) \times 10^{-14}$ Nm/V.

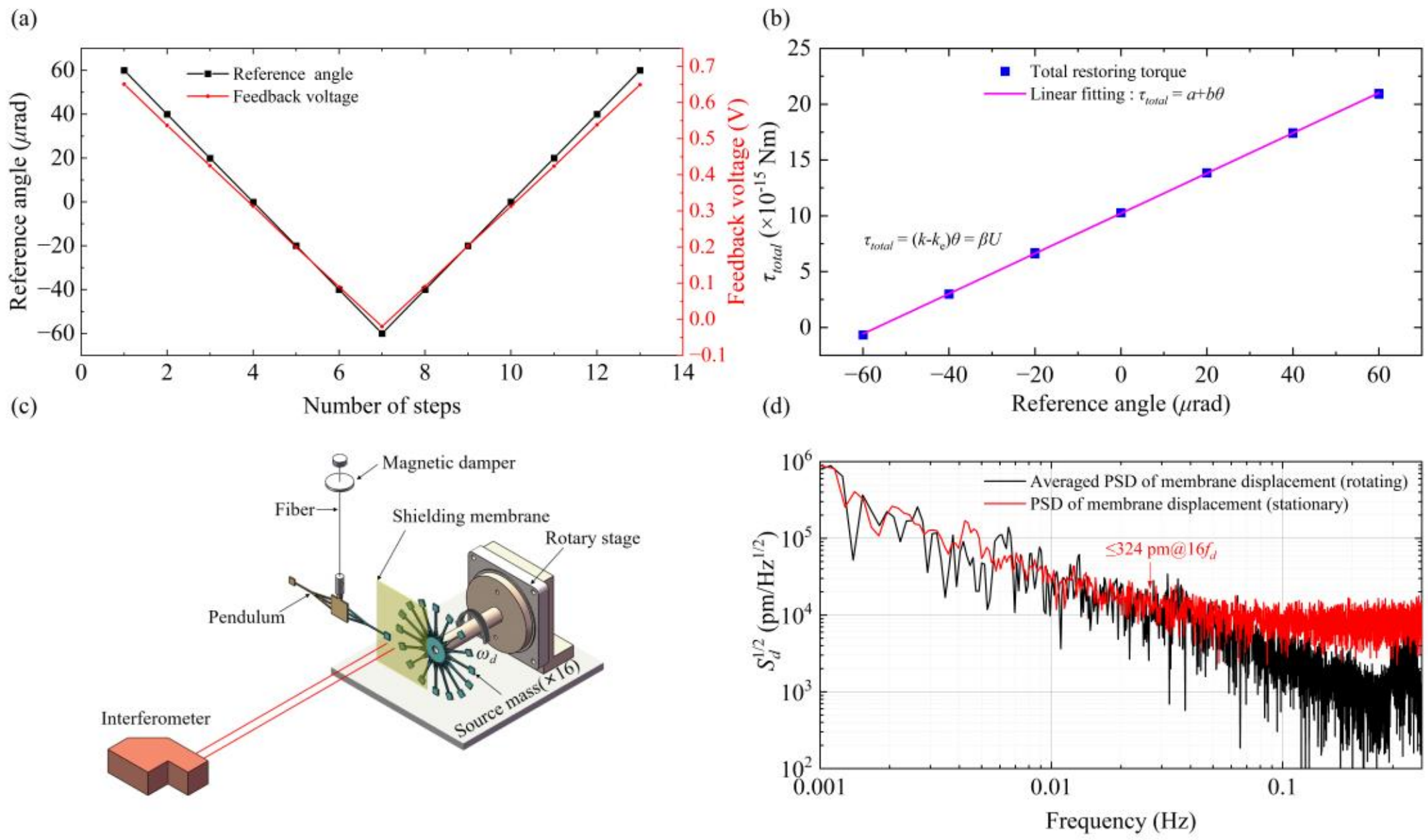

Supplementary Figure 6 Determination of the shielding membrane motion and electrostatic effect estimation between the membrane and pendulum. (a) Electrostatic stiffness determination. By systematically varying the reference angle of the torsion pendulum and recording the corresponding feedback voltage ΔU, we determined the electrostatic stiffness ke introduced by the shielding membrane to be 19.4(2.7) pNm/rad (averaged from multiple calibration measurements). This determination was based on the relationship $(k_f - k_e)\Delta\theta = \beta\Delta U$, where k_f represents the torsion fiber stiffness and β denotes the control torque coefficient (feedback voltage to torque), both parameters having been previously characterized in Supplementary Figure. 5. (b) Total torque gradient analysis. Linear fitting of the experimental data yielded a total torque gradient of $\partial\tau_{\rm total}/\partial\theta = 1.799(5) \times 10^{-10}$ Nm/rad. (c) Displacement measurement methodology. A schematic diagram illustrates the measurement technique for membrane translational displacement using an ultra-precision laser interferometer (HUE-XL, Harbin Institute of Technology). (d) Translational motion analysis. The power spectral densities (PSDs) of membrane translation displacement were measured with the turntable in stationary (black) and rotating (red, averaged from three independent 7,800 s measurements) conditions. At the critical frequency of $16f_d$ (= 26.667 mHz), no statistically significant differences were observed, confirming that turntable rotation does not introduce substantial translational motion to the membrane at the frequency of interest. Under the displacement noise threshold (from the PSDs) of less than 324 pm, the resulting parasitic electrostatic torque is estimated as $\delta\tau_e = k_e \cdot \delta s / L_{eq} \leq 0.27$ aNm (with k_e = 19.4(2.7) pNm/rad, $\delta s \leq 324$ pm, and L_{eq} = 23.694(3) mm).

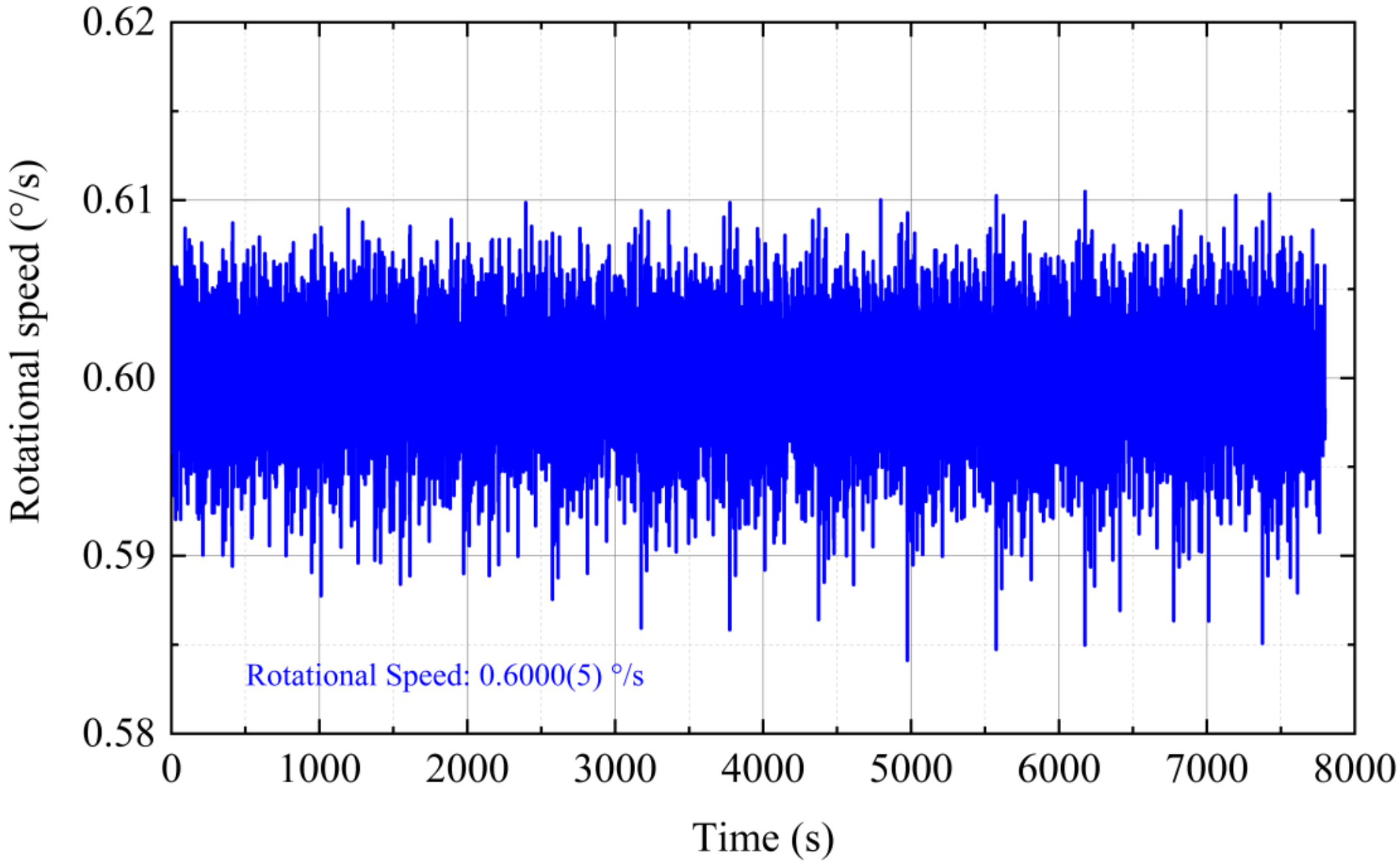

Supplementary Figure 7 Measurement of the rotational speed of the attractor using an angle encoder integrated in the rotary stage. The mean rotational speed is 0.6000(5) °/s, corresponding to a frequency of f_d = (1.6667 ± 0.0014) mHz, with a contribution to the torque uncertainty of 0.08 aNm.

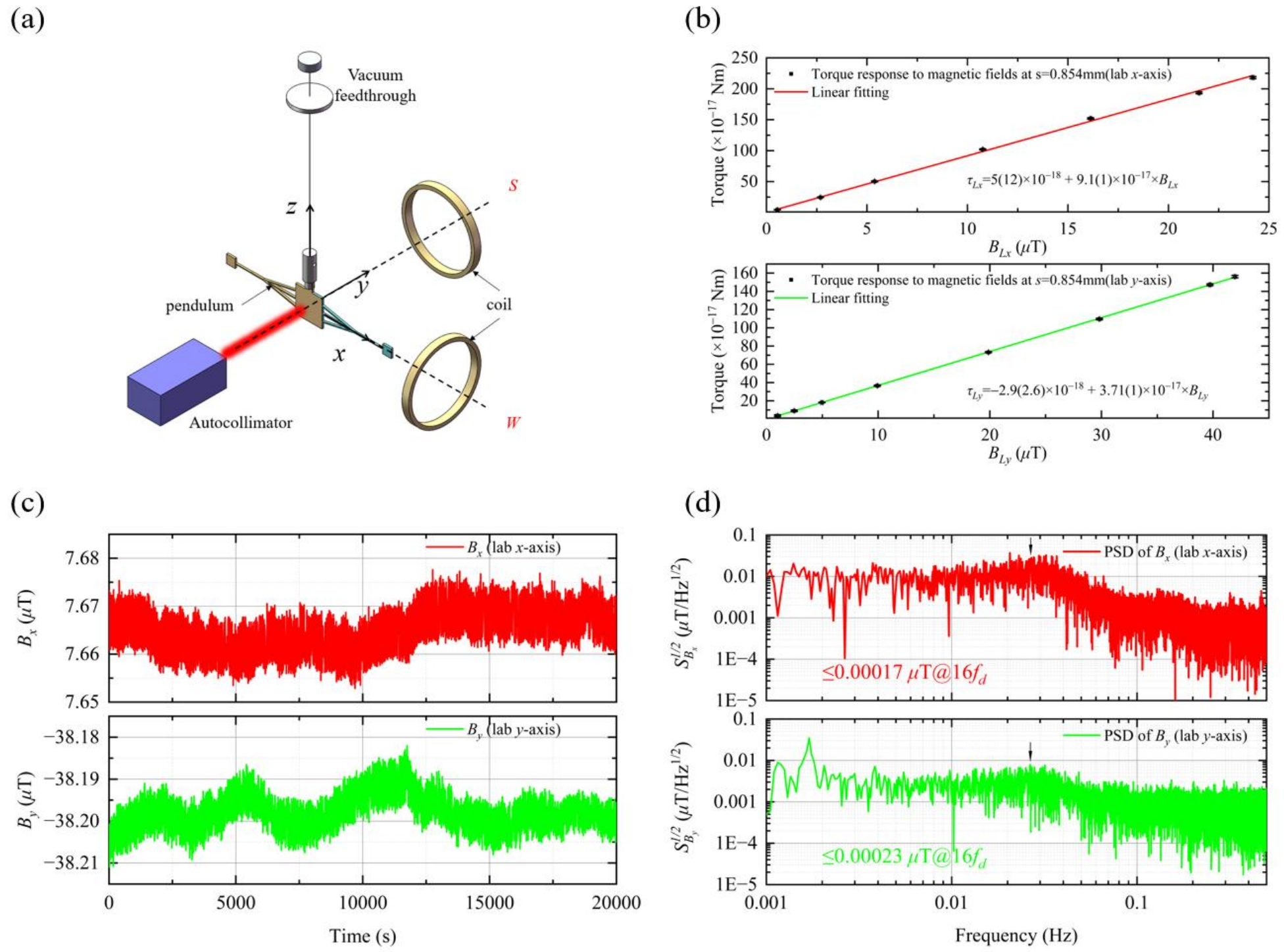

Supplementary Figure 8 Analysis of torsion balance response to alternating magnetic fields. (a) Experimental configuration. The torsion balance responds exclusively to vertical torque components, making horizontal magnetic field variations the primary consideration. To characterize the magnetic torque response, a pair of horizontally orthogonal coils was implemented to generate controlled alternating magnetic fields for systematic calibration. (b) Torque response characterization. The torsion balance's torque response to alternating magnetic fields was measured at a modulation frequency of 26.667 mHz ($16f_d$). The plot displays magnetic field amplitude (horizontal axis) versus extracted torque amplitude at the corresponding frequency (vertical axis). Linear regression analysis yielded sensitivity coefficients for the x- and y-axes: $\eta_x = \partial\tau/\partial B_x = 9.1(1) \times 10^{-17}$ Nm/μT and $\eta_y = \partial\tau/\partial B_y = 3.71(1) \times 10^{-17}$ Nm/μT. (c) Magnetic field monitoring. Magnetic field along laboratory x- and y-directions were continuously monitored using a Bartington Mag-13 magnetometer positioned adjacent to the torsion balance. The measurement duration was approximately 20,000 seconds, with the turntable operating in rotation mode throughout the data acquisition period. (d) Spectral analysis and torque fluctuation calculation. Power spectral density (PSD) analysis of the magnetic field data revealed fluctuations. Applying the same methodology used for gravitational signal analysis, the magnetic field fluctuation amplitudes at the $16f_d$ frequency were determined as $\delta B_x \leq 0.00017\ \mu$T and $\delta B_y \leq 0.00023\ \mu$T. Using the relationship $\delta\tau = \eta \cdot \delta B$, the corresponding torque fluctuations at $16f_d$ were calculated as $\delta\tau_x \leq 1.5 \times 10^{-20}$ Nm and $\delta\tau_y \leq 9 \times 10^{-21}$ Nm. The combined torque fluctuation from horizontal magnetic field variations is given by $\delta\tau_{B_\perp} = \sqrt{(\delta\tau_x)^2 + (\delta\tau_y)^2} \leq 0.02\ \mathrm{aNm}$, which falls within the total uncertainty budget of the magnetic effect.

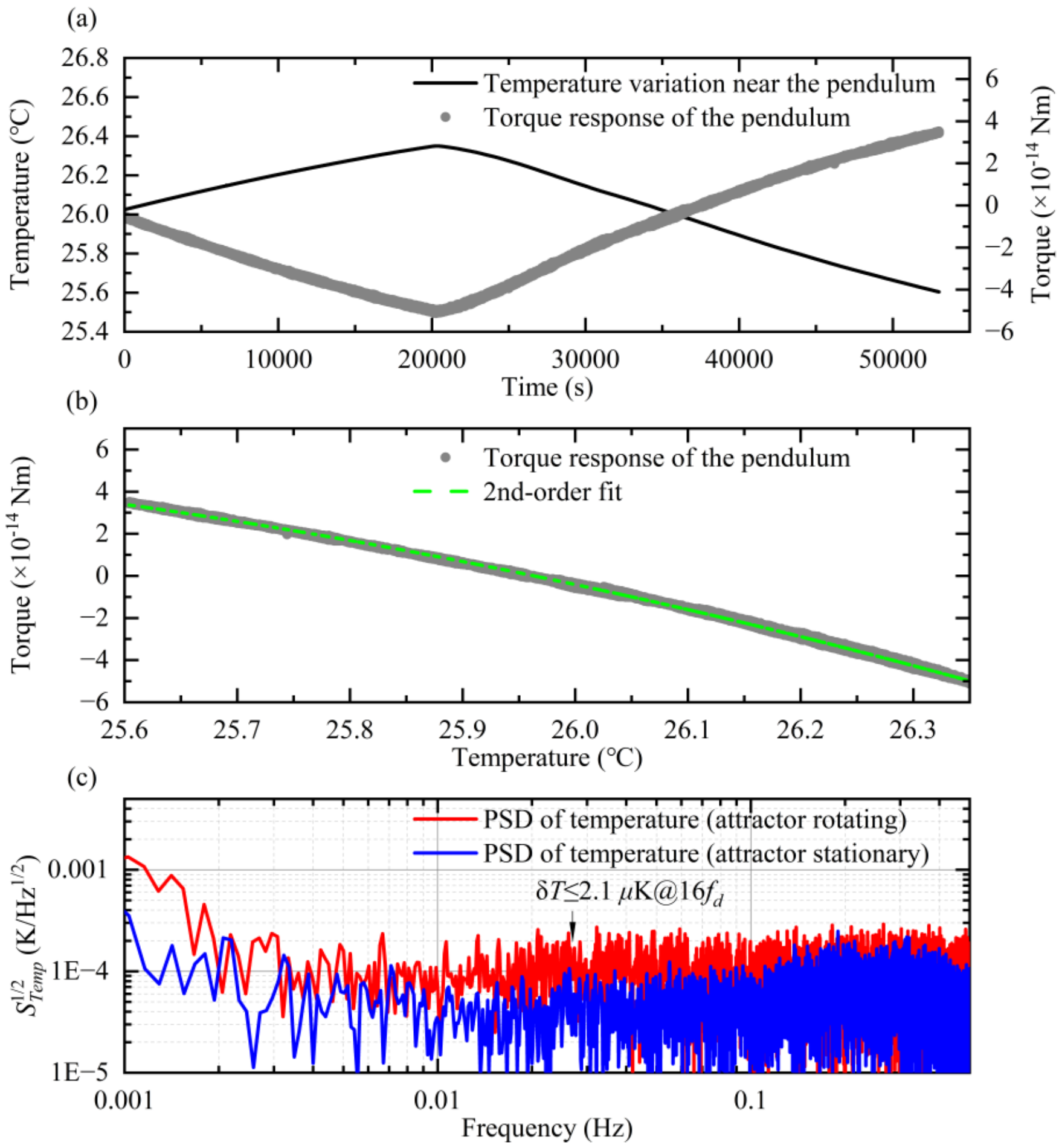

Supplementary Figure 9 Temperature effect evaluation through ambient temperature variation and pendulum torque response. (a) Time-domain characterization. The typical time-domain data illustrates the measured ambient temperature variation around the pendulum's location and the corresponding torque response, establishing a direct correlation between thermal fluctuations and mechanical response. (b) Temperature-torque relationship analysis. The relationship between torque and temperature is depicted (gray dots). A second-order polynomial fit (green dashed line) was applied to the experimental data according to the equation $\tau = a + bT + cT^2$, yielding coefficients: $a = -2.907(5) \times 10^{-11}$ Nm, $b = 2.350(4) \times 10^{-12}$ Nm/°C, and $c = -4.738(8) \times 10^{-14}$ Nm/(°C)². The temperature-to-torque coefficient is then derived as $k_T = \partial\tau/\partial T = b + 2cT$. At the typical experimental temperature of $T_0 = 26$ °C, this gives $k_T = (-114.2 \pm 5.7)$ fNm/°C. (c) Frequency-domain analysis and torque estimation. Power spectral densities (PSDs) of the measured temperature around the pendulum's location (red line) during a 7,800 s experimental run reveal a $16f_d$ variation of $\delta T \leq 2.1$ μK, which is extracted using the same method as the gravitational signal. This signal is superimposed on the background noise measured when the attractor is stationary (blue line). By combining the maximum temperature variation of 2.1 μK with the temperature coefficient k_T, the resulting torque at $16f_d$ is calculated as $\delta\tau_T = k_T \cdot \delta T \leq 0.24$ aNm.

Supplementary Note 1 – Fabrication process of test and source mass.

Silicon wafer etching is a mature semiconductor manufacturing process. Below is a brief introduction to the fabrication procedure used in this work, accompanied by a recommended reference book for further details [1].

In this experiment, 500-μm-thick silicon wafers were selected and subjected to advanced semiconductor lithography processing, including photolithography and subsequent deep silicon etching procedures. Initially, a uniform photoresist layer (typically positive or negative photoresist with a thickness of approximately 1–3 μm) was spin-coated onto the silicon wafer surface. Subsequently, the pre-designed micrometre-scale patterns were precisely transferred onto the photoresist layer using direct-write UV exposure systems. Following exposure, the development process removed either the exposed or unexposed photoresist regions, creating a precisely patterned photoresist mask that served as the etching mask for the subsequent steps.

High-precision etching was then accomplished using the Bosch deep silicon etching process (also known as pulsed plasma etching). This technique employs alternating SF_6 plasma etching steps and C_4F_8 passivation steps. In the SF_6 plasma environment, fluorine radicals react with silicon to form volatile SiF_4 gas, enabling isotropic silicon removal. Subsequently, in the C_4F_8 plasma environment, a fluorinated polymer passivation layer is deposited on the etched sidewalls, protecting them from further etching. Through this "etch–passivation" cyclic alternating process, deep structures up to several hundred micrometers can be achieved while maintaining excellent anisotropic etching characteristics.

Supplementary Figure 10–Supplementary Figure 12 show photographs taken during various stages of the fabrication process.

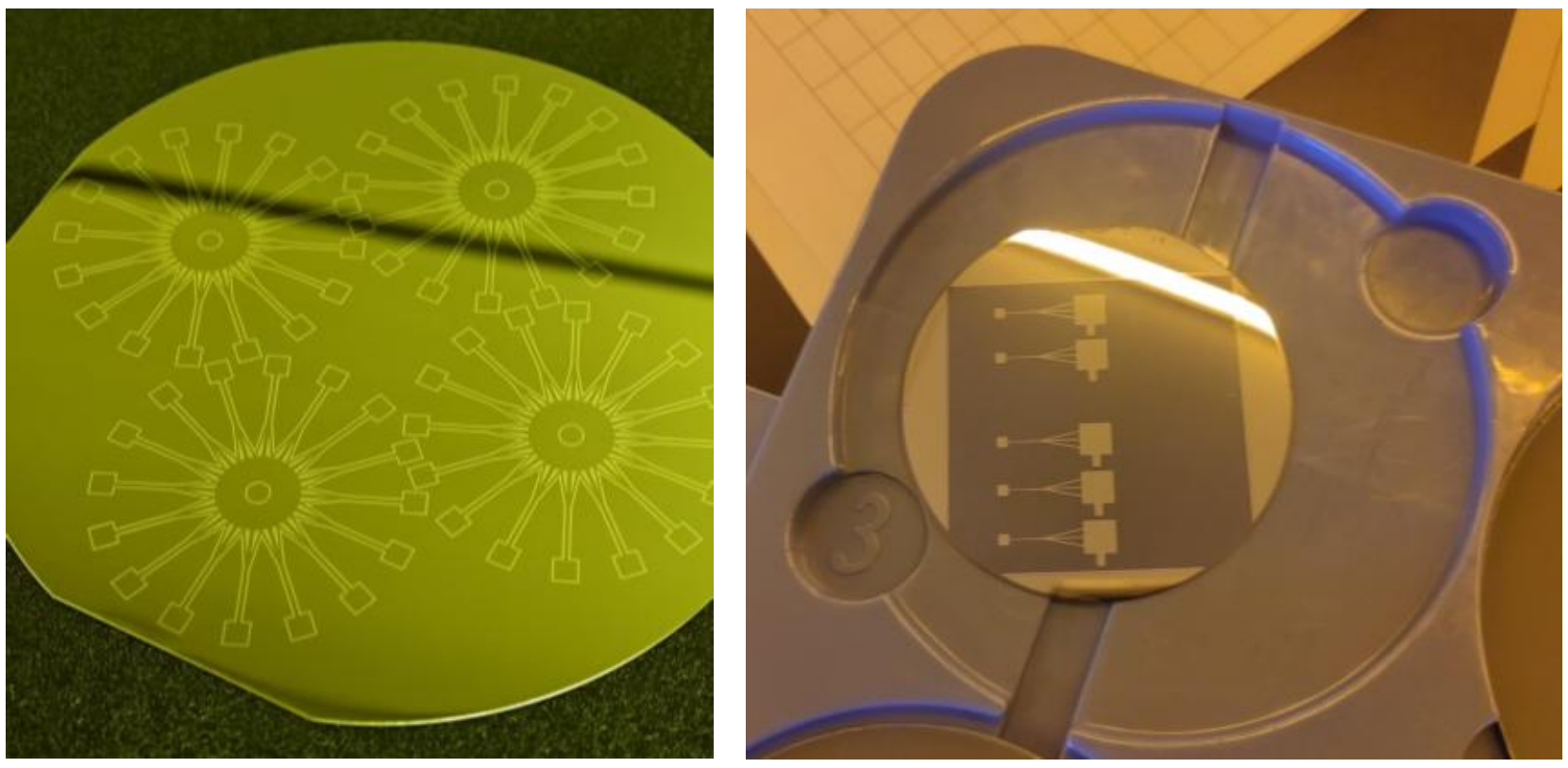

Supplementary Figure 10 Appearance photograph of lithography development during source mass processing.

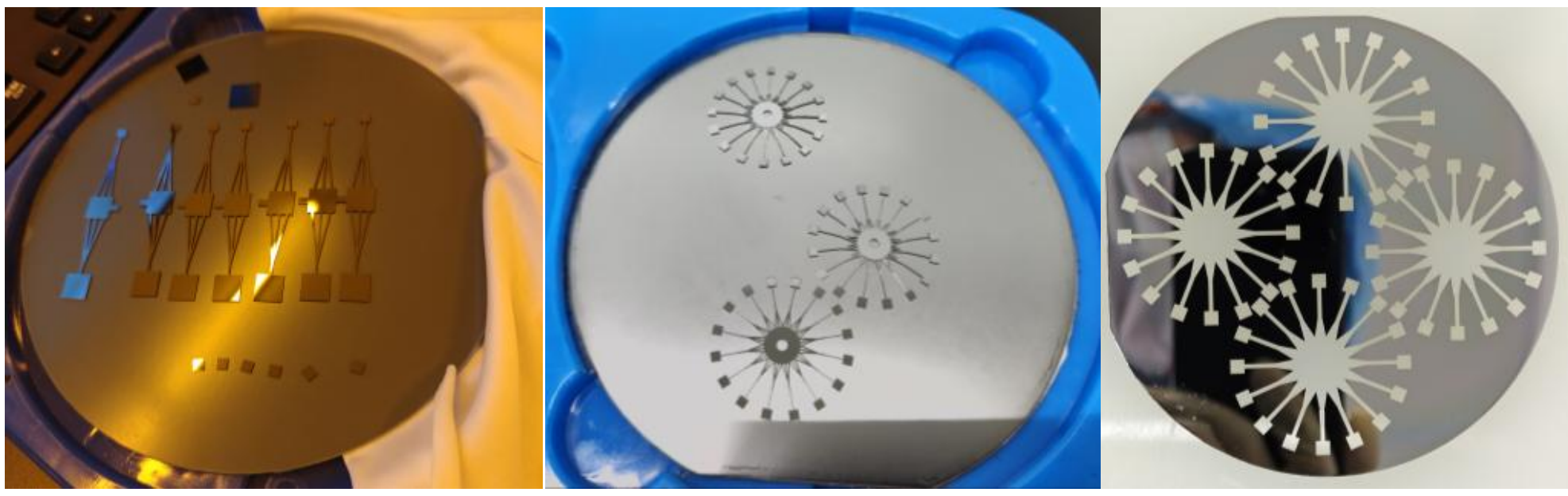

Supplementary Figure 11 Photograph of silicon wafer sample following deep silicon etching process.

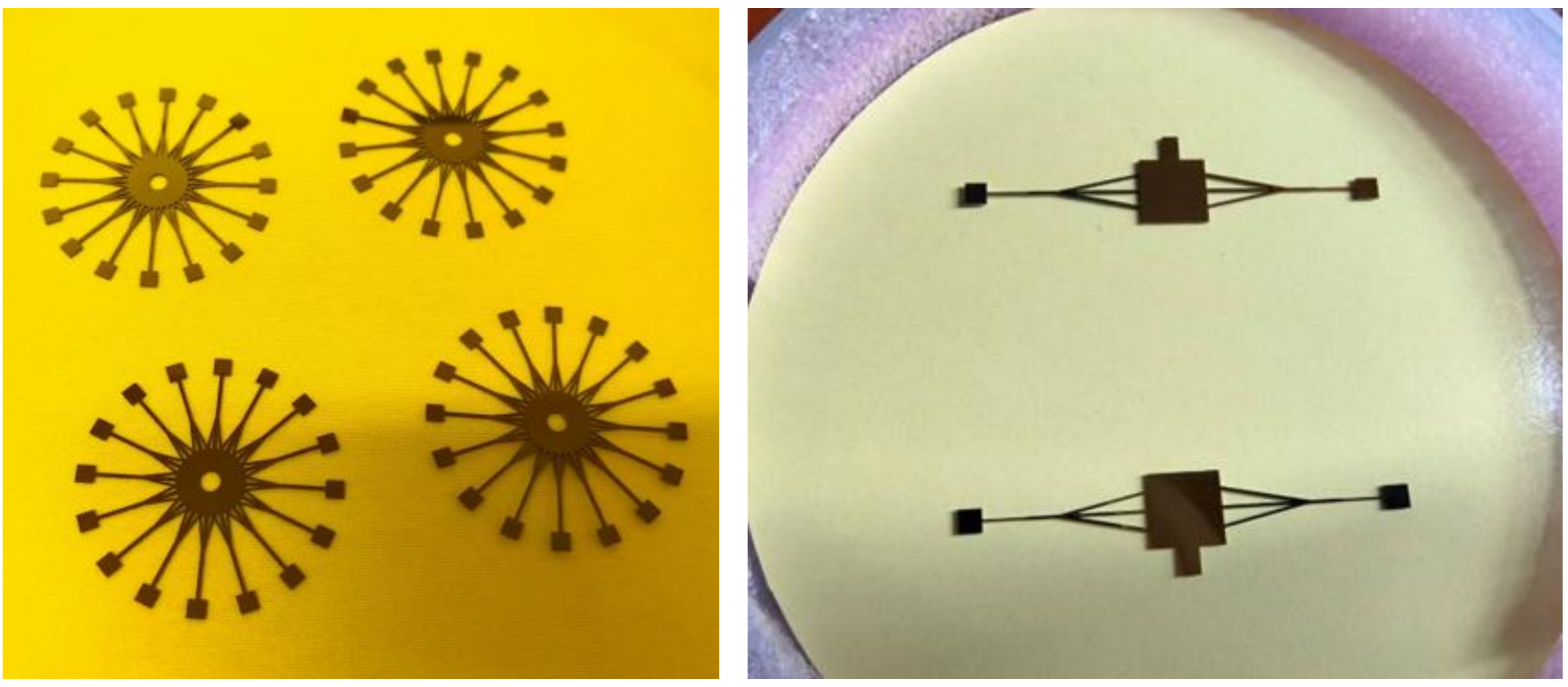

Supplementary Figure 12 Final experimental components after cleaning and dicing of processed silicon wafers.

Supplementary Note 2 – Alignment procedure.

The alignment procedure in this experiment follows the method established in our previous work [2,3]. The detailed procedure is described below.

2.1 Axiality adjustment of source mass disk and rotating shaft. Under the Video Measuring Machine, the axiality between the disk center and rotating shaft is adjusted to 40(7) μm. Subsequently, the disk is secured to the shaft using epoxy resin, with the perpendicularity between the disk face and shaft controlled by adjusting the thickness of the epoxy resin layer.

2.2 Measurement and correction of perpendicularity between disk face and rotating shaft. During rotary table rotation, an autocollimator is employed to measure the deviation angle between the disk face normal and the shaft axis. The resulting perpendicularity deviation between the disk face and rotating shaft is determined to be 1.86(13) mrad.

2.3 Shielding membrane attitude alignment. The shielding membrane and attracting mass are mounted on the same base plate. An autocollimator is used to simultaneously monitor the attitude of both the shielding membrane and the attracting mass disk face. The shielding membrane is then adjusted relative to the rotating shaft axis to achieve angular deviations of $\theta x \approx 887$ μrad and $\theta z \approx -630$ μrad, respectively.

2.4 Measurement of spacing between shielding membrane and source mass. Using a Video Measuring Machine, the spacing between the shielding membrane and the center of the attracting mass disk face is measured to be 0.268(3) mm.

2.5 Installation and calibration of alignment reference component. The alignment reference component is secured to the same base plate. The Video Measuring Machine is employed to measure the relative position of the alignment reference component with respect to the center area of the attracting mass disk face and the rotating shaft.

2.6 Alignment of source mass and torsion pendulum in vacuum chamber. After installing the entire apparatus in the vacuum chamber, the torsion pendulum is aligned relative to the alignment reference component by making contact between the reference component and the torsion pendulum suspension wire and pendulum itself, following the methodology from our

previous work [3] . The relative position of the torsion pendulum with respect to the reference component is thereby determined. Subsequently, the relative position between the torsion pendulum and source mass is calculated utilizing the pre-measured positional relationship between the reference component and the source mass disk.

2.7 Final positional parameters of source mass Rotating shaft. The relative position of the source mass rotating shaft with respect to the torsion pendulum center is determined as follows: horizontal direction (x-axis): 41.726(24) mm, vertical direction (z-axis): 0.002(20) mm. The face spacing is adjusted using a precision displacement stage (PI M112.1VG1) located at the bottom of the base plate, achieving an alignment precision superior to 20 μm.

Supplementary Note 3 – Evaluation of residual gas effects.

First, consider the squeeze-film damping force fluctuations between two closely spaced opposing parallel plates (corresponding to the torsion pendulum and the membrane, as shown in Fig. 1:(a) Schematic view of the experimental setup in the manuscript), whose power spectral expression is given below [4,5]:

$$S_F = 8k_B TPab\left[\left(\frac{2m}{\pi k_B T}\right)^{\frac{1}{2}} + \frac{\tau}{d}\right], (\mathrm{N}^2/\mathrm{Hz}) \tag{1}$$

Where k_B=1.38×10⁻²³ J/K is the Boltzmann constant, T=300 K is the ambient temperature. In experiment, the vacuum chamber is maintained by an ion pump with a vacuum pressure of $P \approx 10^{-5}$ Pa. a and b are the side lengths of the test/source mass (a=b= 2.6 mm), m is the mass of a single molecule (in a high-vacuum chamber, water molecules are typically the dominant species [6], here we take $m = 2.99\times10^{-26}$ kg), and d (=0.25mm) is the gap distance between the two plates. $\tau \approx 5.7\times10^{-6}$ s is the molecular escape time, obtained from Monte Carlo simulation under the current conditions. The calculated noise power spectral density of the squeeze-film damping force between the two plates is $S_F^{1/2'} \approx 2.4\times10^{-16}\mathrm{N}/\sqrt{\mathrm{Hz}}$. Substituting the effective lever arm of the torsion pendulum L_{eq} = 23.694(3) mm, the torque fluctuation noise is obtained as $S_{torque}^{1/2'} \approx 5.7\times10^{-18}\mathrm{Nm}/\sqrt{\mathrm{Hz}}$, which is far below the current thermal noise floor of the torsion balance ($\sim1\times10^{-16}\mathrm{Nm}/\sqrt{\mathrm{Hz}}$@26.7mHz , dominated by the structural dissipation of the torsion fiber) and can therefore be neglected.

Secondly, we consider the effect of the source mass disk rotation on gas molecule motion. This analysis is conducted from three aspects: (1) Due to the membrane and shielding box enclosing the source mass and turntable, the direct pathway for the source mass motion to affect the torsion balance through gas is eliminated. (2) Compared to the speed of gas molecules (500~600 m/s), the equivalent motion speed of the source mass (0.0002 m/s) has a negligible effect on the molecular collision rate. (3) The primary potential impact occurs when individual source masses alternately face the membrane, which can alter the collision frequency of

molecules with the membrane, leading to periodic pressure variations that affect membrane motion.

We employ the Monte Carlo method for molecular collision simulation. As illustrated in the figure below, particles are generated at random positions within a 12×12×0.25 mm³ rectangular prism, with initial velocities assigned according to the Boltzmann distribution. The particle count satisfies the ideal gas law *PV=NRT*, and particle trajectories are subsequently tracked. When particles collide with the membrane or source mass, they undergo diffuse reflection (shown as red and green particles), with their impulse, position, and time recorded. The simulation continues until particles reach the rectangular prism boundary (shown as yellow particles), at which point they are considered to have escaped.

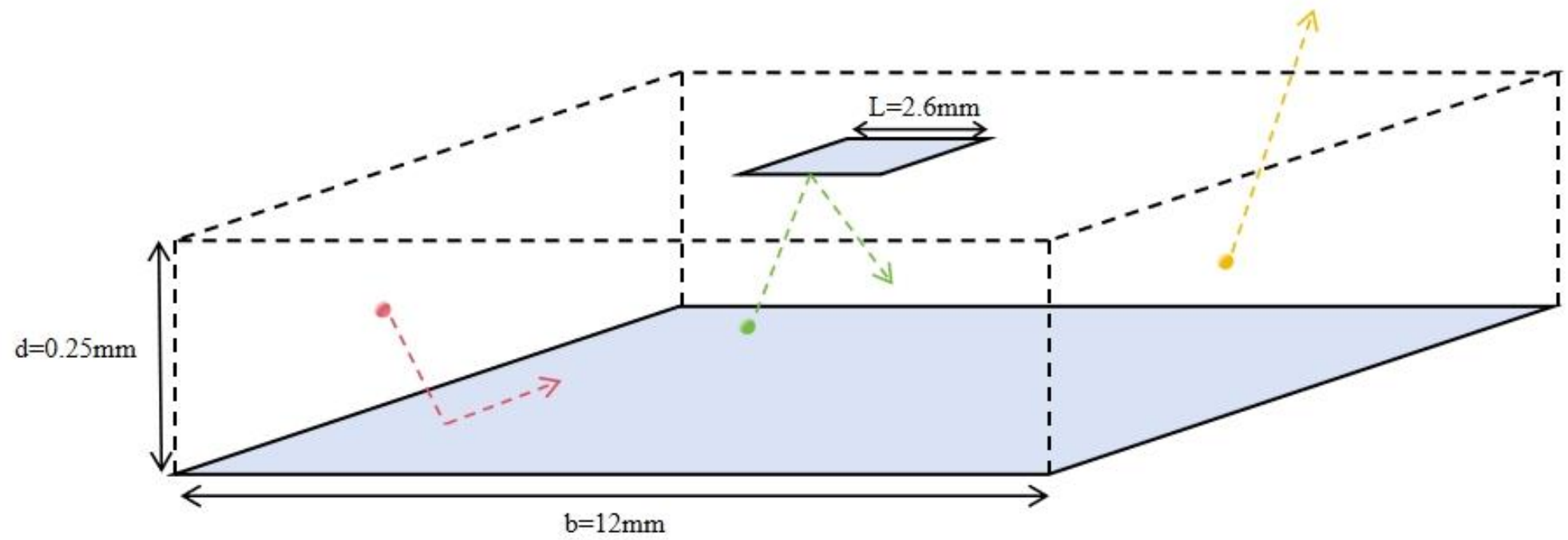

Supplementary Figure 13 Monte Carlo simulation of molecular collision process between two plates.

Through the simulation, we can provide both force fluctuation noise and derive pressure variations. Simultaneously, we can collect molecular collision counts to estimate the force acting on the shielding membrane surface during the simulation. For the central region of the shielding membrane with area A = 2.6×2.6 mm², the simulation yields molecular collision counts of $n_1 = 2.1\times10^6$ without the source mass and $n_2 = 19\times10^6$ with the source mass present. As pressure is proportional to molecular collision counts, we conducted a conservative maximum estimation. During the source mass disk rotation, we assumed that the pressure difference on the membrane surface, caused by residual gas molecules in the vacuum chamber ($P \approx 1 \times 10^{-5}$Pa), reaches a value of $\Delta F = \frac{n_2-n_1}{n_1}PA \approx 5.44 \times 10^{-10}$N.

Finally, once the pressure difference on the membrane surface caused by the rotating source mass disk is determined, SolidWorks simulation yields deformation of the membrane or frame

system, the maximum is approximately 2.6×10^{-4} nm. This value is completely negligible compared to the membrane vibration size measured by using an interferometer (<0.324 nm) as shown in **Supplementary Figure 6**.

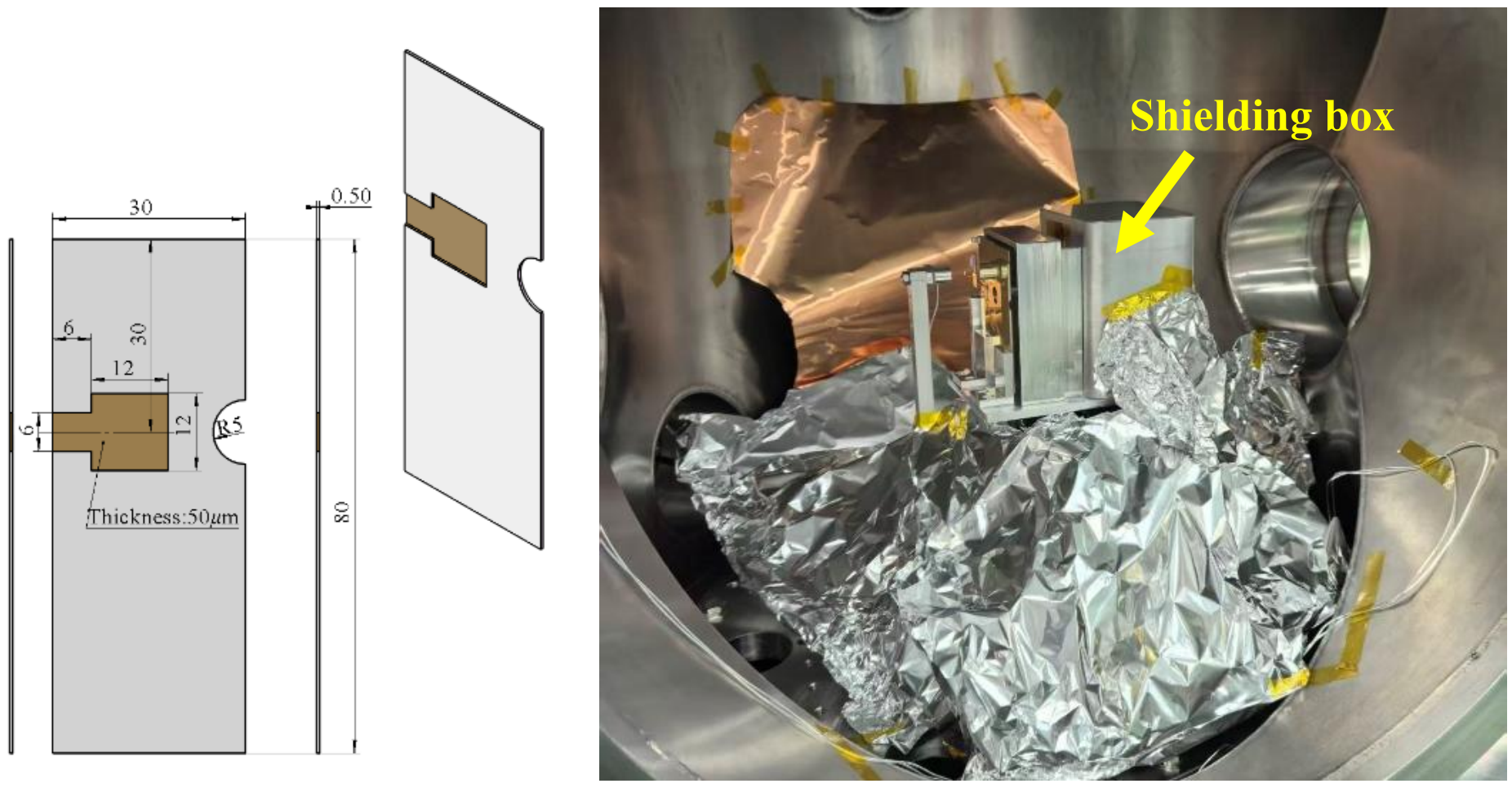

Supplementary Figure 14 (Left) Design drawing of the shielding membrane and frame, which consists of a 0.5 mm thick silicon wafer overall, with a thickness of 0.05 mm in the area directly opposite between the test and source masses (approximately 12×12 mm^2). (Right) Shielding box enclosing the motor and source mass to reduce disturbances such as electrostatic and air turbulence.

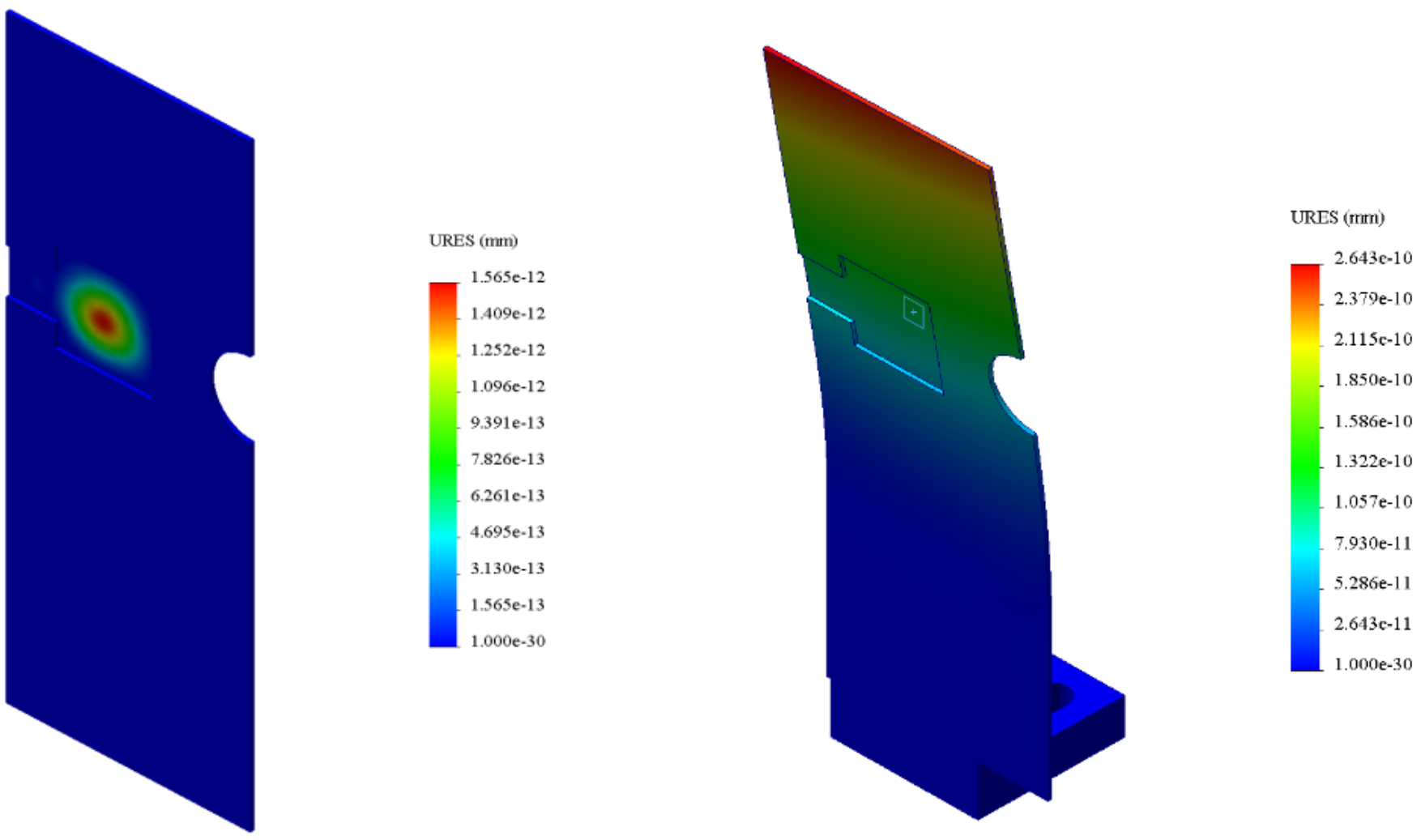

Supplementary Figure 15 Force-induced deformation of the membrane and frame simulated using SolidWorks software. (Left) Frame fixed, with deformation only simulated for the membrane region. (Right) Bottom of the frame fixed, with deformation simulated for both the membrane region and the entire frame, the maximum deformation is approximately 2.6×10^{-4} nm, it can be completely disregarded.

Supplementary Note 4 – Signal extraction method.

In the closed-loop system, the feedback voltage $U(t)$ directly reflects the external torque (also including the thermal noise) acting on the pendulum. We extract the signal amplitude at $16\omega_d$ using a time-domain orthogonal decomposition method, which coherently accumulates the signal over N full rotation periods while averaging down random noise.

4.1 Data preprocessing methods.

Each data segment has a duration of T=7800 s, containing N=13 complete rotation periods of the attractor, with each period T_d =600 s. The sampling rate is 1 Hz, giving 600 data points per period. Before spectral analysis, the low-frequency drift in $U(t)$ is removed using a third-order polynomial fitting as

$$U_d(t) = U(t) - (a_0 + a_1 t + a_2 t^2 + a_3 t^3) \qquad (2)$$

where the coefficients a_0,a_1,a_2,a_3 are obtained by least-squares fitting over the entire data segment.

4.2 Extraction of the target frequency component.

The target signal frequency is the 16th harmonic of the attractor rotation frequency, i.e.,ω_s =$16\omega_d$ =$2\pi\times$26.667mHz. For each rotation period, we extract the cosine and sine components of the feedback voltage at ω_s by orthogonal decomposition. For the i-th rotation period (i=1,2,…,N):

$$U_{\cos,i} = \frac{2}{T_d}\int_0^{T_d} U_d(t)\cos(\omega_s t)dt \quad (3) \qquad (4)$$

$$U_{\sin,i} = \frac{2}{T_d}\int_0^{T_d} U_d(t)\sin(\omega_s t)dt \qquad (5)$$

In the discrete time domain, these integrals become sums:

$$U_{\cos,i} = \frac{2}{M}\sum_{j=0}^{M-1} U_d[j]\cos(\omega_s t_j) \qquad (6)$$

$$U_{\sin,i} = \frac{2}{M}\sum_{j=0}^{M-1} U_d[j]\sin(\omega_s t_j) \qquad (7)$$

Where M=600 is the number of samples per rotation period, $t_j = j\cdot T_s$, and T_s =1s is the sampling interval.

4.3 Outlier rejection and weighted average within one data segment.

For the 13 periods in one data segment, we calculate the means and standard deviations of $U_{\cos,i}$ and $U_{\sin,i}$:

$$\bar{U}_{\cos} = \frac{1}{13}\sum_{i=1}^{13} U_{\cos,i}, \quad \sigma_{U_{\cos}} = \sqrt{\frac{1}{12}\sum_{i=1}^{13}(U_{\cos,i} - \bar{U}_{\cos})^2} \tag{8}$$

$$\bar{U}_{\sin} = \frac{1}{13}\sum_{i=1}^{13} U_{\sin,i}, \quad \sigma_{U_{\sin}} = \sqrt{\frac{1}{12}\sum_{i=1}^{13}(U_{\sin,i} - \bar{U}_{\sin})^2} \tag{9}$$

A 3σ criterion is applied to reject outliers: if $|U_{\cos,i} - \bar{U}_{\cos}| > 3\sigma_{U_{\cos}}$ or $|U_{\sin,i} - \bar{U}_{\sin}| > 3\sigma_{U_{\sin}}$ for a given period, that period is marked as an outlier and excluded. After outlier rejection, the remaining number of valid periods is N'. The mean values and their standard errors are recalculated:

$$\bar{U}_{\cos} = \frac{1}{N'}\sum_{i=1}^{N'} U_{\cos,i}, \quad \delta U_{\cos} = \sqrt{\frac{1}{N'(N'-1)}\sum_{i=1}^{N'}(U_{\cos,i} - \bar{U}_{\cos})^2} \tag{10}$$

$$\bar{U}_{\sin} = \frac{1}{N'}\sum_{i=1}^{N'} U_{\sin,i}, \quad \delta U_{\sin} = \sqrt{\frac{1}{N'(N'-1)}\sum_{i=1}^{N'}(U_{\sin,i} - \bar{U}_{\sin})^2} \tag{11}$$

Thus, for this data segment (13 rotation periods), the feedback voltage components and their statistical errors are $(\bar{U}_{\cos}, \delta U_{\cos})$ and $(\bar{U}_{\sin}, \delta U_{\sin})$.

4.4 Voltage dependence of the electrostatic control coefficient.

In our experiment, the electrostatic control coefficient β depends linearly on the feedback voltage U due to the relatively smaller area of the electrode plates, is shown in **Supplementary Figure 5**:

$$\beta(U) = 3.34(5)\times 10^{-14} - 1.88(2)\times 10^{-15} U \tag{12}$$

At the typical operating point U=0, this gives β_0 =33.4(5) fNm/V. In the time-domain orthogonal decomposition method, the cosine and sine components for each rotation period (T_d =600 s) are extracted by integrating over the full period, shown in (section 1.2), the β value used in the transfer function thus corresponds to the mean control voltage over that period. This effectively suppresses the influence of the voltage dependence of $\beta(U)$ on the transfer function

calibration, given that the typical slow drift of the voltage is~0.2 V/h. In frequency-domain analysis, by contrast, each bin corresponds to the Fourier transform of the voltage data cut with length of 7800 s, where the voltage dependence of β would introduce additional errors. Hence the time-domain method is better suited for our experiment.

4.5 Conversion to torque and error propagation.

The voltage components are converted to torque components using the transfer function of the closed-loop torsion pendulum. The magnitude of the transfer function $|H_U(\omega_s)|$ is given by Eq. (2) in the main text:

$$H_U(\omega)=\frac{U(\omega)}{\tau(\omega)}=\frac{U(\omega)\theta(\omega)}{\theta(\omega)\tau(\omega)}=\frac{(k_p+\frac{k_i}{i\omega}+i\omega k_d)e^{-i\omega\Delta t}}{\left(k_f-k_e-I\omega^2\right)+i\frac{k_f}{Q}+\beta(k_p+\frac{k_i}{i\omega}+i\omega k_d)e^{-i\omega\Delta t}} \quad (13)$$

where:

I=12.61(1)×10^{-9} kg·m² is the moment of inertia of the pendulum,

k_f=203.1(5) pNm/rad is the torsional spring constant of the suspension fiber,

Q=1786(20) is the quality factor,

k_e=19.4(2.7) pNm/rad (at s=0.654 mm) is the electrostatic stiffness,

$\beta(U)$=3.34(5)×10^{-14}−1.88(2)×10^{-15}U (Nm/V) is the electrostatic control coefficient, which depends linearly on the feedback voltage U. At the typical operating point U = 0, this gives β_0=33.4(5) fNm/V.

k_p =0.02 V/μrad, k_i =0.002 V/(μrad·s), and k_d =0.1 V/(μrad·s^{-1}) are the proportional, integral, and derivative gains of the PID controller, respectively,

Δt=1s is the sampling interval delay.

Their values and calibrations are detailed in the Methods section of the main text.

The cosine and sine components and the uncertainties of the torque are:

$$\tau_{\cos}=\frac{\bar{U}_{\cos}}{|H_U(\omega_s)|},\quad \tau_{\sin}=\frac{\bar{U}_{\sin}}{|H_U(\omega_s)|} \quad (14)$$

$$\delta\tau_{\cos}=\frac{\delta U_{\cos}}{|H_U(\omega_s)|},\quad \delta\tau_{\sin}=\frac{\delta U_{\sin}}{|H_U(\omega_s)|} \quad (15)$$

For this data segment, the torque amplitude is:

$$\tau_{\text{seg}} = \sqrt{\tau_{\cos}^2 + \tau_{\sin}^2} \tag{16}$$

with uncertainty:

$$\delta\tau_{\text{seg}} = \sqrt{\left(\frac{\tau_{\cos}}{\tau_{\text{seg}}} \cdot \delta\tau_{\cos}\right)^2 + \left(\frac{\tau_{\sin}}{\tau_{\text{seg}}} \cdot \delta\tau_{\sin}\right)^2} \tag{17}$$

4.6 Weighted average across multiple data segments.

For multiple measurements at the same separation, suppose there are m data segments (each containing 13 rotation periods, corresponding to 7,800 s of data). For the k-th data segment, the extracted torque amplitude and its statistical uncertainty are (τ_k ,$\delta\tau_k$), which are combined using a weighted average:

$$\overline{\tau} = \frac{\sum_{k=1}^{m} \tau_k / (\delta\tau_k)^2}{\sum_{k=1}^{m} 1/(\delta\tau_k)^2}, \qquad \delta\overline{\tau}_{\text{stat}} = \sqrt{\frac{1}{\sum_{k=1}^{m} 1/(\delta\tau_k)^2}} \tag{18}$$

4.7 Example of statistical analysis of the measured data.

To confirm the presence of a genuine signal at $16\omega_d$, we perform a statistical analysis of the torque amplitudes obtained from multiple independent measurements. At separation s=0.654 mm, a total of m=46 independent measurements were performed (each corresponding to 13 rotation periods, i.e., 7,800 s of data). The weighted average of the 46 torque amplitudes τ_k is:

$$\overline{\tau} = 11.16 \text{ aNm}, \quad \delta\overline{\tau} = 0.27 \text{ aNm} \tag{19}$$

Supplementary Figure 1 shows the 46 individual torque measurements, along with the weighted mean and its uncertainty.

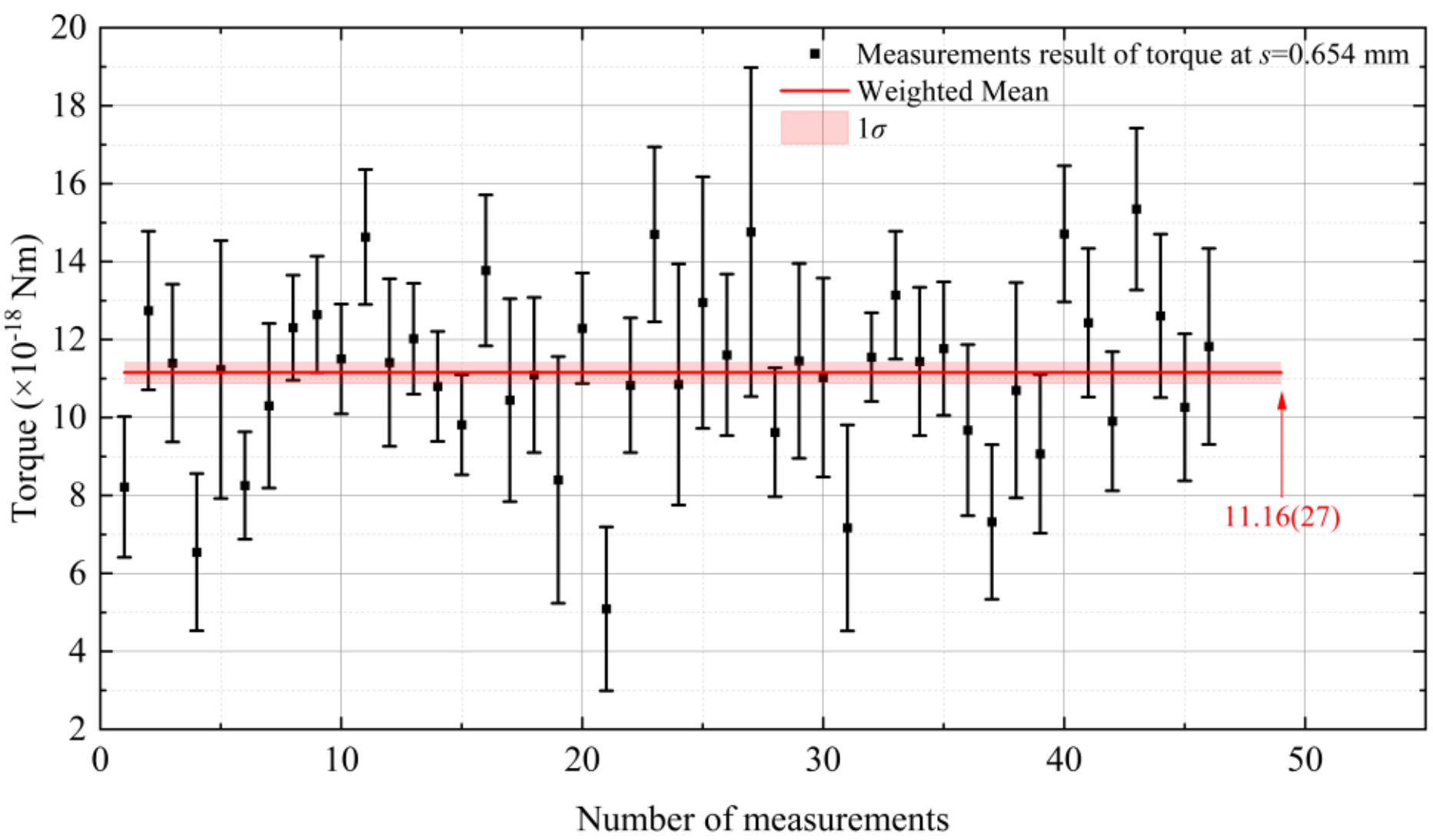

Supplementary Figure 16. Individual torque measurements from 46 experimental runs conducted at s = 0.654 mm. Each data point represents the extracted $16\omega_d$ torque amplitude derived from a single measurement segment lasting 7,800 s (equivalent to 13 rotation periods). The solid red line indicates the weighted mean value τ = 11.16 aNm, while the shaded band represents the 1σ standard error $\delta\tau$ = 0.27 aNm.

4.8 Effects of systematic uncertainties on the voltage U (ω) and transfer function H_U(ω).

The control voltage is generated by a PXI Multifunction I/O Module (National Instruments PXI-6289) featuring a 16-bit DAC resolution. Its output voltage was characterized using a high-precision digital voltmeter (Keysight 3458A). Since this experiment requires only the precise determination of the variation in output voltage, rather than the absolute voltage level, this offset is not of primary concern. In this experiment, the modulation signal amplitude corresponding to the gravitational torque (11.16 aNm) at the target frequency (~26.667 mHz) is approximately 0.33 mV, which was calibrated using the same digital voltmeter with an accuracy better than 3.3 µV. Consequently, this introduces a relative error of $\delta U(\omega_s)/U(\omega_s) \approx$ 1%, corresponding to a systematic uncertainty of approximately 0.11 aNm in the measured torque at a separation of 0.654 mm.

The systematic uncertainty of the transfer function $H_U(\omega_s)$ primarily is arised from the uncertainties in the electrostatic control coefficient β and the electrostatic stiffness k_e, which are discussed in **Supplementary Figure 5** and **6**. The relative uncertainty is estimated to be $\delta H_U(\omega_s)/H_U(\omega_s) \approx$ 2 %, corresponding to a systematic uncertainty of approximately 0.22 aNm in the measured torque.

Combining these two independent systematic uncertainties in quadrature gives:

$$\delta\tau = \sqrt{\left(\frac{1}{|H_U(\omega_s)|}\cdot\delta U\right)^2 + \left(\frac{U}{|H_U(\omega_s)|^2}\cdot\delta|H_U(\omega_s)|\right)^2} \tag{20}$$

Substituting $\delta U(\omega_s)/U(\omega_s) \approx 1\%$ and $\delta H_U(\omega_s)/H_U(\omega_s) \approx 2\ \%$ into Eq.(20), the combined systematic uncertainty from these two sources is estimated to be approximately 0.25 aNm. All systematic uncertainties are summarized in Table 1 of the main text.

Supplementary Note 5 – Sensitivity estimation.

In the Outlook section of the main text, we mention that shortening the separation from the current 654 μm to ~ 15 μm could yield a 10^5 improvement in signal-to-noise ratio. This estimation is based on the detection of **non-Newtonian Yukawa forces**, considering the combined effects of reduced separation and improved suspension fiber. The detailed derivation is provided below.

5.1 Signal torque estimation (non-Newtonian Yukawa deviations).

According to the reference [7], the Yukawa torque between two parallel plates can be written as:

$$\tau_Y = 2\pi\alpha G\rho_1\rho_2\lambda^2 A\left(1-e^{-t_1/\lambda}\right)\left(1-e^{-t_2/\lambda}\right)e^{-s/\lambda}L \tag{21}$$

Where s is the surface separation, λ is the Yukawa range, A is the area, t_1, t_2 are the thicknesses of the two plates, and L is the lever arm.

Taking $\alpha = 1$, $\rho = 2330$ kg/m^3 (silicon wafer), silicon wafer dimensions of 2.6 × 2.6 × 0.5 mm^3, lever arm $L = 23.7$ mm, and Yukawa range $\lambda = 90$ μm, the signal enhancement is approximately **1212 times**, as shown in Table below.

Surface separation (μm)	λ (μm)	Non-Newtonian torque (Nm)
654	**90**	2.05×10^{-21}
15	**90**	2.48×10^{-18}

5.2. Noise estimation.

The thermal noise torque of the suspension fiber scales as [8]:

$$S_\tau^{1/2}(f) = \sqrt{\frac{4k_BTk}{2\pi Qf}} \tag{22}$$

where the torsional stiffness is $k = \frac{\pi R^4 S}{2l}$, with R is the fiber radius, S is the shear modulus, and $l = 0.763$ m is the fiber length. Replacing the 10 μm tungsten fiber (Q~1800, $S = 161$ GPa)

with a 3 μm fused silica fiber (Q~25000, S = 29.47 GPa) under the same integration time, the thermal noise is reduced by approximately **97 times**, as shown in Table below.

Fiber	Diameter	Q	Torsional stiffness (Nm/rad)	Thermal noise (Nm/Hz$^{1/2}$) @26.667mHz
Tungsten	10 μm	1800	2.07×10^{-10}	1.07×10^{-16}
Fused silica	3 μm	25000	3.07×10^{-13}	1.10×10^{-18}

5.3. Combined SNR improvement.

$$\frac{SNR_{15\mu\mathrm{m}}}{SNR_{654\mu\mathrm{m}}} = \frac{\tau_{Y,15\mu\mathrm{m}}}{\tau_{Y,654\mu\mathrm{m}}} \cdot \frac{\tau_{\mathrm{noise},W}}{\tau_{\mathrm{noise},Q}} \approx 1212 \times 97 \approx 1.17 \times 10^5 \tag{23}$$

This confirms the statement of ***"a 10^5 improvement in signal-to-noise ratio can be obtained."***

5.4. Sensitivity estimation for measuring the Newtonian gravitational force between smaller masses.

Proposed parameters:

Material	Density (kg/m^3)	Dimensions (mm^3)	Mass (μg)	Separation (μm)	Lever arm (mm)	Newtonian gravitational torque (Nm)
Silicon	2330	2.6×2.6×0.5	**7875.4**	654	23.7	2.69×10^{-17}
Silicon	2330	0.3×0.3×0.1	**21.0**	15	23.7	1.70×10^{-20}

By reducing the silicon wafer dimensions from 2.6×2.6×0.5mm^3 to 0.3×0.3×0.1mm^3, the mass decreases from 7.8 mg to 21.0 μg, which is below the Planck mas m_P ≈22 μg. The surface separation is reduced from 654 μm to 15 μm. Under this configuration, the Newtonian gravitational torque is calculated to be approximately 1.7×10^{-20} Nm using numerical integration. Using a 3μm-diameter fused silica fiber (Q=25000, shear modulus S=29.47 GPa) and extending the integration time to 30 days:

$$\tau_{\mathrm{noise}} = \frac{1.10\times10^{-18}}{\sqrt{30\times24\times60\times60}} \approx 6.84\times10^{-22}\ \mathrm{Nm@0.02667Hz} \tag{24}$$

The resulting signal-to-noise ratio is:

$$SNR = \frac{1.70\times10^{-20}}{6.84\times10^{-22}} \approx 25 \quad (25)$$

This estimation represents a reasonable extrapolation based on the current experimental parameters, providing a reference direction for future exploration of gravitational interactions below the Planck mass.

Supplementary Reference

1. *MEMS Materials and Processes Handbook*. vol. 1 (Springer US, Boston, MA, 2011).
2. Tan, W.-H. *et al.* New Test of the Gravitational Inverse-Square Law at the Submillimeter Range with Dual Modulation and Compensation. *Phys. Rev. Lett.* **116**, 131101 (2016).
3. Yang, S.-Q. *et al.* Test of the Gravitational Inverse Square Law at Millimeter Ranges. *Phys. Rev. Lett.* **108**, 081101 (2012).
4. Mao, J.-J. *et al.* Residual gas damping noise in constrained volume in space-borne gravitational wave detection. *Class. Quantum Grav.* **40**, 075015 (2023).
5. Cavalleri, A. *et al.* Increased Brownian Force Noise from Molecular Impacts in a Constrained Volume. *Phys. Rev. Lett.* **103**, 140601 (2009).
6. Crank, J. *The Mathematics of Diffusion*. (Oxford University Press, 1979).
7. Adelberger, E. G., Gundlach, J. H., Heckel, B. R., Hoedl, S. & Schlamminger, S. Torsion balance experiments: A low-energy frontier of particle physics. *Progress in Particle and Nuclear Physics* **62**, 102–134 (2009).
8. Saulson, P. R. Thermal noise in mechanical experiments. *Phys. Rev. D* **42**, 2437–2445 (1990).
