# Supplementary material for: Test of the gravitational interaction between milligram masses using a miniature torsion pendulum
Source: Nat Commun. 2026 Oct 7;17:11425. doi: 10.1038/s41467-026-78187-4 (PMC13737168; doi:10.1038/s41467-026-78187-4)
Supplement: Supplementary file 2 [file 41467_2026_78187_MOESM2_ESM.pdf]

nature portfolio

Peer Review File

Test of the gravitational interaction between milligram masses using a miniature torsion pendulum

Corresponding Author: Professor Shan-Qing Yang

This file contains all reviewer reports in order by version, followed by all author rebuttals in order by version.

Attachments originally included by the reviewers as part of their assessment can be found at the end of this file.

Version 0:

Reviewer comments:

Reviewer #1

(Remarks to the Author)

Reviewer #2

(Remarks to the Author)
Summary of purpose of the article

The manuscript “Test of the gravitational interaction between milligram masses using a miniature torsion pendulum”, by Zheng-Jie An and co-authors describes an experimental verification of Newton’s inverse square law and a determination of Newton’s Gravitational Constant, G. The novelty in this experiment is the use of millimeter-sized source and test masses, which have a mass of only 7.6 mg each. In order to enhance the sensitivity of the experiment, the torque signal is shifted from DC to 26.7 mHz. This is done by a rotating attractor plate, which contains 16 identical source masses. The test mass, which describes a Cavendish beam balance, as well as the source mass plate are made from a silicon wafer by the so-called plasma deep silicon etching technology.
The gravitational torque was measured for 5 different distances between the source and test mass, ranging from 654 µm to 1.554 mm, for the verification of the inverse square law, whereas the determination of G was performed only for the closest separation. The measured value of 6.83(29)x10^-11 kg m^-3 s^-2 is in agreement to within one sigma with the CODATA-2022 value. No significant deviation for the inverse square law was detected.

General comments

The experiment is well thought and well performed with excellent results. It thus presents an important advancement in gravitational physics.

The title represents well the content of the study. I find the references acknowledge relevant former works.

The length of the manuscript is adequate and the text well written. Mayor uncertainty contributions are well documented in the extended data section.

However, I have a problem with the abstract. It rather is a motivation for the experiment. It is missing what has been measured, an outline of the method, and a summary of the results, as well as what is the outcome. I cannot find any word about “big G” or “inverse square law” in the abstract. Giving an uncertainty of “40 aN” is meaningless in my opinion if the absolute force signal is not mentioned. It is also unnecessary to compare the results to another publication (ref [1]) in the abstract. There is lot of room in the introduction section for motivation of the experiment, and a report of current state-of-the-art.

Moreover, the authors state in the abstract that “What is lacking are experiments that probe the mutual gravitational interaction between mesoscopic or microscopic objects where quantum effects dominated.” Isn’t it true that quantum effects

should dominate in the microscopic world rather than towards the mesoscopic world?

Finally, the authors take “gravity” and “gravitation” as synonyms. To my knowledge, this is not correct. Gravitation is the force that is generated by means of mass, while gravity is a combination of gravitation and centrifugal acceleration (due to the rotation of the earth w.r.t. the fixed stars). As in the experiment presented in this manuscript the nature of the force under test is gravitation, the expression gravitation should be used throughout the manuscript.

Detailed comments

Abstract:
Line 50: “… demonstrate the potential of going considerably smaller”. I cannot find in the manuscript, where this potential is described. Or do you refer to the “Outlook” where you describe how the sensitivity increases by reducing the torsion diameter to 3 µm, increasing the Q to 25000, and bringing the masses closer to 15 µm? This is not specific for your setup. At least, this should not be claimed in the abstract, as it is rather an outlook.

Main:

Introduction:
p. 2, l. 66ff: “However, measurements of gravity from classical sources in laboratory table-top settings is in contrast with the increasing attention on probing gravitational interactions originated from quantum state of source masses …” There is no contrast in my opinion. There are only two foci that are described. One focus is on improving the determination of the value of G, with the smallest relative uncertainties as possible. Therefore, big source and test masses are necessary. The second focus is the search for quantum-effects of gravitation. To this end, the source and test masses have to be reduced as much as possible. I recommend re-phrasing this sentence.

p.2, l.95: What is “plasma deep silicon etching technology”? Maybe you provide a reference to a review article?

p.2, l.55, right column: “No deviations from the Newtonian predictions are observed with…”. Why do you present here already your results (although, as stated before, in my opinion this is not the form of presenting the results about the measurement of the inverse square law)? If so, why don’t you present then also your results for G? This should go into the results section (and also into the abstract).

Experimental Setup:

p.3, l. 135: “The attractor, rotary stage, conducting membrane and alignment block are all mounted …” The conducting membrane was not mentioned before. It appears suddenly here. Maybe mention it earlier in the text, with a description of what it is for.

p.3, l. 147: “Through this procedure, the rotation axis is aligned to the pendulum center within uncertainties of 24 µm and 20 µm in the x and z directions, respectively, while the face-to-face separation along y is determined with an uncertainty of below 20 µm.” Which procedure do you mean? I cannot see any description of the procedure. Could you explain how this was done, eventually in the extended data.

Fig.1c is too small.

p.4, l. 174: “The moment of inertia of the pendulum is calculated to be I = 12.61(1) × 10−9 kg · m2 from the measured geometric parameters …” Can you explain how the parameters were measured? By CMM or CT?

Method:

p.5, figure caption of fig.2, l. 233: “… calculated Newtonian gravity …” I would rather say “… calculated (Newtonian) gravitation-induced torque …”

Systematic Effects:

p.6, table 1: Why does the measurement of the test mass have higher uncertainties than the source mass, although they have the same dimensions?

p.6, 272, right column: “The remanent magnetization intensity of a silicon sample is measured using a femtotesla magnetometer …” Where did this measurement take place? In an extra (shielded) room or on the laboratory table?

Results and Discussion:

p.8, l.359: “…designed to access regimes relevant to quantum coherence.” Can you give a number here for which masses this should happen?

p.8, l.375 ff: Can you say how many measurements you have taken for the evaluation of G (I could count the dots in fig.3, but it is better to read the number somewhere)?

Outlook:

p.8, l.402: “Probing gravity between objects with masses below the Planck scale (mp ≈ 22 µg) is crucial for exploring its quantum nature.” Do you expect quantum-gravitational effects for masses of mp? Why? How would you notice such effects in your experiment? Would both masses (source and test) need to be of that order of magnitude, or is it sufficient that one is of this mass?

p.8, l. 368, right column: “At the same time, by shortening the separation from the current 654 µm to ~ 15 µm, a 10^5 improvement in signal-to-noise ratio can be obtained …” How did you calculate the number 10^5?

Extended data figure 1: “… 0.487 mm-thick pendulum …” mm not in italic!

Extended data figure 4: “Feedback voltage [V]” The unit does not go into squared brackets.

Extended data figure 6: In this figure caption you write often “k_e” with “e” non-italic, whereas throughout the manuscript it is in italic. (also “f_d” and “\delta tau_e” in this figure caption). Be consistent.

Extended data figure 8: In the figures there are spaces in the text of the legends, e.g. “vac uum”

One last question: Did you try to rotate the attractor with other frequencies, in order to see if you get the same results?

Recommendation for the editor

This article describes a very interesting experiment and is surely interesting to a broad physics community. So, it is worth its publication, because the measurement of the gravitational constant is still a challenging task and is worth to be investigated on a broad frequency scale, as well as for small masses.

A shortcoming is clearly the abstract. This definitely should be re-written.
If the authors consider my comments and questions, then I can recommend a publication.

Reviewer #3

(Remarks to the Author)
The authors report the measurement of the Newtonian gravitational force from a 16-spoked silicon source mass using a ~7 mg test mass suspended as a torsional pendulum. They infer the Newtonian gravitational constant G --- the least precisely known of all the fundamental constants --- from their measurement, a result consistent with the latest CODATA value.

The significance of the experiment is that it is the smallest test mass used in the measurement of Newtonian gravity yet. Although the authors claim, somewhat loosely, that this has ramifications for tests of quantum gravity etc, these should be taken with a grain of salt. However, in Extended Data Figure 9, they contextualize their result in terms of limits on Yukawa-type modifications of gravity. Although not competent in an absolute sense, the combination of this low mass for the test mass makes such a test scientifically interesting.

There are however some comments/issues that would significantly aid the manuscript:

-- The most serious deficiency of the manuscript is as follows: Fig. 2(f) is the main measurement result, purporting to show the gravitational torque signal at 16f. But from looking at the spectrum, it is impossible to see whether the arrow the authors point to (at 16f) is some noise spike coming from the variance of the spectrum estimate, or an actual signal! The authors need to do some very detailed statistical analysis --- starting with studying the histogram of the spectral estimates --- to show that this spike is indeed a signal. Details of such analysis needs to be provided in the supplementary information as well, perhaps with the data and code used to analyze it.

-- The authors actually claim that the experiment evidences gravitational attraction between two 7.6 mg masses. But this would only be true if the source mass contribution is dominated by each of its paddles, and very little of the spokes and central mass. Since details of the geometric integration to estimate the Newtonian force is insufficiently detailed, it is unclear whether this claim is true.

-- Indeed, the manuscript seems to suggest that the 16f signal is dominated by other effects, with a sub-dominant contribution from Newtonian gravity, which already refutes the claim that the 16f signal can only arise from gravitational coupling; any synchronous effect at f could have a 16f component.

-- Related: the extended data contains very many measurements of various extraneous causes that might affect the experiment. But the effect of these causes are much less clear from these plots, or the brief discussion in the main text. As simple examples:
(a) if the source mass is rotated at a frequency f, any imbalance in the spokes can produce an acceleration at 16f, which, through the mechanical contact to the test mass, can produce an acceleration (and therefore torque) signal at 16f.

(b) the magnetic field estimates seem to focus on uniform static fields; what about rotating fields from the source mass' motor windings?
(c) What about forces due to residual gas pressure? Rotation of the source causes gas motion at all harmonics of the rotation frequency, which could affect the the pendulum and/or (above point) membrane.
(d) what about potential 16f fluctuations in the capacitive bias?

Loopholes such as this are not independently closed. Perhaps the authors rely on the consistency of the inferred value of G to bolster some confidence (but see next), but presumably, the point of these independent measurements is to act as an independent check; this they do not do yet.

-- But even the inferred G value has a blind-spot: how is the mass density of the source/test masses ascertained? (Indeed it seems like their density uncertainties are treated differently; why?)

-- There is no control experiment on the rotation frequency f. For example:
(a) a sequence of measurements where f is varied, and the inferred gravitational coupling is shown to be frequency independent
(b) or measured amplitudes of the other gravitational harmonics
(c) or measured phase of the 16f signal relative to the known phase from the source mass encoder; it would seem like the error signal already contains this information, which the authors could analyze.

-- What about translation motion of the pendulum-membrane gap? The authors measurements appear to only bound the tilt motion, whereas translation motion remains unbounded, and could definitely cause a spurious signal.

-- How is the source mass electrically neutralized?

-- The fabrication and dimensional precision achieved here for the test and source mass are very important details, and in fact one of the main technical advances. But these details are missing in the paper, without which, the reader's confidence in the error estimates arising from dimensional uncertainty is low. So I suggest:
(a) showing a photograph of the test and source mass in Fig. 1 (perhaps with additional angles in the extended data)
(b) adding a section in a supplementary information detailing the full fabrication details of the device in replicable form.

-- Extended Data Figure 9 would benefit from being moved to the main text, and the message surrounding it explicated further there (as well as in the abstract and introduction).

Version 1:

Reviewer comments:

Reviewer #1

(Remarks to the Author)
I thank the authors for considering my and the othor reviewers' comments. Upon examination, the revised manuscript appears to substantially addressed most of concerns that were raised
With regards to my comments:

(major)

1. Plaigarism in the abstract and introduction has been removed.

2. Abstract and intro have been substantially revised and now better motivate the experiment.

3. Presentation of the 16f peak in Fig. 2 has been substantially improved, including new data.

4. Technical overload concerns have been partially addressed by removing some material to the appendix. I still think a Nature-Comm/general-audience reader would appreciate better signposting (see original comment), but this is perhaps a matter of taste.

(minor)

1. Authors claim to have done a grammar check. I trust them.

2-20. Addressed.

21. Partially addressed. The authors have only cited levitated experiments, whereas I suggested highlighting a broader ecosystem the including torsion pendula (similar to the authors') and other tethered optomechanical systems.

Remaining concerns:

1. It is unclear what "General relativity (GR) and the standard model (SM) are fundamentally incompatible" means. This seems to be an overstatement.

2. I don't quite follow the logic of "... Gravitational measurements where both the source mass and the test mass are in the small-mass regime remain relatively scarce. Consequently, it remains an open question whether the gravitational law still follows Newtonian gravitation when the objects become sufficiently small or even enter the quantum regime." Theoretically, the 1/r^2 is well-motivated and expected. What small-mass gravitational measurements provide is an opportunity to probe deviations from this expectation, rather to solve an open question, per se.

3. In the outlook "To observe gravitation-induced quantum effects, both the source mass and the test mass should ideally fall within the range of 10^(-15)to 10^(-13)kg." seems to be referring to a particular set of proposals. If so, please cite, or all address the narrow range.

4. See comment 21, above

Reviewer #2

(Remarks to the Author)
Dear authors,

Thank you for considering all my comments and suggestions. Especially, I am gratefully for conducting an additional experiment at another frequency, which showed consistency.

I agree with the revised manuscript and recommend a publication.

I finally have a few minor comments. It is up to the authors whether they address them or not.

In main manuscript
1) Abstract (also within the main text): Why do you give a 2-sigma uncertainty for the uncertainty of 46 aN, while the uncertainty for G is given as 1-sigma?
2) P.7, right column: "… (4.8% relative error), …" This should be "relative uncertainty". If you know that this is an error, then you can correct it. Ana dadd a space between 4.8 and %, as % is teated as a unit.

In Supplementary Information
1) 1.1 Data preprocessing methods: These should be negligible contributions, but can you mention which kind of voltmeter you have used, and whether it was calibrated beforehand? Did you consider the sinc-error? What about clock stability?
2) 1.5 Conversion to torque and error propagation: For the electrostatic control coefficient \beta, can you please write the units to each term, not just in parenthesis after the equation.
p.5, l.113: the terms (\tau) below the square root must be squared (not the \cos and\sin)
3) 2.4 Measurement of …, p.7, l.162: "Using a vision measuring machine …" Throughout the text you use the term "Video measuring machine". I this a different system?
l. 163: "… is measured to be 0.268 mm" Can you give here an uncertainty, as you have done for the other measurements?

Reviewer #3

(Remarks to the Author)
The authors have thoroughly addressed all the questions and comments I had raised during the previous round of review.

Open Access This Peer Review File is licensed under a Creative Commons Attribution 4.0 International License, which permits use, sharing, adaptation, distribution and reproduction in any medium or format, as long as you give appropriate credit to the original author(s) and the source, provide a link to the Creative Commons license, and indicate if changes were made.
In cases where reviewers are anonymous, credit should be given to 'Anonymous Referee' and the source.
The images or other third party material in this Peer Review File are included in the article's Creative Commons license, unless indicated otherwise in a credit line to the material. If material is not included in the article's Creative Commons license and your intended use is not permitted by statutory regulation or exceeds the permitted use, you will need to obtain permission directly from the copyright holder.
To view a copy of this license, visit https://creativecommons.org/licenses/by/4.0/

Response on Nature Communications manuscript

Dear Reviewers,

We sincerely thank you for the time and thoughtful consideration you have devoted to reviewing our manuscript. Your constructive criticism and insightful suggestions have been invaluable in strengthening this work. In response to your feedback, we have thoroughly revised the manuscript to address all points raised, incorporating additional experimental data and extended analyses where necessary.

Below, we provide a detailed point-by-point response to all comments; our replies are indicated in **blue**. All textual revisions are marked in **red** in the updated PDF, and the supplementary information is included as a separate document. We are deeply grateful for your expert guidance throughout this review process.

Sincerely,

Shan-Qing Yang,
Professor, School of Physics and Astronomy & TianQin Research Center
Sun Yat-sen University
Zhuhai, 519082, China

Outline:

Responses to Reviewer #1: Page 2---Page 13

Responses to Reviewer #2: Page14---Page 27

Responses to Reviewer #3: Page28---Page 50

Responses to Reviewer #1: (marked as blue)

Reviewer #1 (Remarks to the Author):

In "Test of the gravitational interaction between milligram masses using a miniature torsion pendulum," the authors describe a miniature Cavendish-type experiment in which the gravitational force between two lithographically defined source masses is measured and confirmed to agree with the Newtonian inverse square law. The absolute mass(~ 8 mg) of the source masses is 10-times smaller than a similar experiment conducted by Westphal et. al. 2021 (reference [1]) and represents important step towards Planck mass-scale experiments (~0.1 mg) proposed to test the quantum nature of gravity.

The reported results will garner significant interest and are deserving of being published in Nature Communications, in my opinion. The authors have been developing variants of the apparatus for over a decade. While I am not an expert in this measurement, I found its treatment authoritative.

That said, I have some concerns about presentation of ideas and data in the manuscript that I believe should be addressed before publication. These are listed below, followed by relatively minor comments.

1. Textual overlap with [1]

Several passages in the abstract and introduction contain significant overlap with [1], either in wording or narrative structure. For instance, in the abstract:

(Westphal) "Because quantum coherence is easily lost for increasing system size, it is important to isolate gravity as a coupling force for as small objects as possible."

(An) "Since the quantum coherence is easily lost with the increasing size or mass of the system, it is essential to characterize gravity for as small objects as possible."

And

(Westphal) "Our results extend the parameter space of gravity measurements to small, single source masses and low gravitational field strengths."

(An) "Our results extend gravity experiments to the regime of small gravitational

sources masses at the atto-newton level, and demonstrate the potential of going considerably smaller"

While some overlap is to be expected, the authors are encouraged to revise the abstract and introduction in their own language (not just rearranging words), and to be vigilant against copying.

2. Unclear motivation and relation to quantum experiments

In my opinion, the abstract in introduction do not make it sufficiently clear why this experiment is important, especially in relation to quantum experiments. For example

(1) the abstract begins with a tautology: Probing the interplay between quantum mechanics and gravity... offers a... pathway to explore the quantum nature of gravity,

(2) the connection between small mass and quantum gravity is missing in the abstract: "What is lacking are experiment that probe the mutual gravitational interaction between mesoscopic or microscopic objects where quantum effects dominated. Here, we report a direct measurement of the gravitational interaction between two 7.6-mg silicon wafers using a miniature torsion pendulum..."

(3) the pivotal "however" statement in the intro is a non-sequitur (false/unsupported contrast): However, measurements of gravity from classical sources in laboratory table-top settings is in contrast with the increasing attention on probing gravitational interactions originated from quantum state of source masses.

Response to the above three questions.

We thank the reviewers for their professional evaluations and constructive suggestions. In response, we have revised the abstract and introduction to reduce the emphasis on quantum gravity and to focus more on the precise measurement of gravitational forces acting on small objects and the determination of the gravitational constant *G*. The revised abstract and introduction are provided below: *"Precision tabletop measurements of gravitational force are important for probing the fundamental nature of gravitation. Although gravitational experiments have spanned test masses from macroscopic objects to individual quantum systems, the mutual*

*gravitational interaction between small—particularly microscopic—masses remains largely unexplored. Recent advances have extended such studies into the sub-100 mg regime, however, probing gravitation at still smaller masses continues to pose significant challenges. Here, we report a measurement of gravitational interaction between two 7.6 mg silicon wafers using a miniature Cavendish-type torsion balance combined with a rotating mass-modulation technique. Over separation distances from 0.654 to 1.554 mm, the measured forces agree with Newtonian predictions, achieving a maximum uncertainty of 46 a*N *(2σ) and enabling constraints on Yukawa-type deviations from Newtonian gravitation. From these data, we determine the gravitational constant* $G = 6.67(32) \times 10^{-11}\ m^3\ kg^{-1}\ s^{-2}$ *(1σ). This work employs the smallest test and source masses ever used in measurements of Newtonian gravitational interaction, representing a significant step toward bridging the classical and quantum regimes of gravitation."*

In the Introduction, the sentence "……However,……since the quantum coherence is easily lost with the increasing size or mass of the system" is revised as *"……In current experiments, to enhance the gravitational interaction signal, the source mass is typically chosen to be a massive object (including celestial bodies), while the test mass varies from macroscopic objects to individual quantum systems, depending on the experimental method and expected precision. However, gravitational measurements where both the source mass and the test mass are in the small-mass regime remain relatively scarce. Consequently, it remains an open question whether the gravitational law still follows Newtonian gravitation when the objects become sufficiently small or even enter the quantum regime. This work targets the largely unexplored gravitational interaction between small-mass objects and conducts precision measurements, aiming to provide experimental evidence that may shed light on the nature of gravitation and lay a foundation for future studies on even smaller-mass systems."*

3. Data presentation in Fig. 2

The experimental data presented in Fig. 2 is compelling as a demonstration that the authors have characterized their torsion pendulum and feedback control system; however, it does not clearly highlight the signal of interest: a small coherent tone at $16f_s$ buried in the broadband noise spectrum in panels (d,e). By contrast, in Fig. 2 of Westphal [1], the coherent signal at f_s and $2f_s$ rises visually above the noise and may be directly quantified in both displacement and force units, by comparing to the left/right plot axis. The quality of the processed data in Fig. 3 suggests that a cleaner visualization of the raw experimental data is possible. The authors are encouraged to do so in Fig. 2, possibly by using magnified insets in panels (d) and (e), or by showing representative noise spectra in Fig. 3.

We appreciate the reviewer's valuable suggestions. The signal in the power spectrum density of Figure 2(f) is not readily discernible for two primary reasons. First, while the Westphal experiment utilized 90-mg objects, our experiment employed two 7.6-mg objects as test mass and source mass, respectively. Consequently, the gravitational signal in our experiment is approximately 140 times weaker than that in the Westphal experiment. Second, to limit the temperature rising of the turntable in vacuum, we implemented an intermittent operation mode: continuous rotation of the turntable for 13 full revolutions (7,800 s), followed by a 3,600 s pause, then resumption of rotation. As a result, figure 2(f) presents the power spectrum analysis based on a single 7,800-second dataset, the limited integration time accounts for the insufficiently prominent gravitational signal at $16f_d$.

In response to the reviewer's suggestion, we have enhanced the visibility of the $16f_d$ gravitational signal at s=0.654 mm by computing the averaged power spectrum from six independent 7,800 s data segments, which effectively suppresses random fluctuations. Additionally, as shown in the revised Figure 2(e), we expanded the plot area to clearly reveal a distinct gravitational signal at $16f_d$. Further contrast enhancement would require extending the measurement time.

For comparison, an identical measurement was performed at a larger separation ($s \approx 3$ mm), where the Newtonian gravitational torque at $16f_d$ is theoretically expected to fall below the torsion balance noise floor and thus remain undetectable in the power spectrum. The measurement confirms this expectation: a prominent signal appears only at the drive fundamental frequency f_d, with a magnitude consistent with the close-separation result, while no discernible signal is present at $16f_d$. This control test further corroborates the reliability of the close-separation measurements. In the frequency band above 30 mHz, some random peaks are likely attributable to membrane frame vibrations induced by the rotary stage and ground vibration, which represents an area requiring improvement in subsequent experiments.

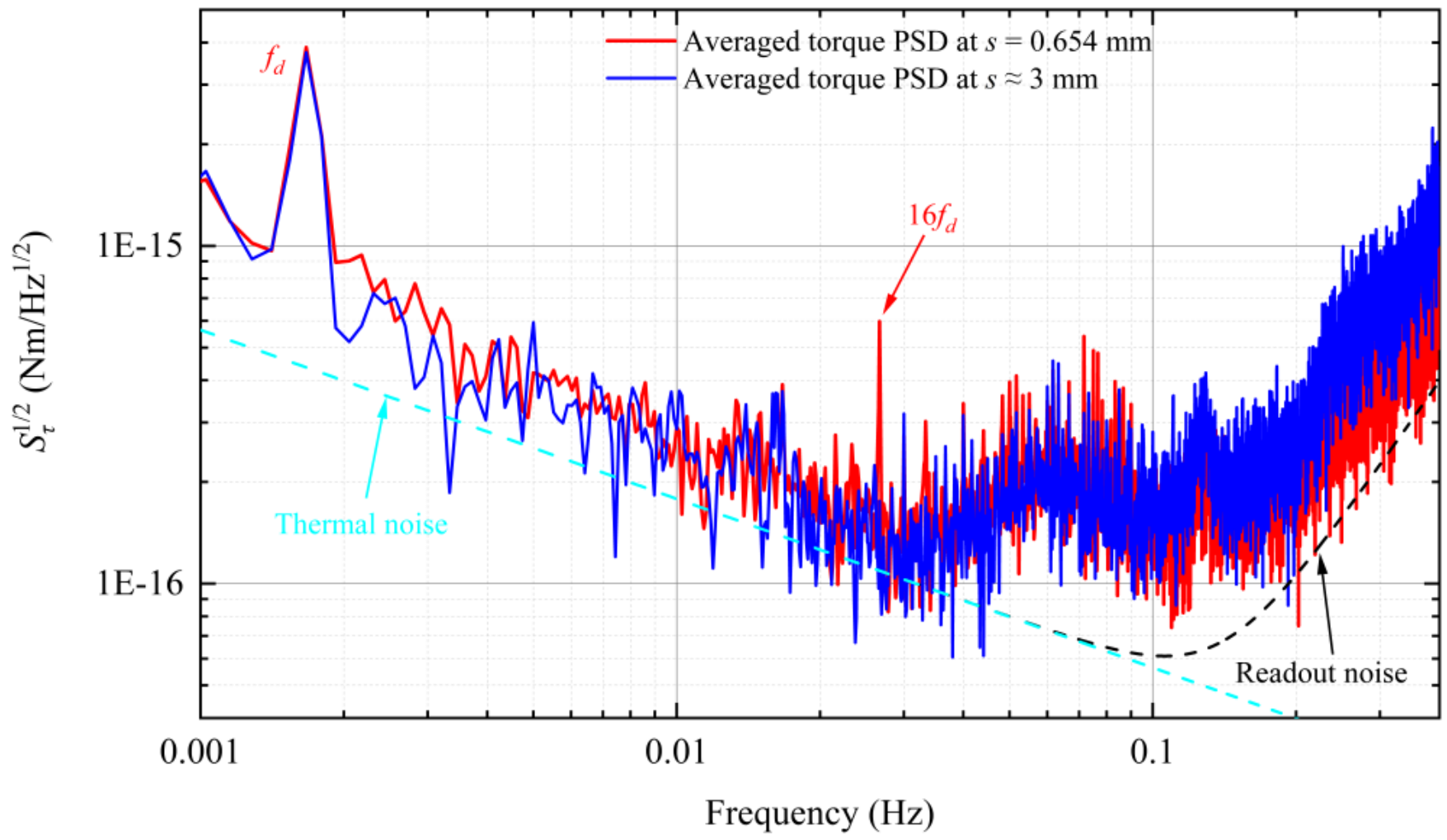

Figure 2(e): Comparison of averaged torque PSD at separations of 0.654 mm and 3 mm. At s = 0.654 mm, a distinct gravitational signal is clearly observed at $16f_d$. Conversely, at the larger separation of $s \approx 3$ mm, the Newtonian gravitational torque at $16f_d$ is theoretically expected to fall below the torsion balance noise floor, rendering it undetectable in the power spectrum. The experimental results confirm this theoretical expectation: a prominent signal appears only at the drive fundamental frequency f_d, with magnitude consistent with the close-separation measurement, while no discernible signal is present at $16f_d$. This control test further corroborates the reliability of the close-separation measurements.

4. Technical overload in Methods section

The paper is written in the style of a traditional metrology paper, with great attention to technical detail. This is fine and, for posterity, important; however, to improve

readability for non-specialists (a purpose for publishing in Nature Communications), it is advisable that the authors buttress technical discussion with explanations of what details are most important and why. The Methods section stands out as unusually technical for a Nature paper. Much of this content could in principle be off-loaded to an appendix. If the authors are intent on keeping it in the main text--which is reasonable, given that this is a precision metrology paper--I recommend sub-sectioning and/or prefacing each paragraph with a "why this important" sentence, similar to how the Systematic effects section is constructed. The authors might in any case consider lightening and/or moving some technical details to the appendix.

Thanks to the reviewer and the valuable suggestion. In the Method section, we have condensed the description of technical details and relocated the determination process of the feedback gain and the electrostatic coefficient to the Extended Data Figure 4.

5.Minor comments in rough chronological order

(1). (general) The text is adequately written, especially the technical sections, but could use a once over for grammatical correctness. Some examples are given below. They are not exhaustive. (abstract) See comments 1 and 2 above.(introduction, especially paragraph 1) See comments 1 and 2 above.

Thanks to the reviewer, we have addressed the reviewer's comments above.

(2). (47, col 1) "mass used in the previous test [1]" implies [1] is the only test preceding the authors'. If this is correct, then fine. If not, then "the" should be replaced with "a", or the sentence reconstructed.

Thanks to the reviewer, this sentence has been deleted.

(3). (49, col 1) "...small gravitational masses at the atto-Newton level.." Since masses don't have units of Newtons, this statement is unclear. Consider revising.

Thanks to the reviewer, this sentence has been deleted.

(4). (57, col 1) "GR and the SM... have so far passed all experimental tests... However, the incompatibility of the two theories provides significant motivation." The second sentence is premised on the unstated claim that GR and SM are incompatible. This should be stated explicitly with support from a reference.

We thank the reviewer for this suggestion. In response, we have revised the sentence and incorporated a relevant citation in the manuscript. *"General relativity (GR) and the standard model, the two cornerstones of modern physics, have withstood every experimental test to date. Yet their fundamental incompatibility provides a compelling impetus for probing the quantum nature of gravitation.[*]"*

[*]Karen Crowther, Why Do We Want a Theory of Quantum Gravity? Publisher: Cambridge University Press, Print publication: 27 March 2025, DOI: https://doi.org/10.1017/9781108878074.

(5). (58, col 2) "50 times enhanced than" ⇒ "50 times enhanced over"

Thanks to the reviewer, this sentence has been deleted.

(6). (65, col 2) "a schematic view of the new experimental design" Readers will not necessarily be familiar with the authors previous work, so "new" needs to be qualified or removed.

Thanks to the reviewer, we've made the revision.

(7). (67, col 2) " Φ - shaped" is unclear. Is Φ - shaped a well-known torsion pendulum geometry? An observational aside? Since the phrase is not used elsewhere, consider removing it.

Thanks to the reviewer, we've made the revision.

(8). (70, col 2) Consider cross-referencing Table 1 when introducing device dimensions for the first time.

Thanks to the reviewer, we've made the revision.

(9). (93, col 2) "The attractor is designed as "ferris-wheel-shaped" to ensure its structural strength" Similar to the above comment, it's unclear why pointing out that the attractor looks like a ferris wheel adds useful information. The sentence implies that there is a connection between ferris wheels and structural strength. This may be case, but not obviously. Consider revising this sentence for concision and clarity.

Thanks to the reviewer, the sentence has revised to *"The attractor is designed as a 16-fold symmetrical structure."*

(10). (150, col 1) "The rotation axis of the attractor is aligned to the rotary stage within 40μm using an optical imaging instrument." It is unclear what is meant by "optical imaging instrument." Consider stating.

Thanks to the reviewer, the "optical imaging instrument" has revised to ***"Video Measuring Machine",*** which employing CCD digital image processing technology, enhanced by computer screen measuring techniques and geometrical calculation software, enables efficient dimensional measurements with ±3 μm accuracy. An in-situ measurement photograph is presented in Extended Data Fig. 1.

(11). (134, col 2) "A 50-μm-thick, gold-coated conducting membrane..." Consider stating what the membrane substrate material is. Silicon nitride?

Thanks to the reviewer, the substrate material is monocrystalline silicon wafer. The sentence has revised to *"A 50-μm-thick, gold-coated conducting monocrystalline silicon membrane..."*

(12). (184, col 1) Consider changing the section title Method to the (more typical)

Methods.

Thanks to the reviewer, we've made the revision.

(13). (196, col 1) "...τ(t) is the external torque" Does $\tau(t)$ include thermal noise? If so, consider stating so, or else consider including an additional thermal torque in Eq. 1.

Thanks to the reviewer, τ(t) has included the thermal noise, and has been clarified in the revised manuscript.

(14). (252, col 1) "The $16\omega_d$ Newtonian torque is calculated via numerical integration over separations from $s = 0.654$ to 1.554mm" It is unclear what is being numerically integrated over. Please clarify.

We appreciate the reviewer's comment regarding this ambiguous description. The integration is performed over the volumes of all relevant components of both the pendulum and the attractor, applying $U_{Nij} = G\rho_i\rho_j \int_{V_i}\int_{V_j} \frac{1}{|\vec{r_i}-\vec{r_j}|} dV_i dV_j$ for each separation, rather than over separations. We have revised the sentence to:

"The 16ωd Newtonian torque is calculated via numerical integration of the Newtonian gravitational interaction over all volume elements of the torsion pendulum assembly and all 16 source masses with their supporting struts on the rotating attractor, using the measured geometries and densities of each component. Separate integrations are performed for each separation from s=0.654 mm to 1.554 mm."

(15). (240, Fig. 2 caption) "The transformed torque PSD." It is unclear what "transformed" means. I believe the authors mean the "inferred" or "apparent" torque PSD.

Thanks to the reviewer, "The transformed torque PSD……" has been changed to *"The derived PSD of the torque through the measured feedback voltage according the Eq.2.……"*.

(16). (266, Table 1) Can the authors provide a reference or a footnote for how the density uncertainty, $2313.5 \pm 11.9\mathrm{kg/m^3}$ was obtained? (It seems reasonable, but oddly specific to a nonspecialist.)

We thank the reviewer for this suggestion. In response, we have added a footnote below Table 1 with the following clarification: *"The silicon wafer density value represent an average calculated from multiple samples cut from the same wafer used for both the torsion pendulum and source masses, determined by measuring their masses and dimensions"*

(17). (301, col 2) Can the authors provide a reference or commentary on how the " $\delta T < 2.1\mu\mathrm{K}$ temperature fluctuations at $16\omega_d$" was determined?

We thank the reviewer for this suggestion. The temperature fluctuation amplitude δT at $16\omega_d$ was extracted from 7,800-second segments collected during each experimental run. A typical value of 2.1 μK was obtained across multiple measurement sets, with corresponding procedures added to Extended Data Fig. 8.

(18). (Fig. 3) Minor comment: consider changing panel B red point label "Measurement result of G" to Measured/Inferred/Calculated G, since, as described in the text and caption, these are indirect measurements.

We thank the reviewer for this suggestion, we've made the revision.

(19). (351, col 2) For passage "Beyond confirming Newtonian gravity... small masses may help bridge between... lab tests of classical gravity and future experiments designed to access regimes relevant to quantum coherence" it would be helpful to have references supporting claims.

Thanks to the reviewer, two references have been cited in the revised manuscript.

[*] Al Balushi, A., Cong, W. & Mann, R. B. Optomechanical quantum cavendish experiment. Phys. Rev. A 98,043811 (2018).

[*] Bose, S. et al. Massive quantum systems as interfaces of quantum mechanics and gravity. Rev. Mod. Phys. 97,015003 (2025).

(20). (361, col 1) "However, this is not generally advantageous" What does "this" refer to?

We appreciate the reviewer's clarification. The term "this" refers to "Conducting Gravitational Experiments with Small-Mass Objects." This section addresses the challenges in such experiments: the inherently weak gravitational effects of small masses result in poor signal-to-noise ratios, making them less effective for improving the precision of classical gravitational measurements. And the sentence has been revised to *"However, small mass objects not generally competitive in improving the accuracy of classical gravitational experiments, such as …… "*

(21). (Outlook) The outlook highlights strategies to improve the authors' apparatus (including reducing the fiber size and moving from metal-alloy to quartz) toward the long-term goal of Planck-mass experiments. This is interesting and appropriate; however, for a Nature audience, the authors might also consider contextualize their outlook within the broader ecosystem of mg-scale experiments and sensors aimed at precision gravity measurements---including torsion pendula (both lithographic and assembled), levitated systems, and optomechanical platforms---currently facing similar challenges and pursuing similar goals.

We are grateful to the reviewer for the helpful suggestion. In response, we have inserted the sentence *"Alternatively, approaches employing optically, magnetically, or otherwise levitated masses offer promising and highly anticipated avenues[**]"* before the final sentence, and we have updated the references accordingly.

[*].Fuchs, T. M. et al. Measuring gravity with milligram levitated masses. Sci. Adv. 10, eadk2949 (2024)

[*].U. Delić, M. Reisenbauer, K. Dare, D. Grass, V. Vuletić, N. Kiesel, and M.

Aspelmeyer, Cooling of a levitated nanoparticle to the motional quantum ground state, Science 367,892 (2020).

[*].Tebbenjohanns, F., Frimmer, M., Jain, V., Windey, D. & Novotny, L. Motional sideband asymmetry of a nanoparticle optically levitated in free space. Phys. Rev. Lett. 124, 013603(2020).

[*].P. R. Yin, R. Li, C. J. Yin, X. Y. Xu, X. Bian, H. Xie, C. K. Duan, P. Huang, J. H. He, and J. F. Du, Experiments with levitated force sensor challenge theories of dark energy, Nat. Phys. 18,1181 (2022).

[*].C. W. Lewandowski, T. D. Knowles, Z. B. Etienne, and B. D'Urso, High-sensitivity accelerometry with a feedback-cooled magnetically levitated microsphere, Phys. Rev. Appl. 15,014050 (2021).

Responses to Reviewer #2: (marked as blue)

Reviewer #2 (Remarks to the Author):

1.Summary of purpose of the article.

The manuscript “Test of the gravitational interaction between milligram masses using a miniature torsion pendulum”, by Zheng-Jie An and co-authors describes an experimental verification of Newton’s inverse square law and a determination of Newton’s Gravitational Constant, G. The novelty in this experiment is the use of millimeter-sized source and test masses, which have a mass of only 7.6 mg each. In order to enhance the sensitivity of the experiment, the torque signal is shifted from DC to 26.7 mHz. This is done by a rotating attractor plate, which contains 16 identical source masses. The test mass, which describes a Cavendish beam balance, as well as the source mass plate are made from a silicon wafer by the so-called plasma deep silicon etching technology.

The gravitational torque was measured for 5 different distances between the source and test mass, ranging from 654 μm to 1.554 mm, for the verification of the inverse square law, whereas the determination of G was performed only for the closest separation. The measured value of $6.83(29)\times 10^{-11}$ kg m^{-3} s^{-2} is in agreement to within one sigma with the CODATA-2022 value. No significant deviation for the inverse square law was detected.

2.General comments

The experiment is well thought and well performed with excellent results. It thus presents an important advancement in gravitational physics. The title represents well the content of the study. I find the references acknowledge relevant former works. The length of the manuscript is adequate and the text well written. Mayor uncertainty contributions are well documented in the extended data section.

However, I have a problem with the abstract. It rather is a motivation for the experiment. It is missing what has been measured, an outline of the method, and a summary of the results, as well as what is the outcome. I cannot find any word about

"big G" or "inverse square law" in the abstract. Giving an uncertainty of "40 aN" is meaningless in my opinion if the absolute force signal is not mentioned. It is also unnecessary to compare the results to another publication (ref [1]) in the abstract. There is lot of room in the introduction section for motivation of the experiment, and a report of current state-of-the-art.

Moreover, the authors state in the abstract that "What is lacking are experiments that probe the mutual gravitational interaction between mesoscopic or microscopic objects where quantum effects dominated." Isn't it true that quantum effects should dominate in the microscopic world rather than towards the mesoscopic world?

We are grateful to the reviewer for the helpful suggestion. Following the reviewers' constructive suggestions, we have revised the abstract as follows: *"Precision tabletop measurements of gravitational force are important for probing the fundamental nature of gravitation. Although gravitational experiments have spanned test masses from macroscopic objects to individual quantum systems, the mutual gravitational interaction between small—particularly microscopic—masses remains largely unexplored. Recent advances have extended such studies into the sub-100 mg regime, however, probing gravitation at still smaller masses continues to pose significant challenges. Here, we report a measurement of gravitational interaction between two 7.6 mg silicon wafers using a miniature Cavendish-type torsion balance combined with a rotating mass-modulation technique. Over separation distances from 0.654 to 1.554 mm, the measured forces agree with Newtonian predictions, achieving a maximum uncertainty of 46 aN (2σ) and enabling constraints on Yukawa-type deviations from Newtonian gravitation. From these data, we determine the gravitational constant $G = 6.67(32) \times 10^{-11}\ m^3\ kg^{-1}\ s^{-2}$ (1σ). This work employs the smallest test and source masses ever used in measurements of Newtonian gravitational interaction, representing a significant step toward bridging the classical and quantum regimes of gravitation."*

3.Finally, the authors take "gravity" and "gravitation" as synonyms. To my knowledge,

this is not correct. Gravitation is the force that is generated by means of mass, while gravity is a combination of gravitation and centrifugal acceleration (due to the rotation of the earth w.r.t. the fixed stars). As in the experiment presented in this manuscript the nature of the force under test is gravitation, the expression gravitation should be used throughout the manuscript.

We are grateful to the reviewer for the valuable suggestion, the expression "gravitation" is more suitable for this work, we have made corrections throughout the entire text.

4.Detailed comments

(1). Abstract:Line 50: "… demonstrate the potential of going considerably smaller". I cannot find in the manuscript, where this potential is described. Or do you refer to the "Outlook" where you describe how the sensitivity increases by reducing the torsion diameter to 3 μm, increasing the Q to 25000, and bringing the masses closer to 15 μm? This is not specific for your setup. At least, this should not be claimed in the abstract, as it is rather an outlook.

Thanks to the reviewer, we have revised the abstract.

(2). Introduction: p. 2, l. 66ff: "However, measurements of gravity from classical sources in laboratory table-top settings is in contrast with the increasing attention on probing gravitational interactions originated from quantum state of source masses …" There is no contrast in my opinion. There are only two foci that are described. One focus is on improving the determination of the value of G, with the smallest relative uncertainties as possible. Therefore, big source and test masses are necessary. The second focus is the search for quantum-effects of gravitation. To this end, the source and test masses have to be reduced as much as possible. I recommend re-phrasing this sentence.

We are grateful to the reviewer for the valuable suggestion. The sentence is revised

as *"……In current experiments, to enhance the gravitational interaction signal, the source mass is typically chosen to be a massive object (including celestial bodies), while the test mass varies from macroscopic objects to individual quantum systems, depending on the experimental method and expected precision. However, gravitational measurements where both the source mass and the test mass are in the small-mass regime remain relatively scarce. Consequently, it remains an open question whether the gravitational law still follows Newtonian gravitation when the objects become sufficiently small or even enter the quantum regime. This work targets the largely unexplored gravitational interaction between small-mass objects, aiming to provide experimental evidence that may shed light on the nature of gravitation and lay a foundation for future studies on even smaller-mass systems."*

(3). p.2, l.95: What is "plasma deep silicon etching technology"? Maybe you provide a reference to a review article?

Thanks to the reviewer. The "plasma deep silicon etching technology" is a mature semiconductor manufacturing process, a review article was cited to provide a comprehensive introduction to this technology.

[*].C. Li, Y. Yang, R. Qu, X. Cao, G. Liu, X. Jin, Y. Liu, S. Liu, W. Jiang, X. Zhang, Recent advances in plasma etching for micro and nano fabrication of silicon-based materials: a review, *Journal of Materials Chemistry C*, 2024, Vol.12, pp.18211-18237.

(4). p.2, l.55, right column: "No deviations from the Newtonian predictions are observed with…". Why do you present here already your results (although, as stated before, in my opinion this is not the form of presenting the results about the measurement of the inverse square law)? If so, why don't you present then also your results for G? This should go into the results section (and also into the abstract).

We are grateful to the reviewer for the valuable suggestion. we have made the following revisions, as "……*Here we report a precision measurement of the*

gravitational interaction between two milligram-mass silicon wafers (each 7.6 mg) using a closed-loop torsion balance, which achieves torque and force noise floors of approximately 0.1fNm/ √Hz and 4fN/ √Hz, respectively, near the 30 mHz frequency band. Over mass-to-mass separations ranging from 0.654 to 1.554 mm, we compare our measured gravitational forces with Newtonian predictions and constrain possible Yukawa-type deviations from Newtonian gravitation. From these data, we determine the gravitational constant G. "

Experimental Setup:

(5). p.3, l. 135: "The attractor, rotary stage, conducting membrane and alignment block are all mounted …" The conducting membrane was not mentioned before. It appears suddenly here. Maybe mention it earlier in the text, with a description of what it is for.

Thanks to the reviewer, we've made the revision.

(6). p.3, l. 147: "Through this procedure, the rotation axis is aligned to the pendulum center within uncertainties of 24 μm and 20 μm in the x and z directions, respectively, while the face-to-face separation along y is determined with an uncertainty of below 20 μm." Which procedure do you mean? I cannot see any description of the procedure. Could you explain how this was done, eventually in the extended data.

We thank the reviewer for the suggestion. A similar procedure has been employed in our previous work (Refs. 5, 31), so it was not described in detail in the main text. Following the reviewer's good suggestion, we have now included a detailed description of the procedure in the Supplementary Information Section 2.

(7). Fig.1c is too small.

We thank the reviewer. To address this, we have moved Fig. 1c from the main text to the Extended Data Fig. 1 and added additional photographs of the apparatus from different viewing angles in the revised manuscript.

(8). p.4, l. 174: “The moment of inertia of the pendulum is calculated to be I = 12.61(1) × 10^−9 kg • m^2 from the measured geometric parameters …” Can you explain how the parameters were measured? By CMM or CT?

We thank the reviewer for the suggestion. The length and width of the pendulum were measured using a Video Measuring Machine (Model QVP5040T, Guangdong Longtian Intelligent Instrument Co., Ltd., China), with a measurement accuracy of ±3μm. The thickness was measured using a Contact Length Gauge (HEIDENHAIN CT6001, HEIDENHAIN GmbH, Germany), with a specified accuracy of ±0.1μm. Photographs about the geometric parameters measurements have been added to Extended Data Fig. 1.

Method:

(9). p.5, figure caption of fig.2, l. 233: “… calculated Newtonian gravity …” I would rather say “… calculated (Newtonian) gravitation-induced torque …”

Thanks to the reviewer, we have made the necessary revisions in the manuscript.

Systematic Effects:

(10). p.6, table 1: Why does the measurement of the test mass have higher uncertainties than the source mass, although they have the same dimensions?

We appreciate the reviewer's insightful question regarding dimensional measurements. The test masses (mounted on the torsion fiber) and source masses were measured using different instruments: a Video Measuring Machine (QVP5040T, ±3 μm) for the test masses and a high-precision Coordinate Measuring Machine (Werth VideoCheck UA400, ±0.15 μm) for the source masses. This approach was necessary due to practical constraints: the Werth machine was unavailable during initial test mass measurements. By the time it became accessible, the pendulum assembly had progressed to fiber installation and sensitivity testing. Despite this difference in measurement precision, both instruments fully satisfy experimental requirements. The

dimensional uncertainty contribution to the $16f_d$ torque uncertainty is merely 0.01 a·N·m, which is negligible compared to the total uncertainty budget.

(11). p.6, 272, right column: "The remanent magnetization intensity of a silicon sample is measured using a femtotesla magnetometer …" Where did this measurement take place? In an extra (shielded) room or on the laboratory table?

We thank the reviewer for this question. The remanent magnetization of the silicon sample was measured using a high-sensitivity atomic magnetometer (noise level: $15fT/Hz^{1/2}$) within a near-zero magnetic field environment established by a multi-layer magnetic shielding infrastructure at the National Major Scientific and Technological Infrastructure of Harbin Institute of Technology. We acknowledge this facility for providing the essential low-field environment for this measurement, and this acknowledgment has been added to the revised manuscript. A reference describing the shielding facility has also been included [Z. Sun,et al., Limits of Low Magnetic Field Environments in Magnetic Shields, IEEE Trans. Ind. Electron., 68 (2021) 5385–5395.].

Results and Discussion:

(12). p.8, l.359: "…designed to access regimes relevant to quantum coherence." Can you give a number here for which masses this should happen? p.8, l.402: "Probing gravity between objects with masses below the Planck scale (mp ≈ 22 µg) is crucial for exploring its quantum nature." Do you expect quantum-gravitational effects for masses of mp? Why? How would you notice such effects in your experiment? Would both masses (source and test) need to be of that order of magnitude, or is it sufficient that one is of this mass?

Thank the reviewer for the insightful question. We have revised the relevant descriptions in our manuscript and supplemented the possible mass range for observing quantum-gravitational effects.

The Planck mass ($m_P \approx 22$ μg) is one of the most fundamental natural units in

quantum gravity research, marking the critical scale where quantum gravitational effects transition from negligible to non-negligible. It serves as both the characteristic scale for quantum gravitational effects and the target threshold for experimental verification. Therefore, this is why we aim to detect gravitational interactions at mass scales down to or below the Planck mass. According to references below (Bose, et al., PRL,119,2017,240401; Marletto, et al., PRL, vol. 119,2017,240402), to observe quantum effects induced by gravitation, the ideal scenario is that both interacting objects should be in the quantum regime, with masses much smaller than m_p. both the source mass and test mass should reach approximately the range of 10^{-15} to 10^{-13} kg. Within this mass range, gravitational interactions between micrometer-scale objects can potentially generate measurable entanglement within experimentally feasible coherence times. This target mass window is far below the Planck mass, and is the cale that our experimental efforts are advancing towards. Once again, we thank the reviewer for this valuable question.

[*] S. Bose, A. Mazumdar, G. W. Morley, H. Ulbricht,M. Toroš, M. Paternostro, A. Geraci, P. Barker, M. S.Kim, and G. Milburn, “Spin Entanglement Witness for Quantum Gravity,” Phys. Rev. Lett.,vol. 119, no. 24,p. 240401, 2017.

[*] C. Marletto and V. Vedral, “Gravitationally-induced entanglement between two massive particles is sufficient evidence of quantum effects in gravity,” Phys. Rev. Lett.,vol. 119, no. 24, p. 240402, 2017.

(13). p.8, l.375 ff: Can you say how many measurements you have taken for the evaluation of G (I could count the dots in fig.3, but it is better to read the number somewhere)?

We thank the reviewer for this suggestion. A total of 46 independent measurements were taken for the evaluation of the gravitational constant *G*. Each measurement corresponds to one data segment of 13 rotation periods (7800 s) of the source mass, from which the torque amplitude at $16\omega_d$ is extracted. We have added this information

to the revised manuscript.

(14). p.8, l. 368, right column: "At the same time, by shortening the separation from the current 654 µm to ~ 15 µm, a 10^5 improvement in signal-to-noise ratio can be obtained …" How did you calculate the number 10^5?

We thank the reviewer for this question. The 10^5 improvement in signal-to-noise ratio mentioned in the text refers to the detection of non-Newtonian Yukawa deviations, considering the combined effects of reduced separation and improved suspension fiber. The detailed derivation is provided below and added in the Supplementary Information Section 5.

(1) **Signal torque estimation (non-Newtonian Yukawa deviations)**

According to the reference (Adelberger et al., *Prog. Part. Nucl. Phys.* 62, 102 (2009)), the Yukawa torque between two parallel plates can be written as:

$$\tau_Y = 2\pi\alpha G\rho_1\rho_2\lambda^2 A\left(1-e^{-t_1/\lambda}\right)\left(1-e^{-t_2/\lambda}\right)e^{-s/\lambda}L$$

Where s is the surface separation, λ is the Yukawa range, A is the area, t_1, t_2 are the thicknesses of the two plates, and L is the lever arm.

Taking α=1, ρ=2330kg/m3 (silicon wafer), silicon wafer dimensions of $2.6\times2.6\times0.5\text{mm}^3$, lever arm L=23.7mm, and Yukawa range λ=90µm, the signal enhancement is approximately **1212 times**, as shown in Table below.

Surface separation	λ	Non-Newtonian torque
654μm	**90**μm	2.05×10^{-21} Nm
15μm	**90**μm	2.48×10^{-18} Nm

(2) **Noise estimation**

The thermal noise torque of the suspension fiber scales as (Saulson, P. R. Thermal noise in mechanical experiments. Phys. Rev. D 42(1990) 2437):

$$S_\tau^{1/2}(f) = \sqrt{\frac{4k_BTk}{2\pi Qf}}$$

where the torsional stiffness is $k = \frac{\pi R^4 S}{2l}$, with R the fiber radius, S the shear modulus, and l=0.763m is the fiber length. Replacing the 10μm tungsten fiber (Q~1800, S=161GPa) with a 3μm fused silica fiber (Q~25000, S=29.47GPa) under the same integration time, the thermal noise is reduced by approximately **97 times**, as shown in Table below.

Fiber	Diameter	Q	Torsional stiffness (Nm/rad)	Thermal noise (Nm/Hz$^{1/2}$) @26.667mHz
Tungsten	10μm	1800	2.07×10^{-10}	1.07×10^{-16}
Fused silica	3μm	25000	3.07×10^{-13}	1.10×10^{-18}

(3) Combined SNR improvement

$$\frac{SNR_{15\mu m}}{SNR_{654\mu m}} = \frac{\tau_{Y,15}}{\tau_{Y,654}} \cdot \frac{\tau_{\text{noise},W}}{\tau_{\text{noise},Q}} \approx 1212 \times 97 \approx 1.17 \times 10^5$$

This confirms the statement of ***"a 10^5 improvement in signal-to-noise ratio can be obtained."***

(4) Sensitivity estimation for measuring the Newtonian gravitational force between smaller masses.

Proposed parameters:

Material	Density (kg/m^3)	Dimensions (mm^3)	Mass (μg)	Separation (μm)	Lever arm (mm)	Newtonian gravitational torque (Nm)
Silicon	2330	2.6×2.6×0.5	**7875.4**	654	23.7	2.69×10^{-17}
Silicon	2330	0.3×0.3×0.1	**21.0**	15	23.7	1.70×10^{-20}

By reducing the silicon wafer dimensions from 2.6×2.6×0.5mm^3 to 0.3×0.3×0.1mm^3, the mass decreases from 7.8 mg to 21.0 μg, which is below the Planck mas m_P ≈22μg. The surface separation is reduced from 654 μm to 15 μm. Under this configuration, the Newtonian gravitational torque is calculated to be approximately 1.7×10^{-20}Nm using numerical integration. Using a 3μm-diameter fused silica fiber (Q=25000, shear modulus S=29.47GPa) and extending the integration time to 30 days:

$$\tau_{\text{noise}} = \frac{1.10 \times 10^{-18}}{\sqrt{30 \times 24 \times 60 \times 60}} \approx 6.84 \times 10^{-22}\text{Nm@}0.02667\text{Hz}$$

The resulting signal-to-noise ratio is:

$$SNR = \frac{1.70 \times 10^{-20}}{6.84 \times 10^{-22}} \approx 25$$

This estimation represents a reasonable extrapolation based on the current experimental parameters, providing a reference direction for future exploration of gravitational interactions below the Planck mass.

(15). Extended data figure 1: "… 0.487 mm-thick pendulum …" mm not in italic!

We thank the reviewer for the careful reading.

(16). Extended data figure 4: "Feedback voltage [V]" The unit does not go into squared brackets.

We thank the reviewer for pointing out this formatting issue.

(17). Extended data figure 6: In this figure caption you write often "k_e" with "e" non-italic, whereas throughout the manuscript it is in italic. (also "f_d" and "\delta tau_e" in this figure caption). Be consistent.

We thank the reviewer for pointing out this inconsistency.

(18). Extended data figure 8: In the figures there are spaces in the text of the legends, e.g. "vac uum"

We thank the reviewer for catching this typo.

(19). One last question: Did you try to rotate the attractor with other frequencies, in order to see if you get the same results?

Thanks to the reviewer for the insightful suggestion. Fortunately, our experimental apparatus remained operational. We increased the rotation period of the turntable from 600 s to 3600 s, which corresponds to a gravitational signal frequency of 4.444 mHz (16/3600) at the 16th harmonic of the modulation frequency (f_d). The rationale for selecting this frequency is twofold: 1) **Sensitivity consideration:** Based on the

sensitivity characteristic curve of our torsion balance, which exhibits a $1/f$-type noise profile, the noise level at this frequency is higher than that at the previously selected frequency. Nevertheless, it remains within an acceptable range. 2) **Frequency separation:** We intended to maximize the separation between the new frequency and the currently employed one ($16f_d$= 26.67 mHz), thereby enabling the observation of any potential frequency-dependent phenomena.

We conducted 15 sets of measurements with the surface spacing of 0.654mm between the test mass and source mass, each involving 13 full rotations of the turntable and taking 13 hours. Following the signal extraction method described in Supplementary Information Section 1, we obtained a gravitational signal of τ_2 = 11.41(46) aNm at the $16f_d$ (4.444 mHz) frequency, where the uncertainties represent 1σ statistical uncertainties only. Within the 1σ uncertainty range, this result is consistent with our previously measured gravitational signal τ_1=11.16(27) aNm at $16f_d$ (26.667mHz). The increased noise at the new frequency is primarily due to the slower experimental frequency, which leads to greater *1/f* noise. The results indicating that no frequency-dependent phenomenon was observed. We have incorporated these findings into the main text and the Extended Data Figure 9. We sincerely appreciate the reviewer's valuable suggestions.

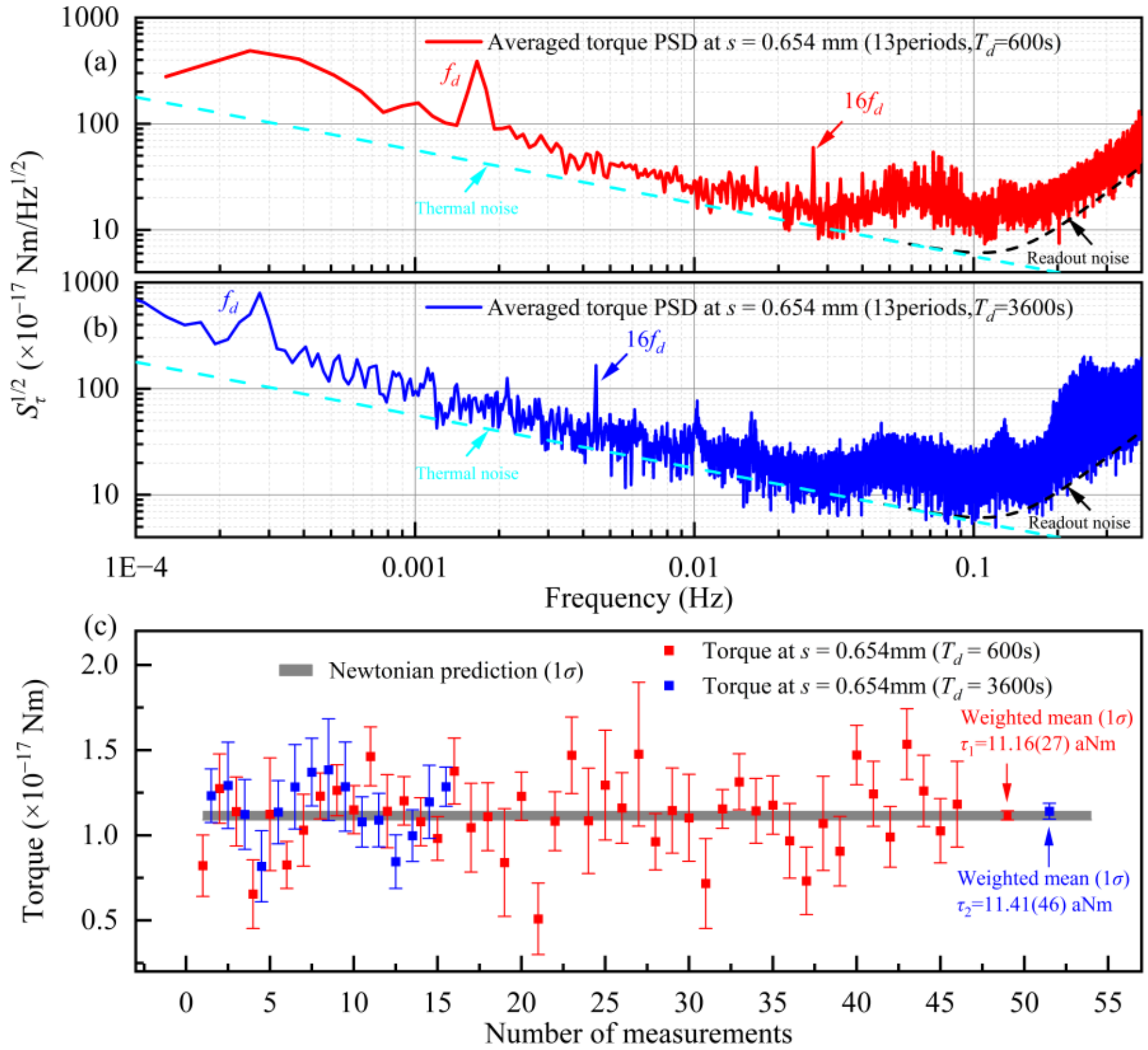

Extended Data Figure 9. Comparison of measured gravitational torques for the source mass disk rotating at two different periods (600s vs 3600s) at s = 0.654 mm. (a) & (b) Average torque power spectral densities (PSDs) of the torsion balance from multiple independent measurement segments (each containing 13 rotation periods) at s = 0.654 mm for rotation periods of 600 s and 3600 s, respectively. Distinct signals were observed at frequency 16fd (26.667 mHz and 4.444 mHz, respectively). (c) Torque amplitudes at 16fd frequency extracted from data segments using the time-domain orthogonal decomposition method (Supplementary Information Section 1). Each point represents the average torque over 13 rotation periods. At the signal frequency of 26.667 mHz, 46 measurements yielded a weighted mean τ_1 = 11.16(27) aNm, while at 4.444 mHz, 15 measurements yielded τ_2 = 11.41(46) aNm. The uncertainties represent 1σ statistical uncertainties only. The gray band indicates the theoretical Newtonian torque of 11.17(26) aNm at this separation distance. The elevated noise level at 4.444 mHz compared to 26.667 mHz primarily results from increased *1/f* noise characteristics of the torsion pendulum at lower frequencies. All measurements remain consistent within 1σ uncertainties, confirming the frequency independence of the gravitational measurement results.

Recommendation for the editor

This article describes a very interesting experiment and is surely interesting to a

broad physics community. So, it is worth its publication, because the measurement of the gravitational constant is still a challenging task and is worth to be investigated on a broad frequency scale, as well as for small masses. A shortcoming is clearly the abstract. This definitely should be re-written. If the authors consider my comments and questions, then I can recommend a publication.

We are grateful for the reviewer's insightful suggestions and the affirmation our work. The abstract has been thoroughly revised to incorporate the feedback. Regarding other concerns, we have provided specific responses to each issue, and for several of these, we conducted additional experimental work.

Responses to Reviewer #3: (marked as blue)

Reviewer #3 (Remarks to the Author):

The authors report the measurement of the Newtonian gravitational force from a 16-spoked silicon source mass using a ~7 mg test mass suspended as a torsional pendulum. They infer the Newtonian gravitational constant G --- the least precisely known of all the fundamental constants --- from their measurement, a result consistent with the latest CODATA value.

The significance of the experiment is that it is the smallest test mass used in the measurement of Newtonian gravity yet. Although the authors claim, somewhat loosely, that this has ramifications for tests of quantum gravity etc, these should be taken with a grain of salt. However, in Extended Data Figure 9, they contextualize their result in terms of limits on Yukawa-type modifications of gravity. Although not competent in an absolute sense, the combination of this low mass for the test mass makes such a test scientifically interesting.

There are however some comments/issues that would significantly aid the manuscript:

(1). The most serious deficiency of the manuscript is as follows: Fig. 2(f) is the main measurement result, purporting to show the gravitational torque signal at 16f. But from looking at the spectrum, it is impossible to see whether the arrow the authors point to (at 16f) is some noise spike coming from the variance of the spectrum estimate, or an actual signal! The authors need to do some very detailed statistical analysis --- starting with studying the histogram of the spectral estimates --- to show that this spike is indeed a signal. Details of such analysis needs to be provided in the supplementary information as well, perhaps with the data and code used to analyze it.

We appreciate the reviewer's valuable suggestions. The signal in the power spectrum density of Figure 2(f) is not readily discernible for two primary reasons. First, while our experiment employed two 7.6-mg small objects as test mass and source mass, the resulting gravitational signal is inherently weaker. Second, to limit the temperature

rising of the turntable in vacuum, we implemented an intermittent operation mode: 13 full revolutions (7,800 s) of turntable rotation followed by a 3,600 s pause before resumption. Consequently, Figure 2(f) shows the power spectrum analysis from a single 7,800-second dataset, and the limited integration time explains the insufficiently prominent gravitational signal at $16f_d$.

In response to the reviewer's suggestion, we have enhanced the visibility of the $16f_d$ gravitational signal at s=0.654 mm by computing the averaged power spectrum from six independent 7,800 s data segments, which effectively suppresses random fluctuations. Additionally, as shown in the revised Figure 2(e), we expanded the plot area to clearly reveal a distinct gravitational signal at $16f_d$. Further contrast enhancement would require extending the measurement time. **For comparison,** an identical measurement was performed at a larger separation (s ≈ 3 mm), where the Newtonian gravitational torque at $16f_d$ is theoretically expected to fall below the torsion balance noise floor and thus remain undetectable in the power spectrum. The measurement confirms this expectation: a prominent signal appears only at the drive fundamental frequency f_d, with a magnitude consistent with the close-separation result, while no discernible signal is present at $16f_d$. This control test further corroborates the reliability of the close-separation measurements. In the frequency band above 30 mHz, some random peaks are likely attributable to membrane frame vibrations induced by the rotary stage and the ground, which represents an area requiring improvement in subsequent experiments.

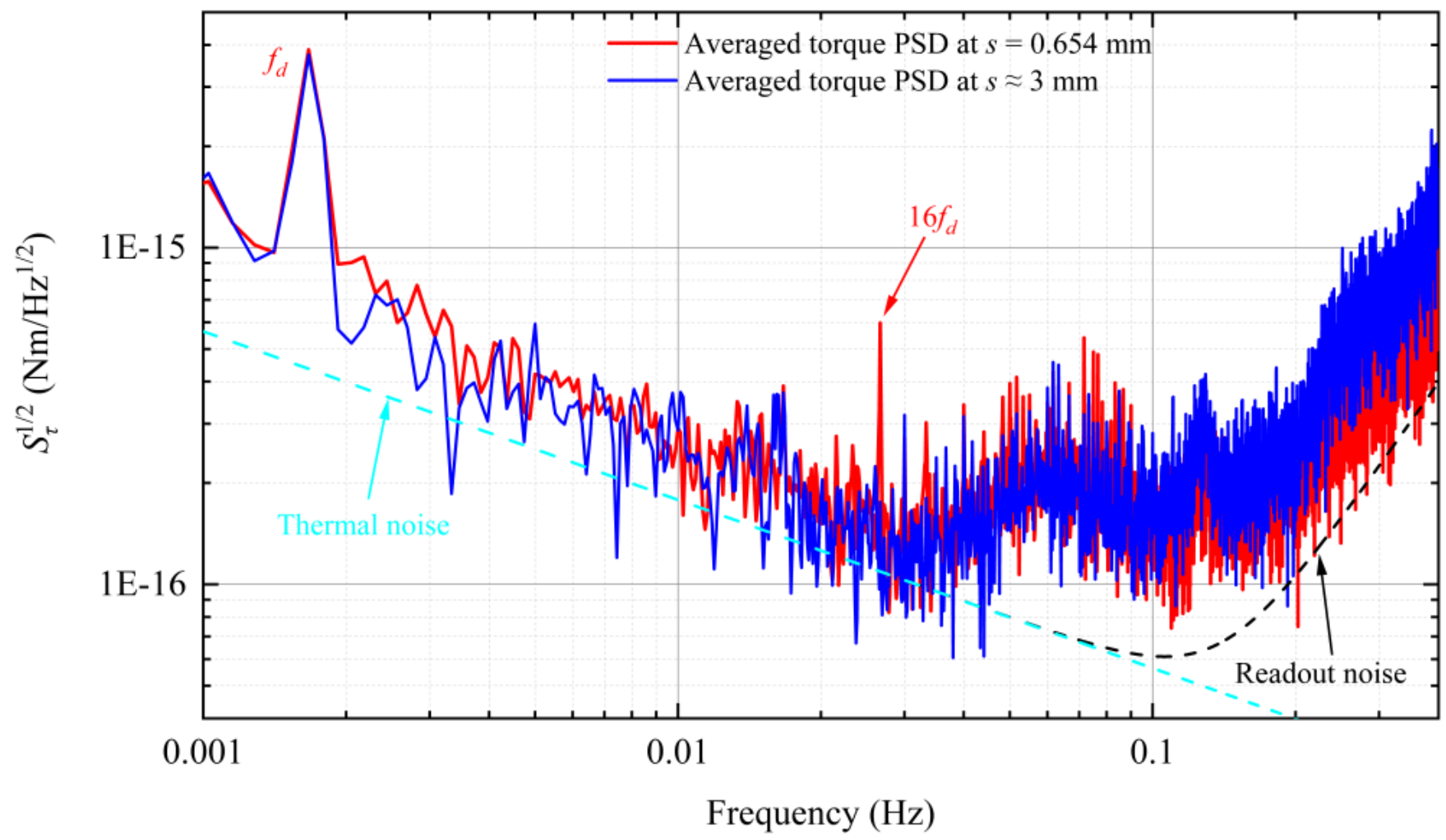

Figure 2(e): Comparison of averaged torque PSD at separations of 0.654 mm and 3 mm. At s = 0.654 mm, a distinct gravitational signal is clearly observed at $16f_d$. Conversely, at the larger separation of $s \approx 3$ mm, the Newtonian gravitational torque at $16f_d$ is theoretically expected to fall below the torsion balance noise floor, rendering it undetectable in the power spectrum. The experimental results confirm this theoretical expectation. This control test further corroborates the reliability of the close-separation measurements. In the frequency band above 30 mHz, some random peaks are likely attributable to membrane frame vibrations induced by the rotary stage and the ground, which represents an area requiring improvement in subsequent experiments.

(2).The authors actually claim that the experiment evidences gravitational attraction between two 7.6 mg masses. But this would only be true if the source mass contribution is dominated by each of its paddles, and very little of the spokes and central mass. Since details of the geometric integration to estimate the Newtonian force is insufficiently detailed, it is unclear whether this claim is true.

We thank the reviewer for this important concern. In our theoretical calculation of the Newtonian torque presented in the manuscript, we have already included the contributions from both the 16 source masses and their supporting spokes, using the measured geometries of all components. At the closest separation of s=0.654mm, the calculated $16\omega_d$ Newtonian torque from the spokes alone is 0.53aNm, which corresponds to about 4.7% of the total calculated Newtonian torque of $11.17(26)\times10^{-18}$Nm. This quantitative evaluation confirms that the gravitational signal is dominated by the 16 source masses, while the contribution from the spokes and

central structure is small. We have added this result to the caption of Fig. 2(b) to clarify that the theoretical calculation includes all geometric components.

(3).Indeed, the manuscript seems to suggest that the 16f signal is dominated by other effects, with a sub-dominant contribution from Newtonian gravity, which already refutes the claim that the 16f signal can only arise from gravitational coupling; any synchronous effect at f could have a 16f component.

Thanks to the reviewer. We sincerely apologize for any misunderstanding that may have been caused by imprecise expressions in our manuscript. The objective of this experiment was to precisely measure the gravitational force between two silicon wafers, each with a mass of 7.6 mg (measuring the gravitational signal at 16*f* din this experiment). To enhance the precision of gravitational measurements, the entire experimental design aimed to minimize or eliminate interference from other effects at 16*f*, thereby highlighting the predominant contribution of Newtonian gravitational force at 16*f*.

First, substantial effort is devoted to investigating the impact of systematic errors affecting the 16*f* gravitational signal, including electrostatic forces, magnetic fields, temperature variations, and the geometric dimensions, positions, and densities of both test and source masses. Each of these systematic errors requires rigorous analysis or dedicated modulation experiments to quantify their individual effects. This process is both extremely challenging and labor-intensive, yet it remains absolutely essential for achieving absolute measurements.

Additionally, we reduce statistical uncertainties through the following approaches: 1) Placing the torsion pendulum in a high-vacuum chamber to minimize molecular Brownian motion effects. 2) Implementing mass rotation modulation to shift the measurement frequency from f to 16*f*, which effectively reduces 1/*f* noise in the torsion fiber while suppressing potential signal leakage from the fundamental frequency *f* that could interfere with the gravitational signal at 16*f*. 3) Employing multiple measurement

strategies, including increased integration time and other statistical enhancement methods to further reduce statistical errors.

Ultimately, through uncertainty evaluation, we realized the relative uncertainty of gravitational measurements at 16*f* to approximately 5% (1σ), that is to say, in this experiment, the gravitational force is the dominant force.

(4).Related: the extended data contains very many measurements of various extraneous causes that might affect the experiment. But the effect of these causes are much less clear from these plots, or the brief discussion in the main text. As simple examples:

(a) if the source mass is rotated at a frequency f, any imbalance in the spokes can produce an acceleration at 16f, which, through the mechanical contact to the test mass, can produce an acceleration (and therefore torque) signal at 16f.

We sincerely thank the reviewer for this insightful question. This critical issue was thoroughly evaluated during the experimental design phase.

Firstly, throughout the experimental process, the source-mass-to-membrane separation is maintained at 0.268 mm, while the pendulum-membrane gap remains ≥ 0.336 mm. This substantial separation ensures complete prevention of direct contact between the components.

Secondly, we enhanced structural rigidity through careful design considerations. The disk-shaped source mass, resembling a "Ferris wheel" configuration, was specifically designed to maximize structural stability. Using SolidWorks software, we conducted comprehensive finite element analysis to simulate and evaluate structural deformation under gravity loading. The results indicated maximum deformations of less than 0.05 μm for the disk structure and less than 0.5 μm for the torsion balance (see below Fig. R1). These deformation values are comparable to the respective geometric dimensional tolerances, resulting in torque errors of less than 0.01 aN·m, which can be negligible.

In addition, we systematically examined the structural integrity of the disk-shaped

source mass from multiple orientations during rotation using a Video Measuring Machine (Model: QVP5040T; accuracy: ±3 μm). Within the instrument's measurement precision of ±3 μm, no resolvable deformation was observed (Fig. R2). Furthermore, the turntable operates at an extremely slow rotation speed of approximately 600 seconds per revolution, ensuring exceptional stability throughout the rotation process. Even in the hypothetical scenario where one source mass becomes unbalanced, its primary effect would manifest at the fundamental frequency f_d, with significantly reduced influence at $16f_d$. Therefore, the reviewer's concern has a negligible impact on this experiment.

Once again, we appreciate the reviewer's rigorous and thought-provoking question that has helped us further consider our experimental design.

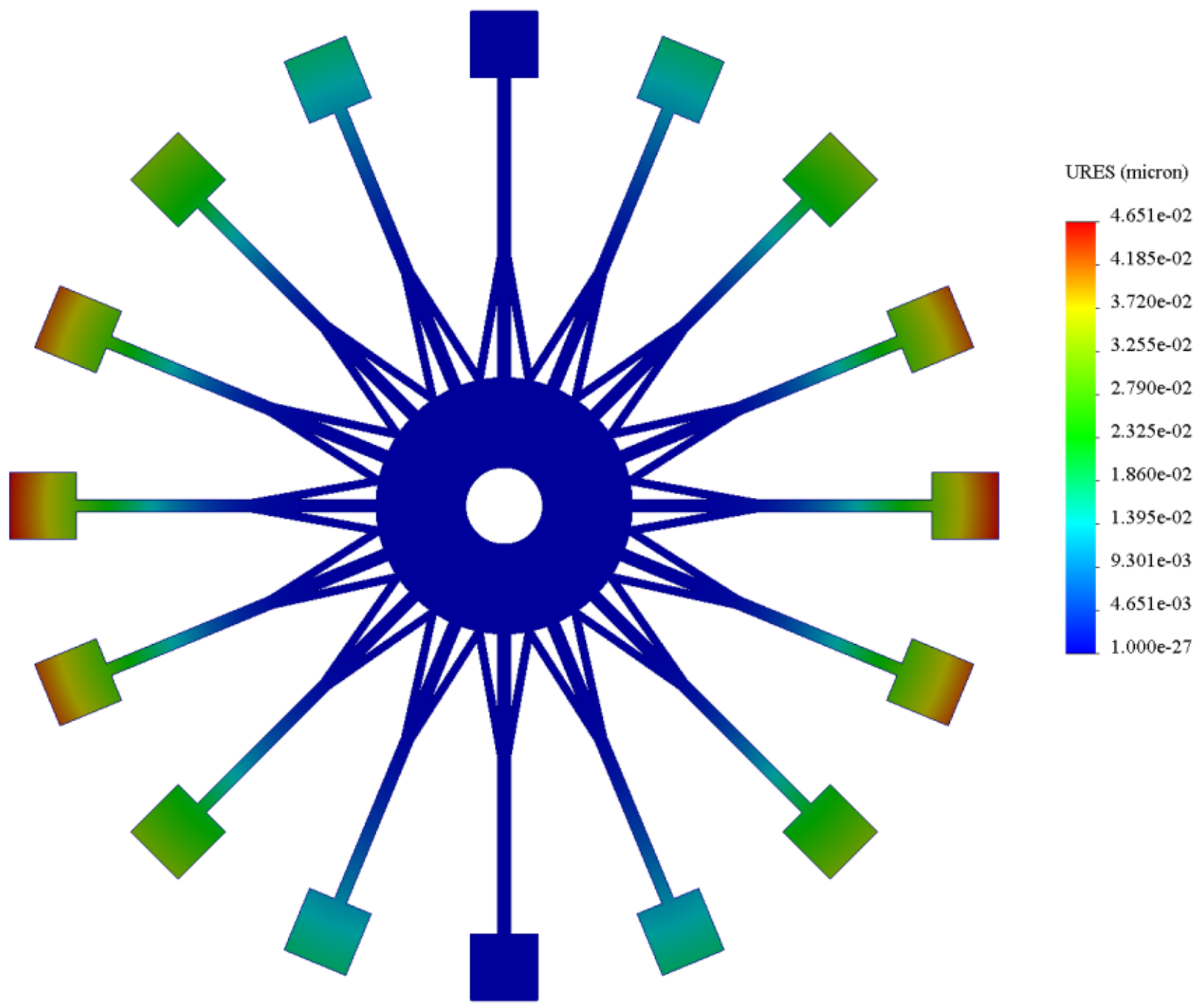

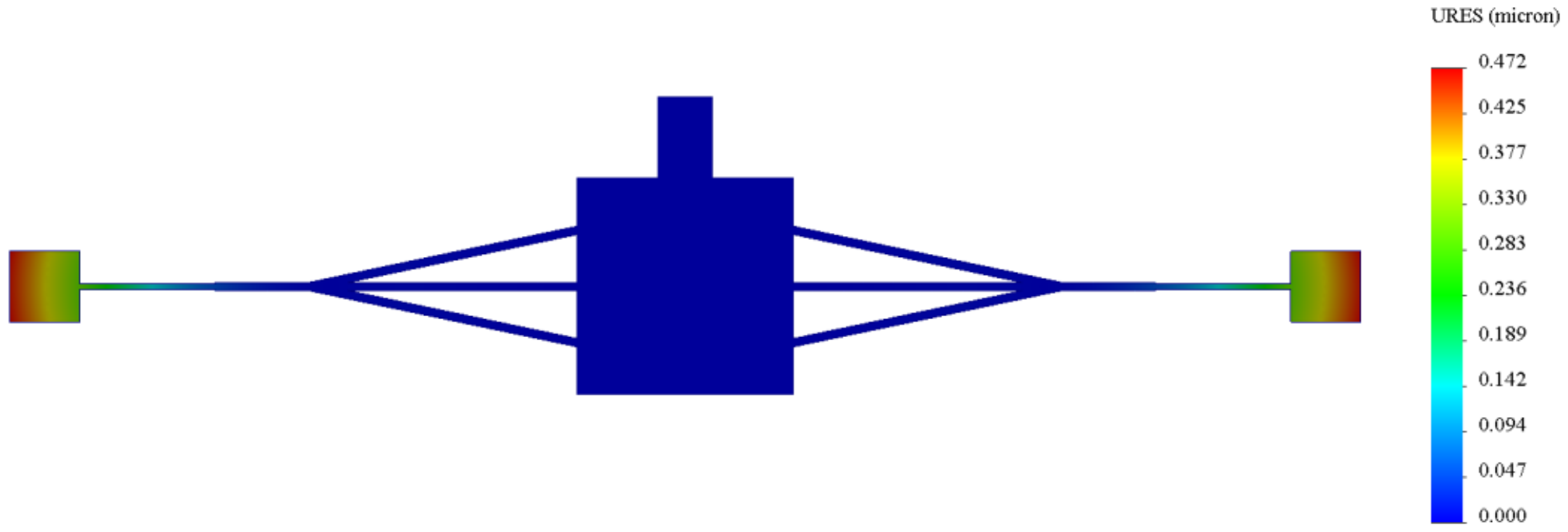

Fig.R1. Simulation results of the deformation of the source mass disk and the torsion pendulum. The results indicated maximum deformations of less than 0.05 μm for the disk structure (upper) and less than 0.5 μm for the torsion pendulum (lower).

[editorial note: figure with people present has been redacted]

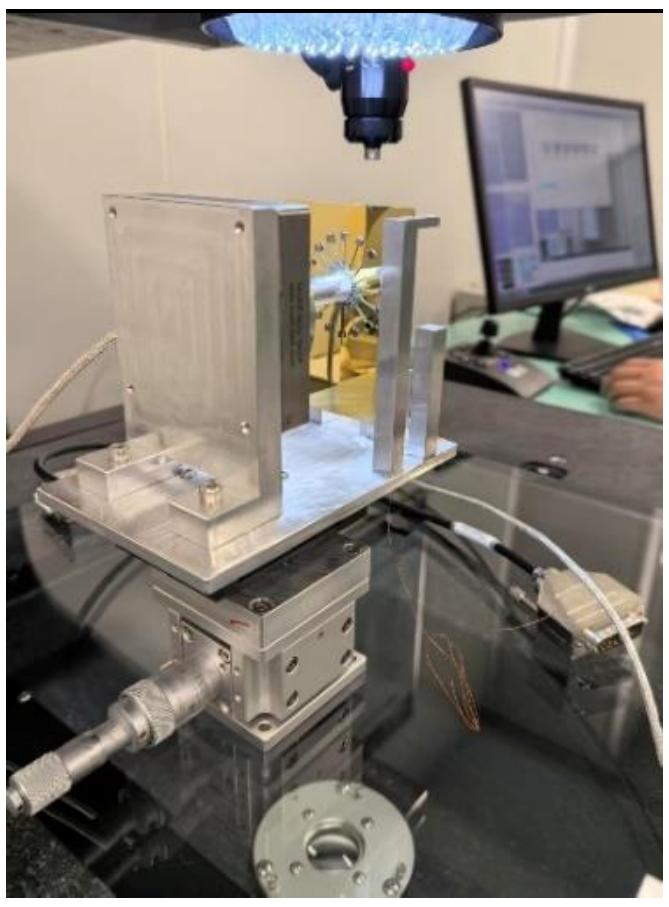

Fig.R2. Photograph of checking the structural integrity of the disk-shaped source mass during rotating under a Video Measuring Machine.

(b) the magnetic field estimates seem to focus on uniform static fields; what about rotating fields from the source mass' motor windings?

Thank the reviewer for this insightful comment. We agree that rotating magnetic fields generated by motor windings could, in principle, introduce synchronous interference, and we have carefully examined this possibility.

First, our rotary stage is driven by a piezoelectric ceramic motor (Multi-Field Technology RS65.Lab.HV) instead of a conventional electromagnetic motor, significantly reducing dynamic magnetic field generation. Despite this precaution, we measured magnetic field variations near the rotary stage under both stationary and

rotating conditions using a three-axis magnetometer (Bartington Mag-13; noise level: < 6 pT RMS/√Hz at 1 Hz), as shown in Fig. R3. The power spectral density analysis reveals no discernible differences in magnetic field components along all three axes between rotating and stationary conditions (Fig. R4). Notably, at the signal frequency $16f_d$, no additional magnetic signal emerges during turntable rotation.

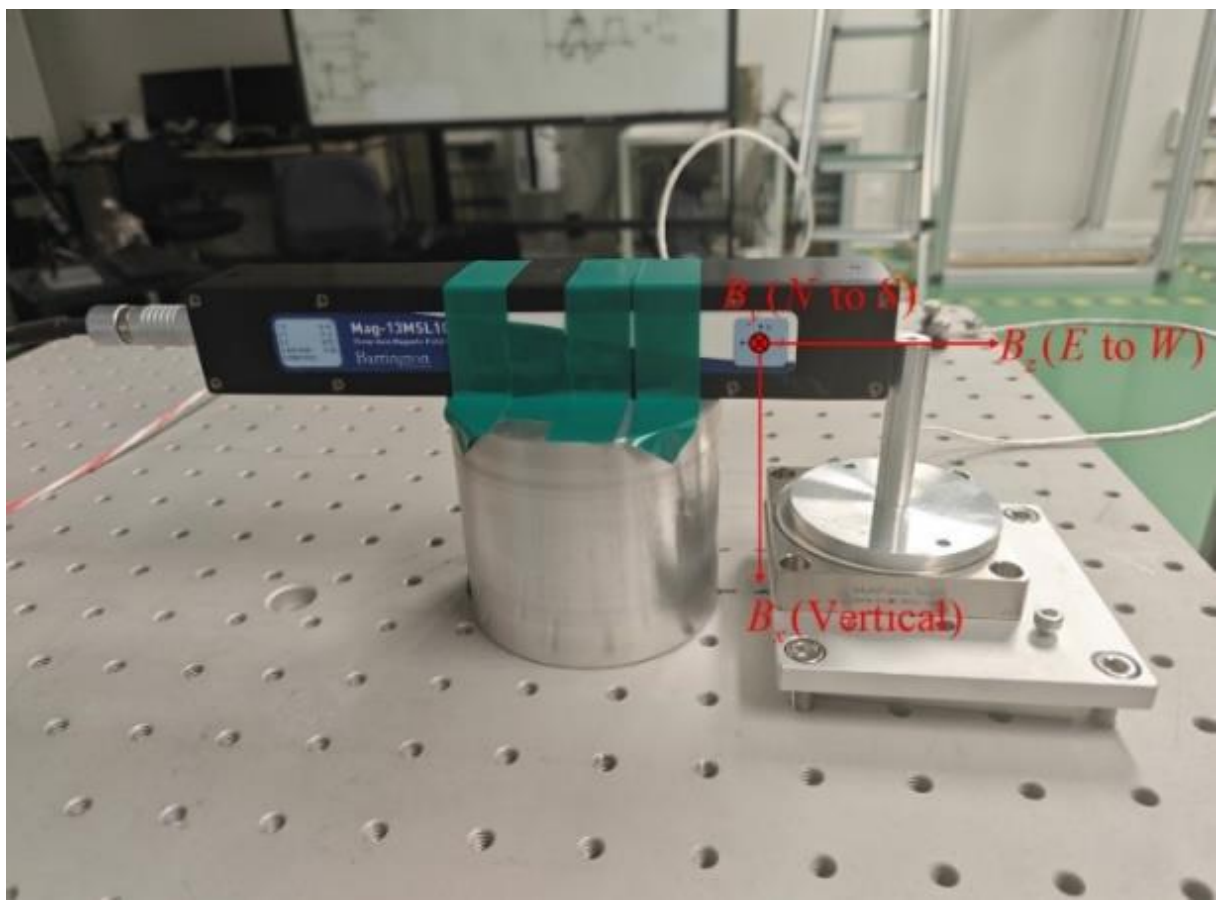

Fig. R3. Three-axis fluxgate magnetometer positioned for measuring magnetic field variations near the rotary stage.

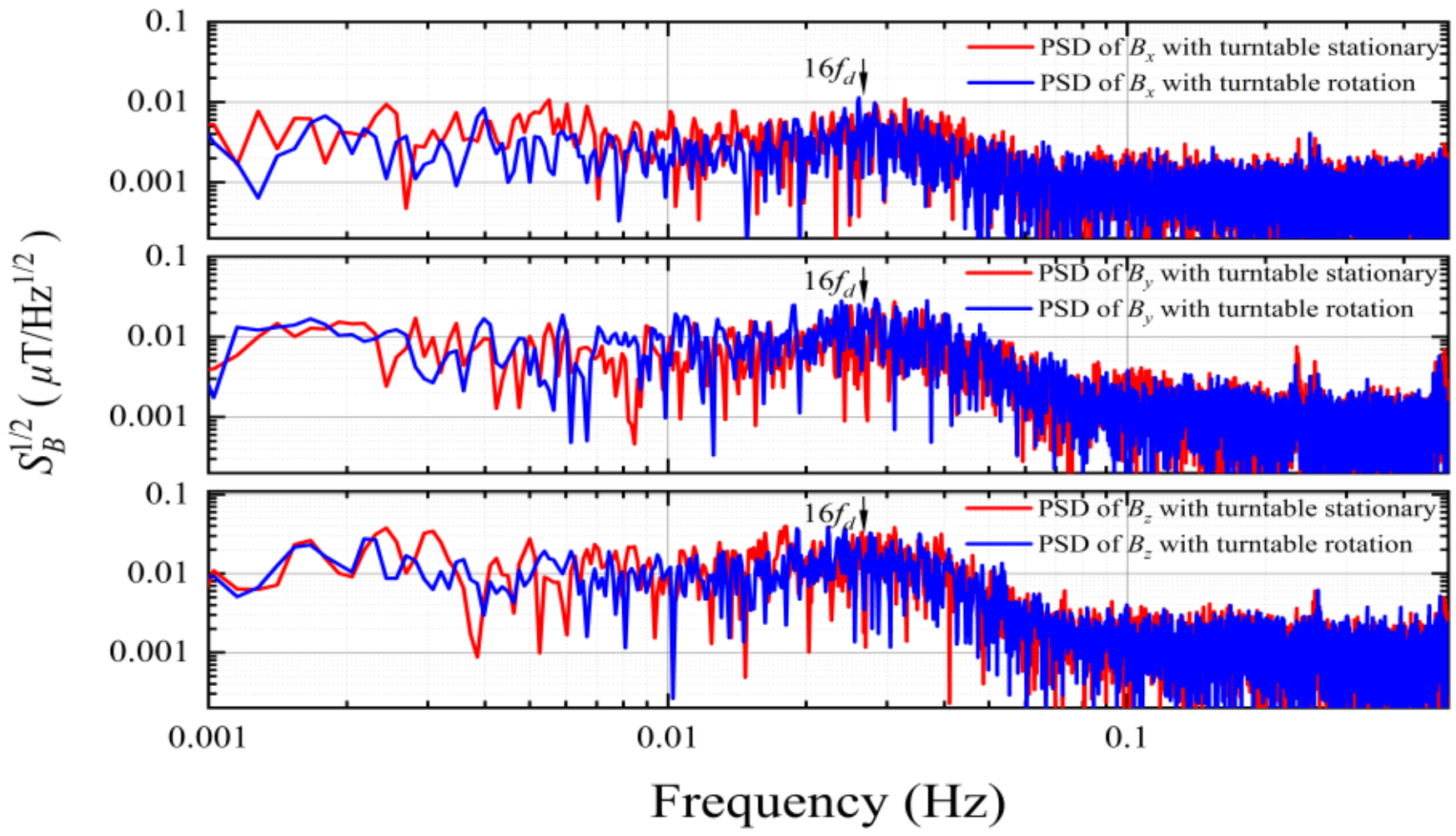

Fig. R4. Magnetic field power spectral densities along orthogonal axes under stationary (red) versus rotating (blue) conditions, demonstrating negligible differences at the signal frequency $16f_d$.

Even so, following the reviewer's suggestion, we evaluated alternating magnetic field effects using the procedure illustrated in Extended Data Figure 7(a)-7(d). (a)Experimental configuration. The torsion balance responds exclusively to

vertical torque components, making horizontal magnetic field variations the primary consideration. To characterize the magnetic torque response, a pair of horizontally orthogonal coils was implemented to generate controlled alternating magnetic fields for systematic calibration. (b) Torque response characterization. The torsion balance's torque response to alternating magnetic fields was measured at a modulation frequency of 26.667 mHz ($16f_d$). Linear regression analysis yielded sensitivity coefficients for the x- and y-axes: $\eta_x = \partial\tau/\partial Bx = 9.1(1)\times10^{-17}$ Nm/μT,$\eta_y=\partial\tau/\partial By=3.71(1)\times10^{-17}$ Nm/μT. (c) Magnetic field monitoring. Magnetic field along laboratory x- and y-directions were continuously monitored using a Bartington Mag-13 magnetometer positioned adjacent to the torsion balance. The measurement duration was approximately 20,000 seconds, with the turntable operating in rotation mode throughout the data acquisition period. (d) Spectral analysis and torque fluctuation calculation. Power spectral density (PSD) analysis of the magnetic field data revealed fluctuations. Applying the same orthogonal decomposition methodology used for gravitational signal analysis, the magnetic field fluctuation amplitudes at the $16fd$ frequency were determined as:$\delta Bx \leq 0.00017$ μT,$\delta By \leq 0.00023$ μT. Using the relationship $\delta\tau = \eta\cdot\delta B$, the corresponding torque fluctuations at $16fd$ were calculated:$\delta\tau x \leq 1.5\times10^{-20}$Nm,$\delta\tau y \leq 9\times10^{-21}$Nm. The combined torque fluctuation is given by:$\delta\tau B\perp=\sqrt{(\delta\tau x^2 + \delta\tau y^2)}\leq0.02$aNm. This comprehensive analysis demonstrates the systematic characterization of the alternating magnetic field effect to the torsion balance.

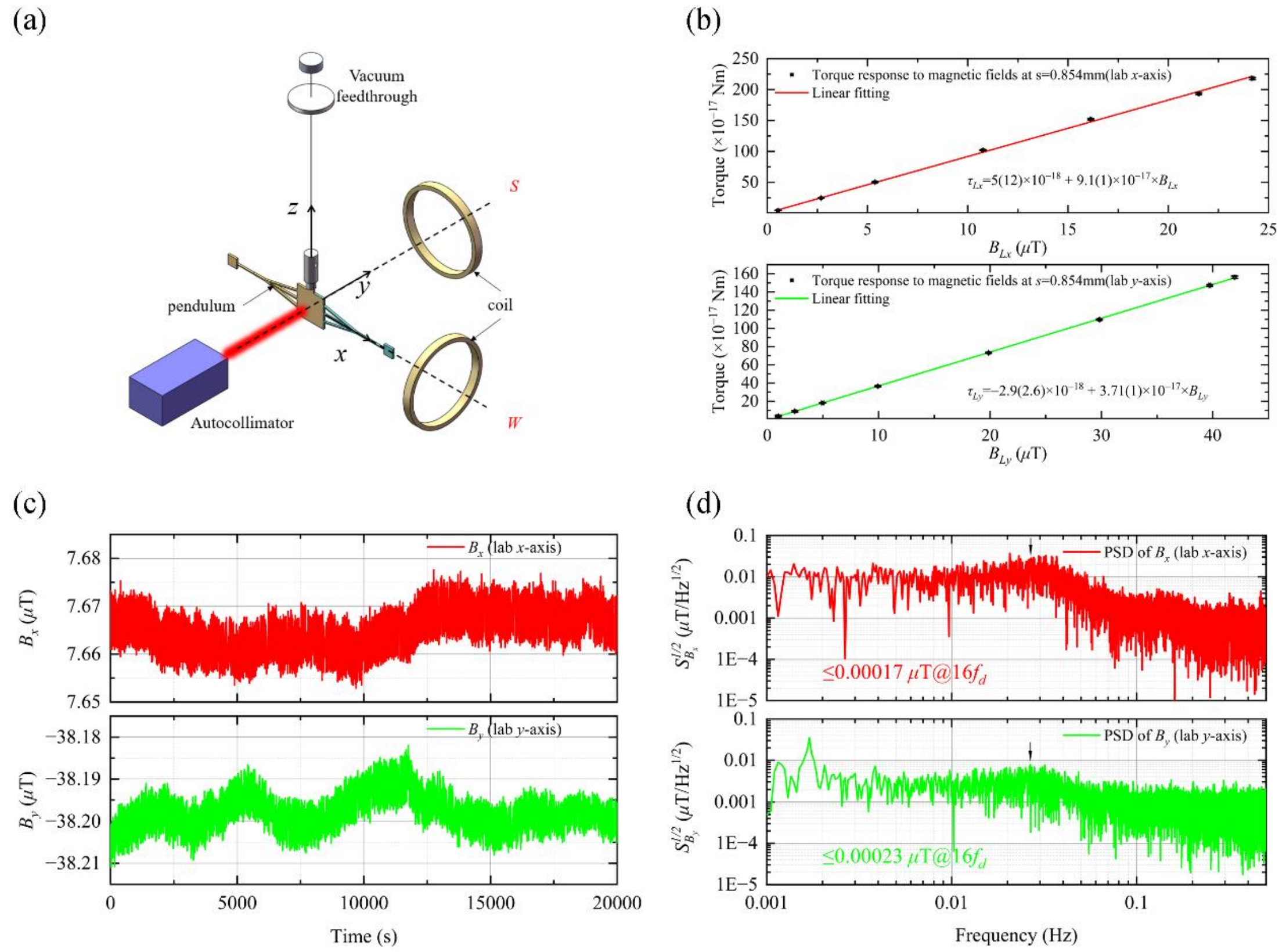

Extended Data Figure 7. Analysis of torsion balance response to alternating magnetic fields.

(c) What about forces due to residual gas pressure? Rotation of the source causes gas motion at all harmonics of the rotation frequency, which could affect the pendulum and/or (above point) membrane.

We thank the reviewer for the question. The impact of this issue can be negligible, and the relevant analysis is as follows.

First, consider the squeeze-film damping force fluctuations between two closely spaced opposing parallel plates (corresponding to the torsion pendulum and the membrane, as shown in Fig. 1:(a) Schematic view of the experimental setup in the manuscript), whose power spectral expression is given below [Mao *et al.*, Classical and Quantum Gravity, 40(2023)075015, Cavalleri *et al.*, Physics Review Letters. 103(2009)140601]:

$$S_F = 8kTPab\left[\left(\frac{2m}{\pi kT}\right)^{\frac{1}{2}} + \frac{\tau}{d}\right], (\mathrm{N}^2/\mathrm{Hz})$$

Where k=1.38×10^{-23} J/K is the Boltzmann constant, $T \approx 300\mathrm{K}$ is the ambient

temperature. In experiment, the vacuum chamber is maintained by an ion pump with a vacuum pressure of $P \approx 10^{-5} Pa$. *a* and *b* are the side lengths of the test/source mass ($a = b = 2.6$ mm), *m* is the mass of a single molecule (in a high-vacuum chamber, water molecules are typically the dominant species [Crank J. The mathematics of diffusion [M]. Oxford university press, 1979], here we take $m = 2.99 \times 10^{-26}$ kg), and *d* (=0.25mm) is the gap distance between the two plates. $\tau \approx 5.7 \times 10^{-6} s$ is the molecular escape time, obtained from Monte Carlo simulation under the current conditions. The calculated noise power spectral density of the squeeze-film damping force between the two plates is $S_F^{1/2\,'} \approx 2.4 \times 10^{-16} \mathrm{N}/\sqrt{\mathrm{Hz}}$. Substituting the effective lever arm of the torsion pendulum L_{eq} = 23.694(3)mm, the torque fluctuation noise is obtained as $S_{torque}^{1/2\,'} \approx 5.7 \times 10^{-18} \mathrm{Nm}/\sqrt{\mathrm{Hz}}$, which is far below the current thermal noise floor of the torsion balance ($\sim 1 \times 10^{-16} \mathrm{Nm}/\sqrt{\mathrm{Hz}} @ 16.7 mHz$, dominated by the structural dissipation of the torsion fiber) and can therefore be neglected.

Secondly, we consider the effect of the source mass disk rotation on gas molecule motion. This analysis is conducted from three aspects: (1)Due to the membrane and shielding box enclosing the source mass and turntable, the direct pathway for the source mass motion to affect the torsion balance through gas is eliminated. (2)Compared to the speed of gas molecules (500-600 m/s), the equivalent motion speed of the source mass (0.0002 m/s) has a negligible effect on the molecular collision rate. (3)The primary potential impact occurs when individual source masses alternately face the membrane, which can alter the collision frequency of molecules with the membrane, leading to periodic pressure variations that affect membrane motion.

We employ the Monte Carlo method for molecular collision simulation. As illustrated in the figure below, particles are generated at random positions within a $12\times12\times0.25$ mm^3 rectangular prism, with initial velocities assigned according to the Boltzmann distribution. The particle count satisfies the ideal gas law pV=NRT, and particle trajectories are subsequently tracked. When particles collide with the

membrane or source mass, they undergo diffuse reflection (shown as red and green particles), with their impulse, position, and time recorded. The simulation continues until particles reach the rectangular prism boundary (shown as yellow particles), at which point they are considered to have escaped.

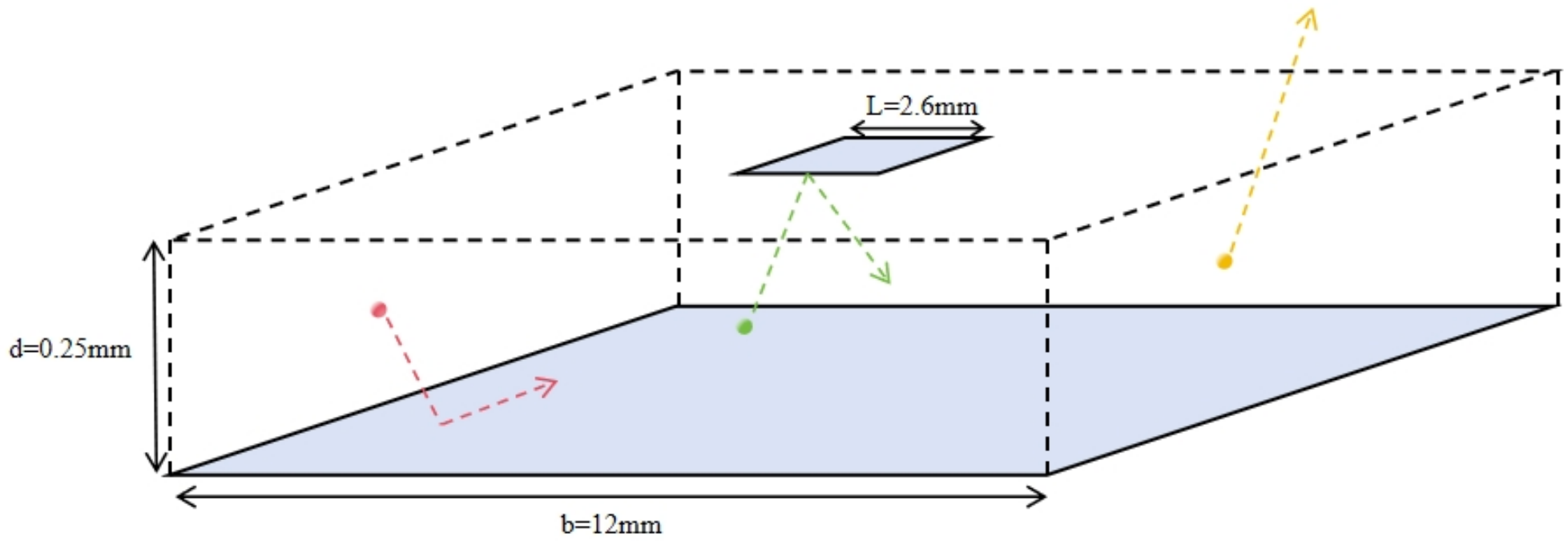

Fig. R5. Monte Carlo simulation of molecular collision process between two plates.

Through the simulation, we can provide both force fluctuation noise and derive pressure variations. Simultaneously, we can collect molecular collision counts to estimate the force acting on the shielding membrane surface during the simulation. For the central region of the shielding membrane with area $A = 2.6\times2.6$ mm², the simulation yields molecular collision counts of $n_1 = 2.1\times10^6$ without the source mass and $n_2 = 19\times10^6$ with the source mass present. As pressure is proportional to molecular collision counts, we conducted a conservative maximum estimation. During the source mass disk rotation, we assumed that the pressure difference on the membrane surface, caused by residual gas molecules in the vacuum chamber ($P \approx 1 \times 10^{-5}$Pa), reaches a value of $\Delta F = \frac{n_2 - n_1}{n_1} PA \approx 5.44 \times 10^{-10}$N.

Finally, once the pressure difference on the membrane surface caused by the rotating source mass disk is determined, SolidWorks simulation yields deformation of the membrane or frame system, the maximum is approximately 2.6×10^{-4} nm. This value is completely negligible compared to the membrane vibration size measured by using an interferometer (<0.324 nm) as shown in Extended Data Fig. 6.

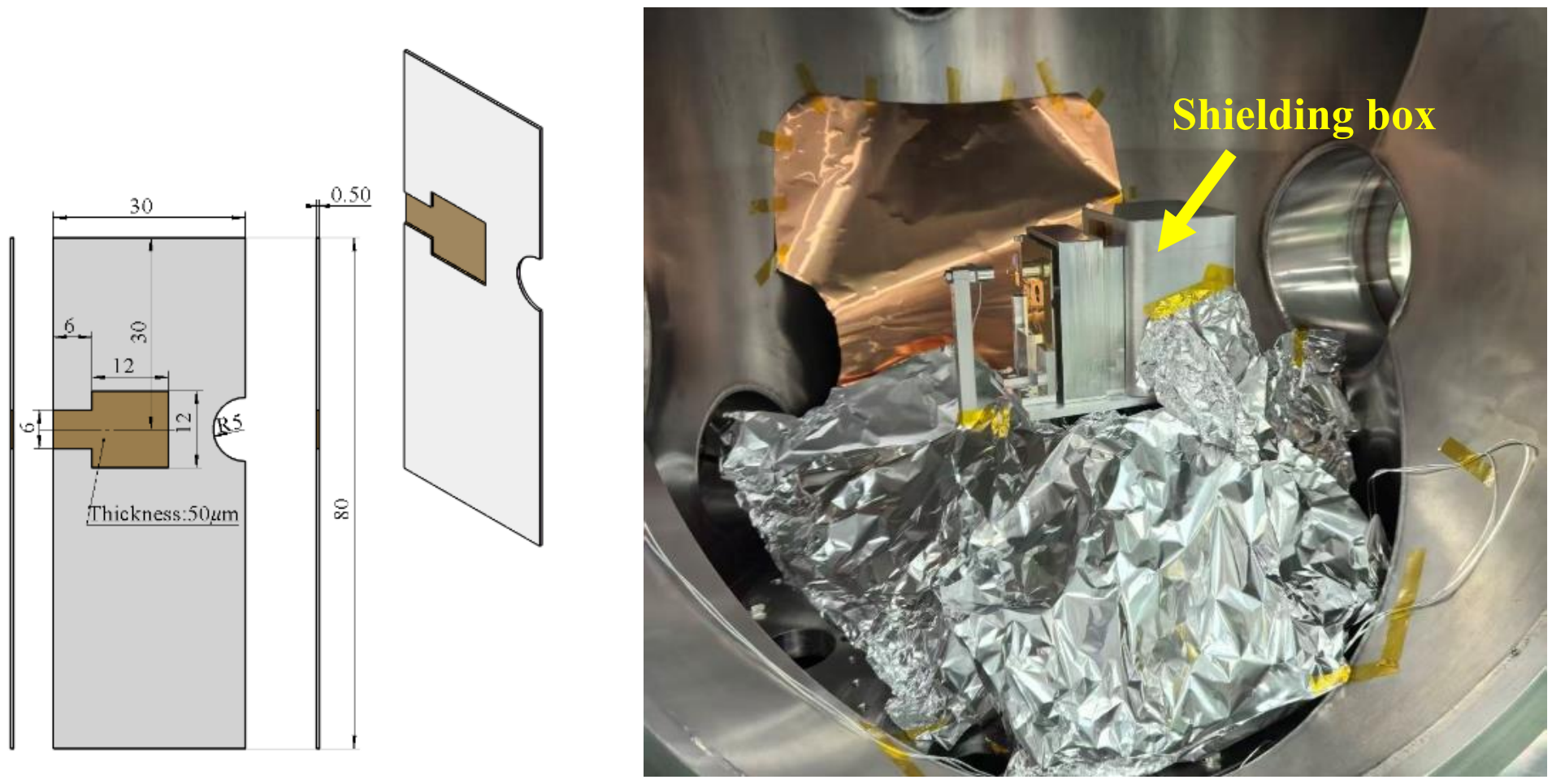

Fig. R6. (Left) Design drawing of the shielding membrane and frame, which consists of a 0.5 mm thick silicon wafer overall, with a thickness of 0.05 mm in the area directly opposite between the test and source masses (approximately 12×12 mm^2). (Right) Shielding box enclosing the motor and source mass to reduce disturbances such as electrostatic and air turbulence.

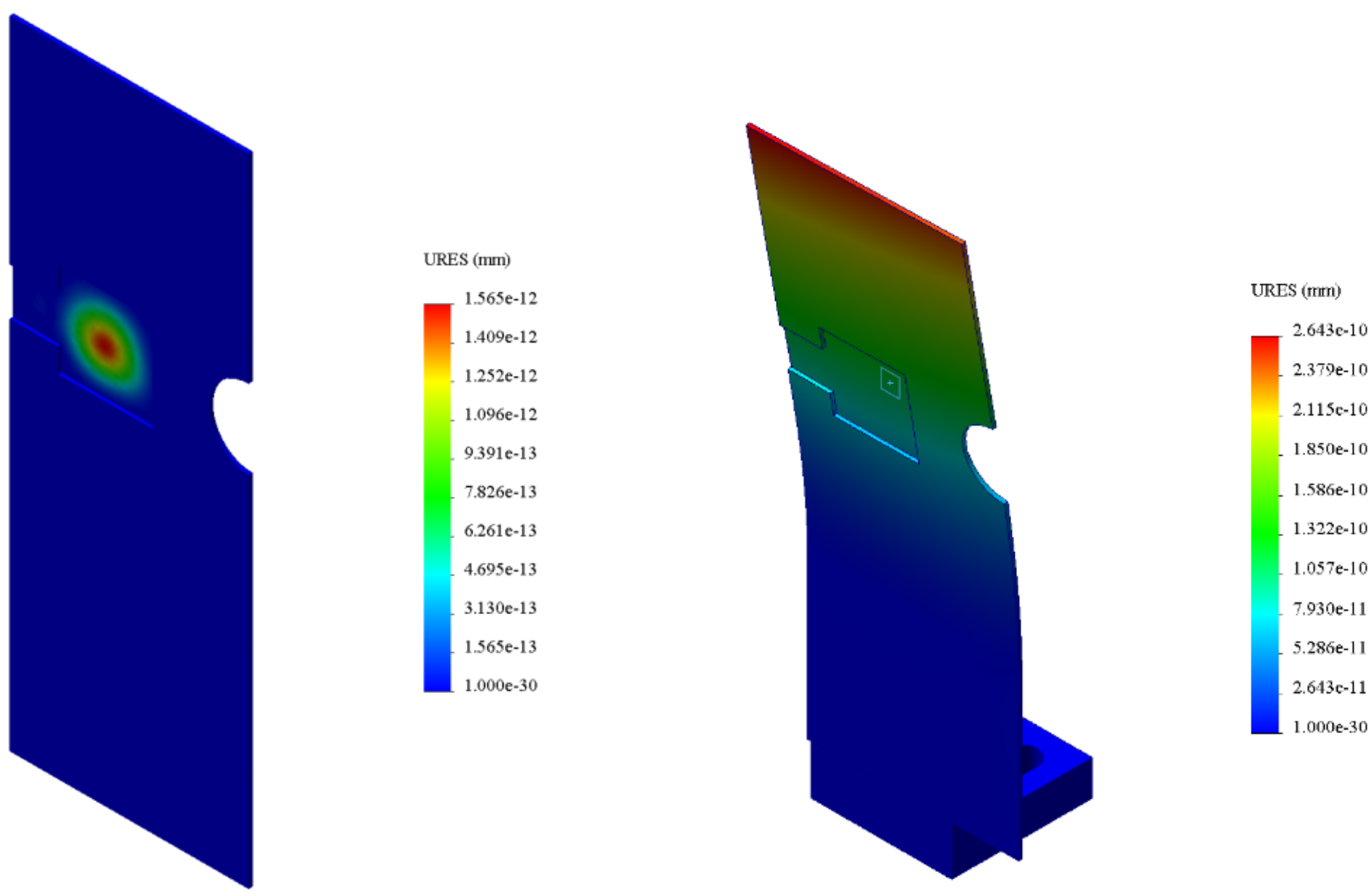

Fig.R7. Force-induced deformation of the membrane and frame simulated using SolidWorks software. (Left) Frame fixed, with deformation only simulated for the membrane region. (Right) Bottom of the frame fixed, with deformation simulated for both the membrane region and the entire frame, the maximum deformation is approximately 2.6×10^{-4} nm, it can be completely disregarded.

(d) what about potential 16f fluctuations in the capacitive bias?

We thank the reviewer for the valuable comments. The capacitor bias voltage

fluctuations is most likely to be affected by the rotary stage motion through the signal crosstalk. To minimize signal crosstalk, the voltage module that controlling the torsion balance and the turntable were operated on separate computers, each connected to independent ground lines.

Furthermore, to comprehensively evaluate the impact of turntable rotation on the entire experiment—including effects on bias voltage—we conducted additional comparison tests with a larger separation (s ≈ 3 mm), where the Newtonian gravitational torque at $16f_d$ is theoretically expected to fall below the torsion balance noise floor and thus remain undetectable in the power spectrum. If there are other possible interferences at interested frequency, then corresponding signal peaks will be observed.

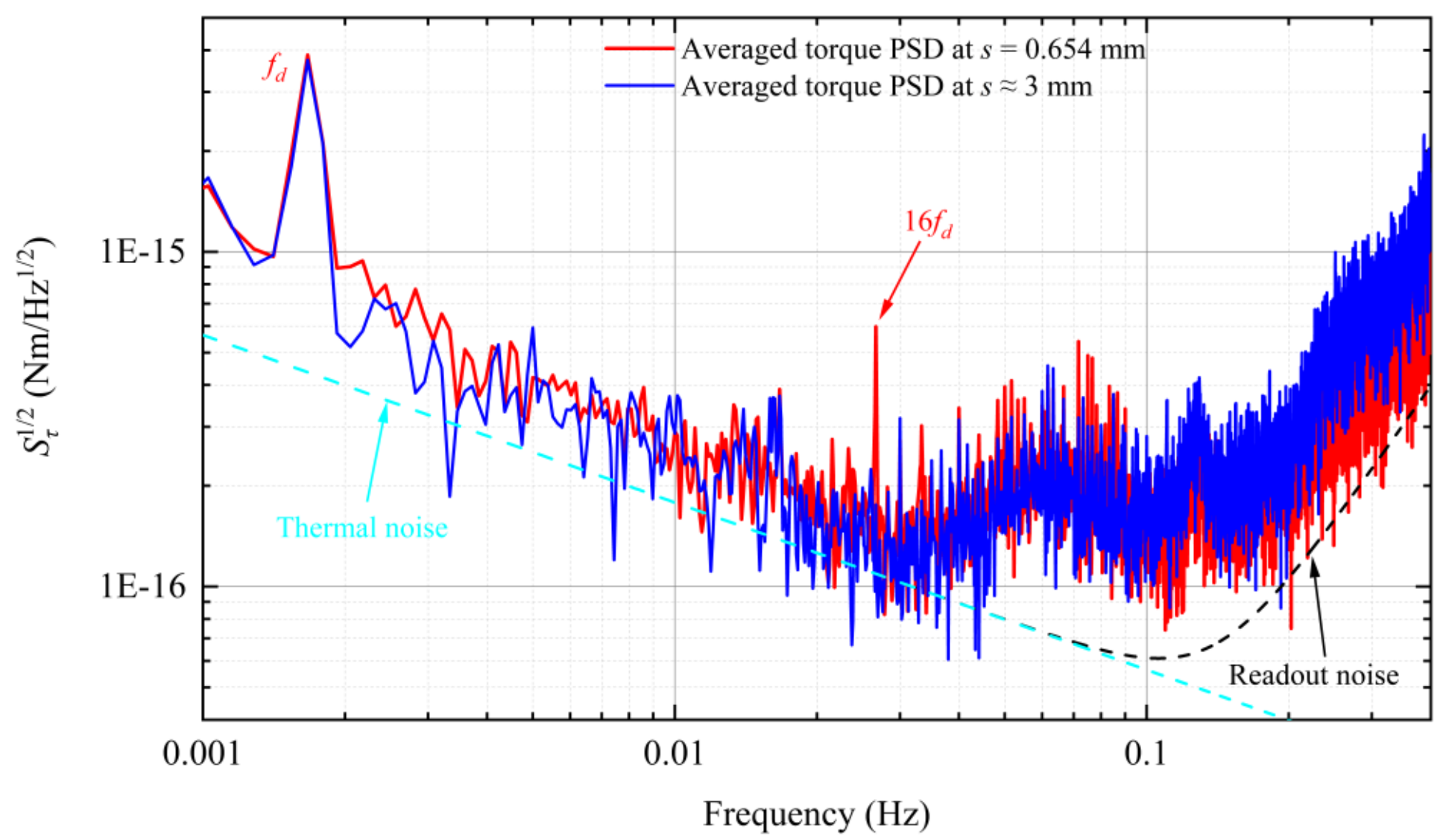

Figure 2(e): Comparison of averaged torque PSD at separations of 0.654 mm and 3 mm. At s = 0.654 mm, a distinct gravitational signal is clearly observed at $16f_d$. Conversely, at the larger separation of $s \approx 3$ mm, the Newtonian gravitational torque at $16f_d$ is theoretically expected to fall below the torsion balance noise floor, rendering it undetectable in the power spectrum. The experimental results confirm this theoretical expectation. This control test further corroborates the reliability of the close-separation measurements.

The measurement confirms this expectation: a prominent signal appears only at the drive fundamental frequency f_d, with a magnitude consistent with the close-separation result, while no discernible signal is present at $16f_d$. This control test further

corroborates the reliability of the close-separation measurements. In the frequency band above 30 mHz, some random peaks are likely attributable to membrane frame vibrations induced by the rotary stage or seismic noise, which represents an area requiring improvement in subsequent experiments. This indicates that, at the noise level of the torsion pendulum, the effect from the potential $16f_d$ fluctuations in the capacitive bias can be negligible.

(5).Loopholes such as this are not independently closed. Perhaps the authors rely on the consistency of the inferred value of G to bolster some confidence (but see next), but presumably, the point of these independent measurements is to act as an independent check; this they do not do yet.

We sincerely thank the reviewer for this excellent question, which touches a fundamental challenge inherent in all absolute measurement experiments. In absolute measurements, beyond minimizing statistical uncertainties, it is crucial to identify and characterize all possible systematic errors within our current understanding and capabilities. Naturally, some error mechanisms remain incompletely understood and require further investigation, while others necessitate additional modulation experiments to quantify their impacts. In our previous G-measurement experiments employing two distinct methods (Nature 560, 582-588, 2018), we dedicated substantial efforts to comprehensively evaluate various error sources, ultimately achieving a relative uncertainty of 0.00116% (11.6 ppm). This extensive work has provided us with valuable experience and methodologies.

In the present study, we selected small-mass objects as both test and source masses. Consequently, the reduced test and source mass diminishes the gravitational signal strength. Therefore, the measurement target for gravitational interactions (or the gravitational constant G) between small masses is set at a relative uncertainty of about 5%, limited primarily by the thermal noise of the torsion balance system. Although this result is not competitive in reducing uncertainty compared to our previous G-

measurement, it remains scientifically valuable for exploring gravitational phenomena at smaller mass scales.

Drawing upon our accumulated experience and methodologies, in addition to the thorough investigation of systematic errors arising from electrostatic forces, magnetic fields, and temperature, we have relied on the following cross-validation measures to address potential systematic errors that may not have been explicitly considered:

(a) Gravitational measurements at multiple separations. As shown in Fig. 3(a) of the main text, we measured the gravitational interaction between two silicon wafers at five different surface separations ranging from 0.654 mm to 1.554 mm. All results are consistent with the Newtonian gravitational law, which can be regarded as mutual validation among the measurements.

(b) Far-distance control experiment. As shown in Fig. 2(e) of the main text, we performed a control measurement at a larger surface separation ($s \approx 3$ mm). At this distance, the Newtonian gravitational torque at $16f_d$ is theoretically below the noise floor of the torsion balance system and should be unresolvable in the power spectrum. The measurement confirmed this expectation — no signal was observed at $16f_d$. This control experiment demonstrates that, within the noise level, any disturbance at $16f_d$ is negligible, further validating the reliability of the near-distance measurements.

(c) Supplementary experiments at different driving frequencies. Following the reviewer's suggestion, we conducted supplementary measurements at different turntable driving frequencies. The results confirm that the measured gravitational signal is independent of the modulation frequency (Extended Data Fig. 9). Admittedly, we only tested two frequencies; in the future, with improved instrument performance, verification at additional frequencies can be carried out.

(d) Independent re-measurement of geometric parameters. During the experimental preparation phase, all key geometric dimensions and relative positions of critical components were independently measured by different experimenters, providing additional cross-validation to rule out systematic errors introduced by

individual human factors.

(e) Repeated calibration of key system parameters. Critical parameters such as the electrostatic control coefficient β and the electrostatic stiffness k_e were periodically recalibrated throughout the experiment. The results from different calibration sessions are consistent within their respective uncertainties, and the final adopted values are weighted averages of multiple calibrations (see Extended Data Figs. 4 and 6). This procedure ensures that the system parameters are continuously monitored throughout the entire experimental period.

Of course, as the reviewer rightly noted, we cannot guarantee that no other unaccounted-for systematic errors exist. Nevertheless, the aforementioned thorough assessment of systematic errors and cross-validation experiments give us strong confidence in the reliability of our results. We sincerely thank the reviewer for the excellent suggestions, which have set a higher standard for our future research.

(6).But even the inferred G value has a blind-spot: how is the mass density of the source/test masses ascertained? (Indeed it seems like their density uncertainties are treated differently; why?)

Thanks to the reviewer. The silicon wafer (the test/source masses) density value represent an average calculated from multiple samples cut from the same wafer used for both the torsion pendulum and source masses, determined by measuring their masses and dimensions. We have added a footnote below the Table 1 about the mass density.

(7).There is no control experiment on the rotation frequency f. For example:

(a) a sequence of measurements where f is varied, and the inferred gravitational coupling is shown to be frequency independent

(b) or measured amplitudes of the other gravitational harmonics

(c) or measured phase of the 16f signal relative to the known phase from the source

mass encoder; it would seem like the error signal already contains this information, which the authors could analyze.

We thank the reviewer for their excellent suggestions. In this experimental design, the amplitude of the Newtonian gravitational torque at the $32f_d$ harmonic is approximately 0.2 aNm, which is approaching the thermal noise limit of the torsion balance system in this experiment. Consequently, it is difficult to obtain meaningful results through harmonic analysis. We have adopted the reviewer's other suggestion to change the driving frequency and repeat the experiment.

Thanks to the reviewer for the insightful suggestion. Fortunately, our experimental apparatus remained operational. We increased the rotation period of the turntable from 600 s to 3600 s, which corresponds to a gravitational signal frequency of 4.444 mHz (16/3600) at the 16th harmonic of the modulation frequency (f_d). The rationale for selecting this frequency is twofold: 1) **Sensitivity consideration:** Based on the sensitivity characteristic curve of our torsion balance, which exhibits a $1/f$-type noise profile, the noise level at this frequency is higher than that at the previously selected frequency. Nevertheless, it remains within an acceptable range. 2) **Frequency separation:** We intended to maximize the separation between the new frequency and the currently employed one ($16f_d$= 26.667 mHz), thereby enabling the observation of any potential frequency-dependent phenomena.

We conducted 15 sets of measurements with the surface spacing of 0.654mm between the test mass and source mass, each involving 13 full rotations of the turntable and taking 13 hours. Following the signal extraction method described in Supplementary Information Section 1, we obtained a gravitational signal of τ_2 = 11.41(46) aNm at the $16f_d$ (4.444 mHz) frequency, where the uncertainties represent 1σ statistical uncertainties only. Within the 1σ uncertainty range, this result is consistent with our previously measured gravitational signal τ_1=11.16(27) aNm at $16f_d$ (26.667mHz). The increased noise at the new frequency is primarily due to the slower experimental frequency, which leads to greater *1/f* noise. The results indicating that no

frequency-dependent phenomenon was observed. We have incorporated these findings into the main text and the Extended Data Figure 9. We sincerely appreciate the reviewer's valuable suggestions.

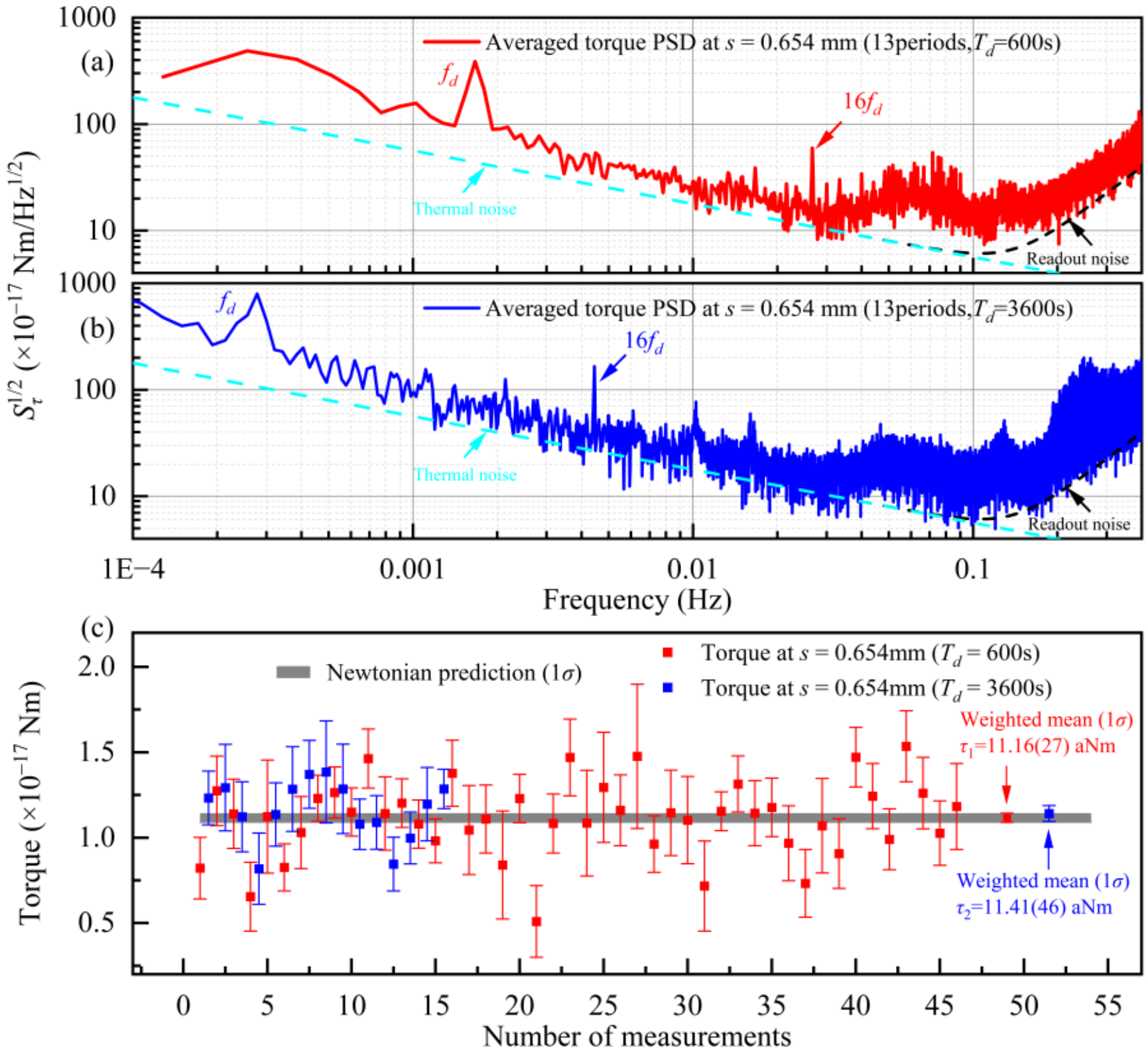

Extended Data Figure 9. Comparison of measured gravitational torques for the source mass disk rotating at two different periods (600s vs 3600s) at s = 0.654 mm. (a) & (b) Average torque power spectral densities (PSDs) of the torsion balance from multiple independent measurement segments (each containing 13 rotation periods) at s = 0.654 mm for rotation periods of 600 s and 3600 s, respectively. Distinct signals were observed at frequency 16fd (26.667 mHz and 4.444 mHz, respectively). (c) Torque amplitudes at 16fd frequency extracted from data segments using the time-domain orthogonal decomposition method (Supplementary Information Section 1). Each point represents the average torque over 13 rotation periods. At the signal frequency of 26.667 mHz, 46 measurements yielded a weighted mean τ_1 = 11.16(27) aNm, while at 4.444 mHz, 15 measurements yielded τ_2 = 11.41(46) aNm. The uncertainties represent 1σ statistical uncertainties only. The gray band indicates the theoretical Newtonian torque of 11.17(26) aNm at this separation distance. The elevated noise level at 4.444 mHz compared to 26.667 mHz primarily results from increased *1/f* noise characteristics of the torsion pendulum at lower frequencies. All measurements remain consistent within 1σ uncertainties, confirming the frequency independence of the gravitational measurement results.

(8).What about translation motion of the pendulum-membrane gap? The authors measurements appear to only bound the tilt motion, whereas translation motion remains unbounded, and could definitely cause a spurious signal.

We sincerely thank the reviewer for this insightful suggestion. In our previous work, we indeed only measured the tilting motion of the shielding membrane in two orthogonal directions using an autocollimator during turntable rotation. However, given that the membrane and its frame were mounted on a translation stage, our assumption that the membrane exhibited no pure translational motion was scientifically inadequate.

To address this limitation in the revised manuscript, we have specifically employed an ultra-precision interferometer (Model: HUE-XL, Harbin Institute of Technology; accuracy: ~20 pm) to measure the membrane's translational motion comprehensively. For rigorous comparison, we conducted measurements under both stationary and rotating turntable conditions. The experimental results reveal that during turntable operation, the displacement noise of the membrane's translational motion deteriorates compared to the stationary condition in the frequency band above 40 mHz. While in the frequency band below 40 mHz, the noise levels remain comparable between moving and stationary states. Notably, at the critical frequency of $16f_d$ (=16.67 mHz), we detected no significant translational motion, with displacement noise remaining below 0.324 nm.

Based on the electrostatic coupling coefficient between the torsion pendulum and the membrane (k_e=19.4(2.7)pNm/rad) and the effective lever arm (L_{eq} = 23.694(3) mm), we calculated that the electrostatic torque noise introduced by the membrane's translational motion is $\delta\tau_e = \frac{k_e \cdot \delta d}{L_{eq}} \leq 0.27$aNm. This error will be incorporated into the total torque error budget. Additional experimental descriptions have been incorporated into Extended Data Fig. 6.

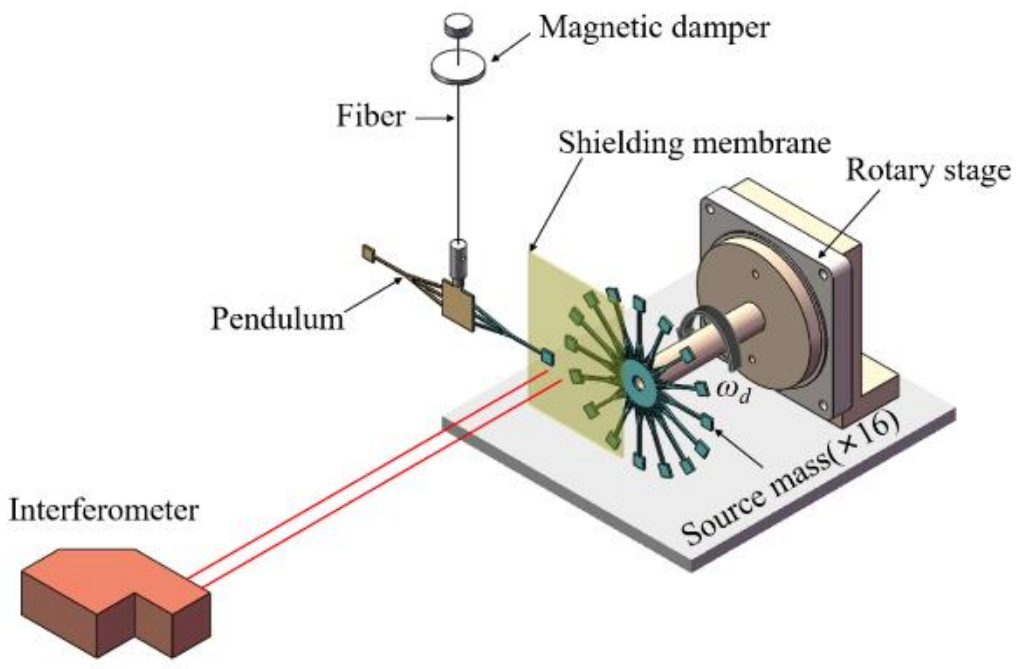

Extended Data Fig.6 (c). Schematic of shielding membrane displacement measurement using ultra-precision laser interferometry.

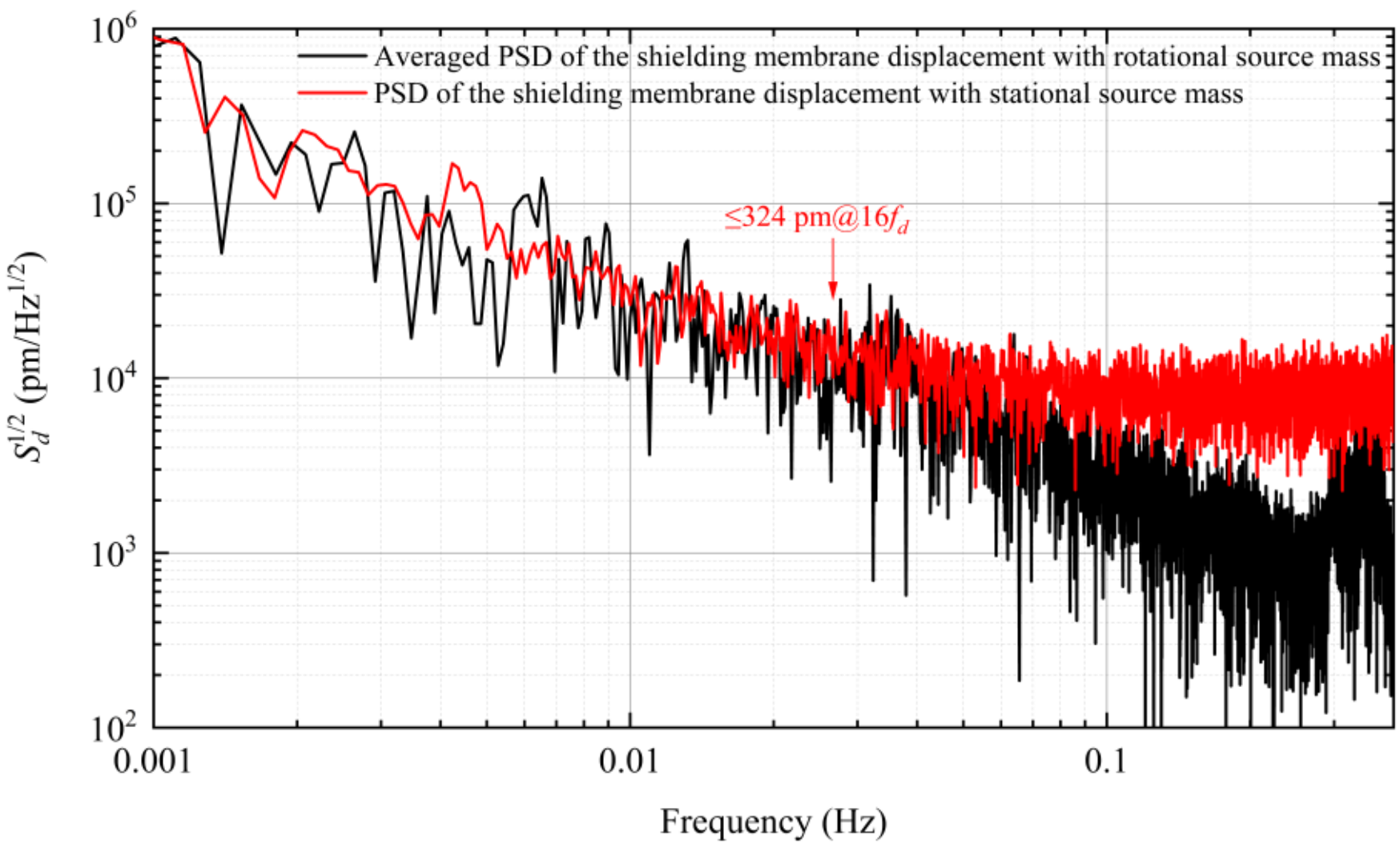

Extended Data Fig.6 (d). Power spectral density (PSD) of shielding membrane translational displacement: turntable stationary (black) vs. rotating (red). At the critical frequency 16f_d (=16.67 mHz), confirming no significant translational motion under displacement noise of less than 0.324 nm.

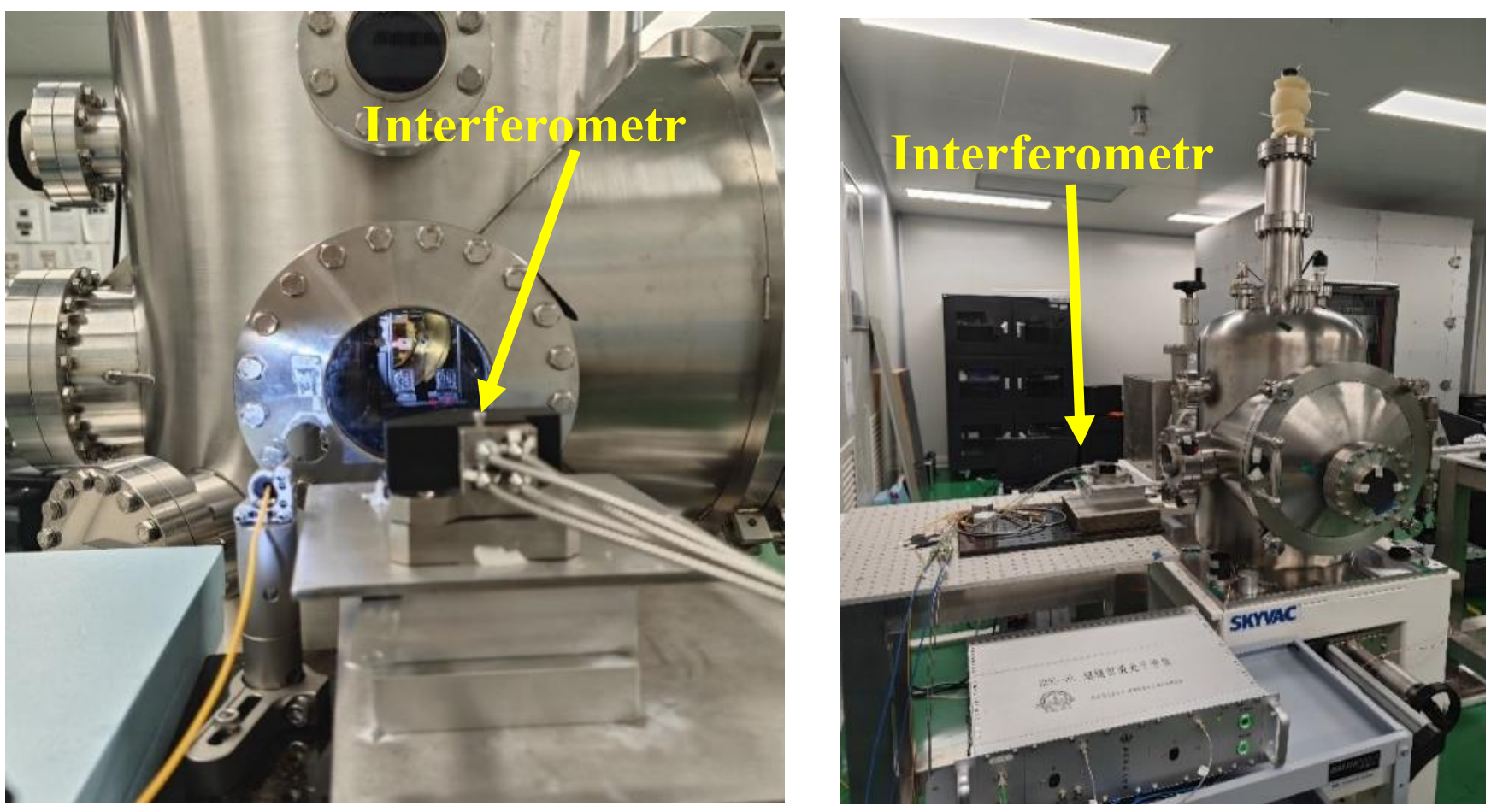

Figure R8. Photograph of shielding membrane displacement measurement using ultra-precision laser interferometry.

(9).How is the source mass electrically neutralized?

We thank the reviewer for their comments. In our experimental setup, the source mass is connected to the turntable housing via aluminum connecting rods, with the turntable housing electrically grounded. The question of whether the source mass maintains complete electrical neutrality remains undetermined. To mitigate the disturbance deduced by contact potential differences between the source mass and the torsion pendulum, a gold- plated shielding membrane was installed between them. Additionally, the entire source mass assembly, including the turntable, is enclosed within an aluminum box for electrostatic shielding, as illustrated in the figure below.

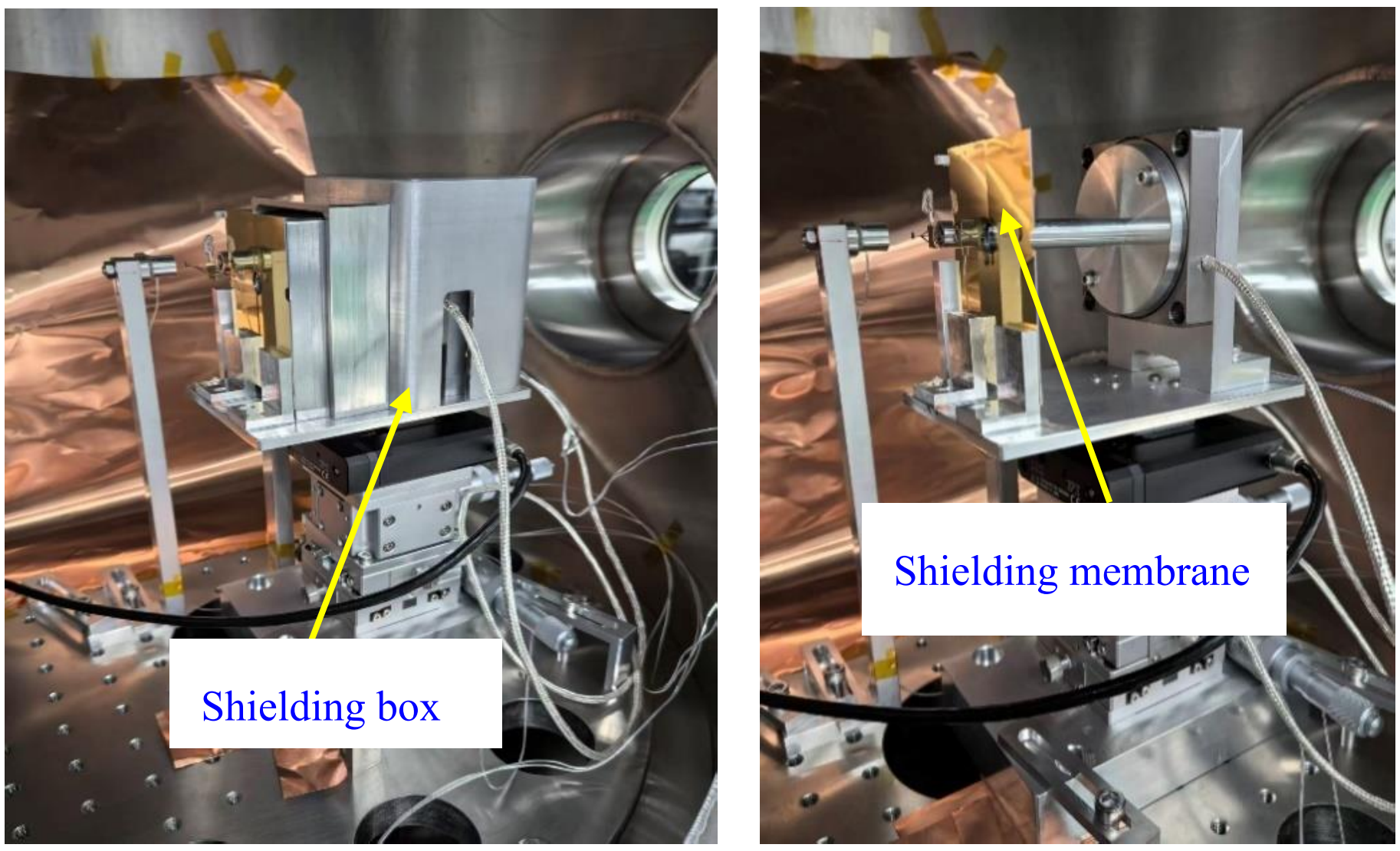

Fig. R9. Photograph of the experimental setup showing the turntable, connecting rods, shielding membrane, and shielding box.

(10).The fabrication and dimensional precision achieved here for the test and source mass are very important details, and in fact one of the main technical advances. But these details are missing in the paper, without which, the reader's confidence in the error estimates arising from dimensional uncertainty is low. So I suggest:

(a) showing a photograph of the test and source mass in Fig. 1 (perhaps with additional angles in the extended data)

Thanks to the reviewer. Additional quality inspection photographs and supplementary images of the test and the source mass have been incorporated into the

Extended Data figure 1.

(b) adding a section in a supplementary information detailing the full fabrication details of the device in replicable form.

Thanks to the reviewer. Silicon wafer etching is a mature semiconductor manufacturing process. Below is a brief introduction, accompanied by a recommended reference book (Editors : Reza Ghodssi, Pinyen Lin, MEMS Materials and Processes Handbook, Springer New York Dordrecht Heidelberg London. 2011. https://link.springer.com/book/10.1007/978-0-387-47318-5). We have included some processing details and some photographs of the processing process in the Supplementary Information section 3.

(11).Extended Data Figure 9 would benefit from being moved to the main text, and the message surrounding it explicated further there (as well as in the abstract and introduction).

Thanks to the reviewer's excellent suggestion. Extended Data Figure 9 has been moved to the main text, and corresponding introductions have been added to the abstract and introduction.

Response to the reviewers' comments (second round)

Dear Reviewers,

We sincerely thank you for the time and thoughtful consideration you have devoted to reviewing our manuscript. Your constructive criticism and insightful suggestions have been invaluable in strengthening this work. Below, we provide a detailed point-by-point response to all comments; our replies are indicated in **blue**. We are deeply grateful for your expert guidance throughout this review process.

Reviewer #1

I thank the authors for considering my and the othor reviewers' comments. Upon examination, the revised manuscript appears to substantially addressed most of concerns that were raised

Remaining concerns:

1. It is unclear what "General relativity (GR) and the standard model (SM) are fundamentally incompatible" means. This seems to be an overstatement.

Thank you for the reviewer's comments. The statement that "General relativity and the standard model are fundamentally incompatible" is widely accepted in the physics community. This represents one of the most fundamental challenges in modern physics and serves as a frontier direction in theoretical physics research, providing motivation for the study of quantum gravity.

2. I don't quite follow the logic of "... Gravitational measurements where both the source mass and the test mass are in the small-mass regime remain relatively scarce. Consequently, it remains an open question whether the gravitational law still follows Newtonian gravitation when the objects become sufficiently small or even enter the quantum regime." Theoretically, the 1/r^2 is well-motivated and expected. What small-mass gravitational measurements provide is an opportunity to probe deviations from this expectation, rather to solve an open question, per se.

We appreciate the reviewer's valuable suggestions. We have revised the expression as *"Gravitational measurements where both the source mass and the test mass are in the small-mass regime remain relatively scarce. Consequently, whether gravitational force deviates from Newton's inverse-square law when the objects become sufficiently small or even enter the quantum regime remains a question worthy of investigation."*

3. In the outlook "To observe gravitation-induced quantum effects, both the source mass and the test mass should ideally fall within the range of 10^(-15)to 10^(-13)kg." seems

to be referring to a particular set of proposals. If so, please cite, or all address the narrow range.

We appreciate the reviewer's valuable suggestions. Suggestions based on two experimental schemes below, to observe gravitation-induced quantum effects, both the source mass and the test mass should ideally fall within the range of 10^{-14} to 10^{-12} kg. This range represents a compromise between competing requirements: sufficient gravitational coupling strength (favored at higher masses) and adequate quantum coherence times (favored at lower masses).

[1] Marletto, C. & Vedral, V. Gravitationally induced entanglement between two massive particles is suffcient evidence of quantum effects in gravity. *Phys. Rev. Lett.* **119**, 240402 (2017).

[2] Bose, S. *et al.* Spin entanglement witness for quantum gravity. *Phys. Rev. Lett.* **119**, 240401 (2017).

4. The authors have only cited levitated experiments, whereas I suggested highlighting a broader ecosystem the including torsion pendula (similar to the authors') and other tethered optomechanical systems.

We appreciate the reviewer's valuable suggestions regarding the contextualization of our outlook within the broader ecosystem of mg-scale experiments. Following this recommendation, we have optimized the expression as *"In recent years, a growing number of experimental platforms have pursued the reduction of test-mass scales in the quest for gravitational interaction detection between objects in the quantum regime. These include torsion pendulum techniques, optical and magnetic levitation schemes, as well as cavity optomechanical systems and so on. The continued advancement of these approaches, each operating across different but increasingly overlapping mass regimes, holds promise that gravitational interactions between quantum-state objects may be observed in the foreseeable future."* In addition, we add some new reference.

[1] Agafonova, S., Rosselló, P., Mekonnen, M. *et al.* One-milligram torsional pendulum

toward experiments at the quantum-gravity interface. *Commun Phys* **9**, 80 (2026).
[2] Guan, S.-G., Cheng, Y.-B., Sun, J., Duan,Z.-L. & Le, J.-X. All-optically operated atto-newton force sensing with a centimeter-milligram-scale torsion pendulum. *Phys. Rev.Lett.* **136**, 063603 (2026).
[3] Millen, J., Monteiro, T. S., Pettit, R. & Vamivakas, A. N. Optomechanics with levitated particles. *Reports on Progress in Physics* **83**, 026401 (2020).

Reviewer #2

Dear authors,

Thank you for considering all my comments and suggestions. Especially, I am gratefully for conducting an additional experiment at another frequency, which showed consistency. I agree with the revised manuscript and recommend a publication. I finally have a few minor comments. It is up to the authors whether they address them or not.

In main manuscript:

1) Abstract (also within the main text): Why do you give a 2-sigma uncertainty for the uncertainty of 46 aN, while the uncertainty for G is given as 1-sigma?

We appreciate the reviewer's valuable suggestions. The primary reason for the appearance of 1-sigma and 2-sigma levels is influenced by international conventions. When discussing the constraints on Yukawa-type forces from gravitational measurement precision, it is usual practice to use 2-sigma levels of measurement results to provide constraints on parameters. However, for measurements of the gravitational constant G, it is conventional to present results using 1-sigma levels.

2) P.7, right column: "… (4.8% relative error), …" This should be "relative uncertainty". If you know that this is an error, then you can correct it. Ana dadd a space between 4.8 and %, as % is teated as a unit.

We appreciate the reviewer's valuable suggestions, we have revised.

In Supplementary Information

1) 1.1 Data preprocessing methods: These should be negligible contributions, but can you mention which kind of voltmeter you have used, and whether it was calibrated beforehand? Did you consider the sinc-error? What about clock stability?

We appreciate the reviewer's valuable suggestions. The control voltage is generated by a PXI Multifunction I/O Module (National Instruments PXI-6289) featuring a 16-bit DAC resolution. Its output voltage offset was characterized using a high-precision digital voltmeter (Keysight 3458A). Since this experiment requires only the precise determination of the *variation* in output voltage, rather than the absolute voltage level, this offset is not of primary concern. In the formal experiment, the modulation signal amplitude at the target frequency (~26.667 mHz) is designed to be approximately 0.33 mV. This amplitude was calibrated using the high-precision digital multimeter, with a calibration accuracy better than 3.3 μV. Consequently, this introduces a relative calibration error of approximately 1% to the measured torque values reported in this work.

The sinc-error is determined by the sampling time $\Delta t = 1\ \mathrm{s}$ and the signal frequency $\omega_s = 0.168\ \mathrm{rad/s}$ (corresponding to the signal period T_s = 37.5 s), that

$$\frac{\sin(\omega_s \Delta t)}{\omega_s \Delta t} = 0.995.$$

It means the calculated amplitude of the modulation signal will attenuate by 0.5%. The timing accuracy of the voltage output module is 50 ppm of sample rate, and that of the control computer is similar. For the gravitational modulation period of 37.5 s and the data segment with a duration of 7800 s, the clock uncertainty contributes a relative error of 0.07% in calculating the amplitude according to the Fourier series. On the other hand, the closed-loop transfer function is calibrated by the open-loop stiffness k_f of the suspension fiber, which was derived from the free oscillation of the torsion pendulum by $k_f = I\,\omega^2$. It contributes the relative uncertainty $\frac{\delta k_f}{k_f} = 2\frac{\delta\omega}{\omega} = 100\ \mathrm{ppm}$ for the 50-

ppm time uncertainty. As a result, clock stability contributes the relative error less than 0.1%, and can be neglected.

2) 1.5 Conversion to torque and error propagation: For the electrostatic control coefficient \beta, can you please write the units to each term, not just in parenthesis after the equation.

We thank the reviewer for this helpful suggestion. In the Supplementary Information, we have now specified the units for each term in the expression for $\beta(U)$ (as highlighted in Supplementary Figure 5).

p.5, l.113: the terms (\tau) below the square root must be squared (not the \cos and\sin)

We thank the reviewer for catching this typo. We have corrected the error in Supplementary Information Section 1.5. The terms $\tau_{\cos}$ and $\tau_{\sin}$ are now properly squared under the square root.

3) 2.4 Measurement of …, p.7, l.162: “Using a vision measuring machine …” Throughout the text you use the term “Video measuring machine”. I this a different system?

We thank the reviewer for pointing out this inconsistency. We have now unified the terminology to "Video Measuring Machine" throughout the Supplementary Information and main text.

l. 163: “… is measured to be 0.268 mm” Can you give here an uncertainty, as you have done for the other measurements?

We thank the reviewer for the suggestion. The spacing was measured using the same Video Measuring Machine (QVP5040T, accuracy: ±3 μm) and is now reported as 0.268(3) mm in Supplementary Note 2.

Reviewer #3

The authors have thoroughly addressed all the questions and comments I had raised during the previous round of review.

We sincerely thank the reviewer for the time and efforts devoted to reviewing our manuscript, and for the positive feedback. We are glad that our revisions have fully addressed all the concerns raised during the previous round of review.

In “Test of the gravitational interaction between milligram masses using a miniature torsion pendulum,” the authors describe a miniature Cavendish-type experiment in which the gravitational force between two lithographically defined source masses is measured and confirmed to agree with the Newtonian inverse square law. The absolute mass (~ 8 mg) of the source masses is 10-times smaller than a similar experiment conducted by Westphal et. al. 2021 (reference [1]) and represents important step towards Planck mass-scale experiments (~0.1 mg) proposed to test the quantum nature of gravity.

The reported results will garner significant interest and are deserving of being published in Nature Communications, in my opinion. The authors have been developing variants of the apparatus for over a decade. While I am not an expert in this measurement, I found its treatment authoritative.

That said, I have some concerns about presentation of ideas and data in the manuscript that I believe should be addressed before publication. These are listed below, followed by relatively minor comments.

1. Textual overlap with [1]
Several passages in the abstract and introduction contain significant overlap with [1], either in wording or narrative structure. For instance, in the abstract:

(Westphal) “Because quantum coherence is easily lost for increasing system size, it is important to isolate gravity as a coupling force for as small objects as possible.”
(An) “Since the quantum coherence is easily lost with the increasing size or mass of the system, it is essential to characterize gravity for as small objects as possible.”

and

(Westphal) “Our results extend the parameter space of gravity measurements to small, single source masses and low gravitational field strengths.”
(An) “Our results extend gravity experiments to the regime of small gravitational sources masses at the atto-newton level, and demonstrate the potential of going considerably smaller”

While some overlap is to be expected, the authors are encouraged to revise the abstract and introduction in their own language (not just rearranging words), and to be vigilant against copying.

2. Unclear motivation and relation to quantum experiments
In my opinion, the abstract in introduction do not make it sufficiently clear why this experiment is important, especially in relation to quantum experiments. For example

(1) the abstract begins with a tautology: *Probing the interplay between quantum mechanics and gravity… offers a… pathway to explore the quantum nature of gravity*,

(2) the connection between small mass and quantum gravity is missing in the abstract: *“What is lacking are experiment that probe the mutual gravitational interaction between mesoscopic or microscopic objects where quantum effects dominated. Here, we report a direct measurement of the gravitational interaction between two 7.6-mg silicon wafers using a miniature torsion pendulum…”*

(3) the pivotal “however” statement in the intro is a non-sequitur (false/unsupported contrast): *However, measurements of gravity from classical sources in laboratory table-top settings is in contrast with the increasing attention on probing gravitational interactions originated from quantum state of source masses.*

These examples are part of a larger pattern in which key ideas are presented in proximity so as to imply their connection, instead of explicitly stating what the connection is. The authors are encouraged to revise the abstract and introduction to more concretely motivate the work, particularly its connection with quantum gravity tests. If need be, diminishing the focus on quantum tests would not be an issue, IMO.

3. Data presentation in Fig. 2

The experimental data presented in Fig. 2 is compelling as a demonstration that the authors have characterized their torsion pendulum and feedback control system; however, it does not clearly highlight the signal of interest: a small coherent tone at $16f_s$ buried in the broadband noise spectrum in panels (d,e). By contrast, in Fig. 2 of Westphas [1], the coherent signal at f_s and $2f_s$ rises visually above the noise and may be directly quantified in both displacement and force units, by comparing to the left/right plot axis.

The quality of the processed data in Fig. 3 suggests that a cleaner visualization of the raw experimental data is possible. The authors are encouraged to do so in Fig. 2, possibly by using magnified insets in panels (d) and (e), or by showing representative noise spectra in Fig. 3.

4. Technical overload in Methods section

The paper is written in the style of a traditional metrology paper, with great attention to technical detail. This is fine and, for posterity, important; however, to improve readability for non-specialists (a purpose for publishing in Nature Communications), it is advisable that the authors buttress technical discussion with explanations of what details are most important and why. The *Methods* section stands out as unusually technical for a Nature paper. Much of this content could in principle be off-loaded to an appendix. If the authors are intent on keeping it in the main text---which is reasonable, given that this *is* a precision metrology paper---I recommend sub-sectioning and/or prefacing each paragraph with a “why this important” sentence, similar to how the *Systematic effects* section is constructed. The authors might in any case consider lightening and/or moving some technical details to the appendix.

Minor comments in rough chronological order (# = line #)

(general) The text is adequately written, especially the technical sections, but could use a once over for grammatical correctness. Some examples are given below. They are not exhaustive.

(abstract) See comments 1 and 2 above.

(introduction, especially paragraph 1) See comments 1 and 2 above.

(47, col 1) “*mass used in the previous test [1]*” implies [1] is the only test preceding the the authors’. If this is correct, then fine. If not, then “the” should be replaced with “a”, or the sentence reconstructed.

(49, col 1) *“...small gravitational masses at the atto-Newton level..”* Since masses don’t have units of Newtons, this statement is unclear. Consider revising.

(57, col 1) *“GR and the SM… have so far passed all experimental tests… However, the incompatibility of the two theories provides significant motivation”* The second sentence is premised on the unstated claim that GR and SM are incompatible. This should be stated explicitly with support from a reference.

(58, col 2) *“50 times enhanced than”* → *“50 times enhanced over”*

(65, col 2) *“a schematic view of the new experimental design”* Readers will not necessarily be familiar with the authors previous work, so “new” needs to be qualified or removed.

(67, col 2) *“Φ-shaped”* is unclear. Is Φ-shaped a well-known torsion pendulum geometry? An observational aside? Since the phrase is not used elsewhere, consider removing it.

(70, col 2) Consider cross-referencing *Table 1* when introducing device dimensions for the first time.

(93, col 2) *"The attractor is designed as "ferris-wheel-shaped" to ensure its structural strength"* Similar to the above comment, it's unclear why pointing out that the attractor looks like a ferris wheel adds useful information. The sentence implies that there is a connection between ferris wheels and structural strength. This may be case, but not obviously. Consider revising this sentence for concision and clarity.

(150, col 1) *"The rotation axis of the attractor is aligned to the rotary stage within 40 μm using an optical imaging instrument."* It is unclear what is meant by "optical imaging instrument." Consider stating.

(134, col 2) *"A 50-um-thick, gold-coated conducting membrane…"* Consider stating what the membrane substrate material is. Silicon nitride?

(184, col 1) Consider changing the section title *Method* to the (more typical) *Methods.*

(Methods section) See comment 4 above.

(196, col 1) *"…τ(t) is the external torque"* Does $\tau(t)$ include thermal noise? If so, consider stating so, or else consider including an additional thermal torque in Eq. 1.

(252, col 1) *"The 16ω_d Newtonian torque is calculated via numerical integration over separations from s = 0.654 to 1.554 mm"* It is unclear what is being numerically integrated over. Please clarify.

(240, Fig. 2 caption) "*The transformed torque PSD.."* It is unclear what "transformed" means. I believe the authors mean the "inferred" or "apparent" torque PSD.

(266, Table 1) Can the authors provide a reference or a footnote for how the density uncertainty, $2313.5 \pm 11.9\ \mathrm{kg/m^3}$ was obtained? (It seems reasonable, but oddly specific to a nonspecialist.)

(301, col 2) Can the authors provide a reference or commentary on how the "$\delta T < 2.1\ \mu$K temperature fluctuations at $16\omega_d$" was determined?

(Fig. 3) Minor comment: consider changing panel B red point label *"Measurement result of G"* to *Measured/Inferred/Calculated G*, since, as described in the text and caption, these are indirect measurements.

(351, col 2) For passage *"Beyond confirming Newtonian gravity… small masses may help bridge between… lab tests of classical gravity and future experiments designed to access regimes relevant to quantum coherence"* it would be helpful to have references supporting claims.

(361, col 1) "*However, this is not generally advantageous"* What does "this" refer to?

(Outlook) The outlook highlights strategies to improve the authors' apparatus (including reducing the fiber size and moving from metal-alloy to quartz) toward the long-term goal of Planck-mass experiments. This is interesting and appropriate; however, for a Nature audience, the authors might also consider contextualize their outlook within the broader ecosystem of mg-scale experiments and sensors aimed at precision gravity measurements---including torsion pendula (both lithographic and assembled), levitated systems, and optomechanical platforms---currently facing similar challenges and pursuing similar goals.
